# Comparison of the Effect of Endurance, Strength and Endurance-Strength Training on Glucose and Insulin Homeostasis and the Lipid Profile of Overweight and Obese Subjects: A Systematic Review and Meta-Analysis †

**DOI:** 10.3390/ijerph192214928

**Published:** 2022-11-13

**Authors:** Małgorzata Jamka, Aleksandra Makarewicz-Bukowska, Kamila Bokayeva, Angelika Śmidowicz, Jakub Geltz, Marta Kokot, Nina Kaczmarek, Agnieszka Żok, Victoria Kononets, Judyta Cielecka-Piontek, Edyta Mądry, Jarosław Walkowiak

**Affiliations:** 1 Department of Pediatric Gastroenterology and Metabolic Diseases, Poznan University of Medical Sciences, Szpitalna Str. 27/33, 60-572 Poznan, Poland; 2 Division of Philosophy of Medicine and Bioethics, Poznan University of Medical Sciences, Rokietnicka Str. 7, 60-806 Poznan, Poland; 3 Department of Natural Sciences Disciplines, West Kazakhstan Marat Ospanov Medical University, Maresyev Str. 68, Aktobe 030019, Kazakhstan; 4 Department of Pharmacognosy, Poznan University of Medical Sciences, Rokietnicka Str. 3, 60-806 Poznan, Poland; 5 Department of Physiology, Poznan University of Medical Sciences, Święcickiego Str. 6, 60-781 Poznan, Poland

**Keywords:** physical activity, exercises, overweight, obesity, glucose, insulin, cholesterol

## Abstract

The most effective type of training to improve cardiometabolic parameters in overweight subjects is unknown. This meta-analysis compared the effect of endurance, strength and combined training on glucose, insulin metabolism and the lipid profile of overweight and obese adults. The Cochrane, PubMed, Scopus and Web of Science databases were searched to identify randomised trials assessing the effect of training intervention on fasting and 2 h glucose and insulin levels, glycated haemoglobin (HbA1c), homeostatic model assessment of insulin resistance (HOMA), C-peptide, total cholesterol (TC), low- (LDL-C) and high-density lipoprotein cholesterol and triglycerides (TG). Forty-six studies were included showing that endurance training more favourably reduced HbA1c (*p =* 0.044), and LDL-C (*p =* 0.021) than strength training. Endurance-strength training more effectively decreased glucose (*p =* 0.002), HbA1c (*p =* 0.032), HOMA (*p =* 0.002), TC (*p =* 0.039), LDL-C (*p =* 0.046), HDL (*p =* 0.036) and TG levels (*p =* 0.025) than strength training. Combined training significantly reduced the HOMA index (*p =* 0.009) and TG levels (*p =* 0.039) compared with endurance training. Endurance and endurance-strength training have a more favourable effect on glucose and insulin homeostasis and lipid profile than strength training in overweight and obese adults. However, the results from this meta-analysis should be interpreted cautiously due to significant heterogeneity among included studies.

## 1. Introduction

Obesity is a major public health problem associated with many serious health conditions. Recent data showed that almost two billion adults are overweight globally, while more than 670 million are obese [1]. Obesity significantly increases the risk of the development of several diseases, as excessive body weight is associated with elevated blood lipids, lipoproteins, cholesterol and insulin resistance. Consequently, obesity increases the risk of type 2 diabetes mellitus [2], may cause dyslipidaemia [3] and contributes to atherosclerosis and cardiovascular diseases [4]. Obesity also increases the risk of developing arthropathy [5], some cancers [6], non-alcoholic fatty liver disease [7] and several other conditions, frequently reducing the overall life expectancy and negatively affecting the quality of life. Therefore, prevention and treatment of obesity are one of the main public health challenges [8].

Noncommunicable diseases related to excessive weight and obesity are largely preventable [9]. Indeed, exercise intervention for overweight and obese adults is one of the effective methods to prevent and treat obesity and reduce the risk of developing concomitant diseases [10,11]. Endurance (aerobic) training has been recommended for obese subjects since it may decrease body weight and fat mass (FM), as well as improve cardiometabolic markers [12,13,14,15]. However, strength (resistance) training has a similar effect [16] or may be even more effective in improving cardiometabolic or anthropometric parameters than endurance training [17,18]. The beneficial effects of combined (endurance-strength) training on cardiometabolic markers compared with endurance training or strength training have also been reported [19,20], while some studies found no differences between training programmes [21,22].

A recent network meta-analysis evaluated the efficacy of five exercise modalities (including endurance, strength and combined training) on cardiometabolic parameters in overweight and obese subjects and found that hybrid training was the most effective in elevating high-density lipoprotein cholesterol (HDL-C) levels and reducing fasting glucose concentrations, combined training was the most effective in reducing low-density lipoprotein cholesterol (LDL-C) levels and lowering fasting insulin concentrations and homeostatic model assessment of insulin resistance (HOMA) index, while interval training was ranked the best in reducing triglycerides (TG) concentrations and glycated haemoglobin (HbA1c) levels. However, the meta-analysis only included studies performed on participants aged from 18 to 64 years, with no diagnosed comorbidities or any noncommunicable diseases, and did not exclude subjects who also received dietary interventions. Moreover, the effect of training on 2 h glucose and insulin levels and C-peptide was not assessed [23]. Therefore, this systematic review aimed to compare the effect of endurance, strength and combined training on glucose and insulin metabolism and lipid profile in overweight and obese adults (including subjects with and without obesity-related comorbidities) who did not receive dietary consultation or nutritional intervention.

## 2. Methods

### 2.1. Protocol and Registration

This study was performed according to the preferred reporting items for systematic reviews and meta-analyses (PRISMA) [24] and Cochrane guidelines [25] and was registered with the International Prospective Register of Systematic Reviews (PROSPERO; registration number: CRD42020183252, date of registration: 11 July 2020, see Supplementary Material, Table S1) [26].

### 2.2. Information Sources and Search Strategy

The Cochrane, PubMed, Scopus and Web of Science databases were searched for articles written in English and performed on humans. The following search strategy was implemented in each database:

Cochrane (1908–2021):

#1—(obesity OR overweight [Title, Abstract, Keyword]);

#2—(endurance training OR strength training OR exercise [Title, Abstract, Keyword]);

#3—#1 AND #2;

#4—#3 AND (Trials AND English [Filter]).

PubMed (1966–2021):

#1—(obesity OR overweight [MeSH Terms]);

#2—(endurance training OR strength training OR exercise [MeSH Terms]);

#3—#1 AND #2;

#4—#3 AND (humans AND English [Filter]).

Scopus (1960–2021):

#1—(obesity OR overweight [Article title, Abstract, Keywords]);

#2—(endurance training OR strength training OR exercise [Article title, Abstract, Keywords]);

#3—#1 AND #2;

#4—#3 AND (Article AND English [Filter]).

Web of Science (1900–present):

#1—(obesity OR overweight [Topic]);

#2—(endurance training OR strength training OR exercise [Topic]);

#3—#1 AND #2;

#4—#3 AND (Article AND English [Filter]).

Manual searches of the bibliography of all studies included were also performed to identify other relevant papers. The search process was conducted between July 2020 and June 2021.

### 2.3. Eligibility Criteria

The inclusion criteria were as follows: types of studies: randomised trials; language: articles written in English; population: overweight and obese adults; intervention: endurance vs. strength training or/and endurance vs. combined training or/and strength vs. combined training where the subjects were instructed not to change dietary habits; duration of the intervention: at least two weeks; outcomes: glucose and insulin metabolism parameters (fasting glucose levels, fasting insulin levels, two-hour glucose levels (after oral glucose tolerance test (OGTT)), two-hour insulin levels (after OGTT), HbA1c, C-peptide levels and HOMA) and lipid profile (total cholesterol (TC), LDL-C, HDL-C and TG levels).

The exclusion criteria included: types of studies: case–control, case-series, case-report, cohort studies, conference reports, cross-sectional studies, editorial letters, observational studies, uncontrolled trials, studies available only as abstracts and studies performed on animal models; population: children, adolescents, pregnant and women during lactation, subjects living in non-public (closed-type) houses; subjects who cannot free decided on their dietary habits; intervention: studies in which exercise intervention was combined with dietary consultation or intervention or dietary supplementation.

### 2.4. Study Selection

**Figure 1:**
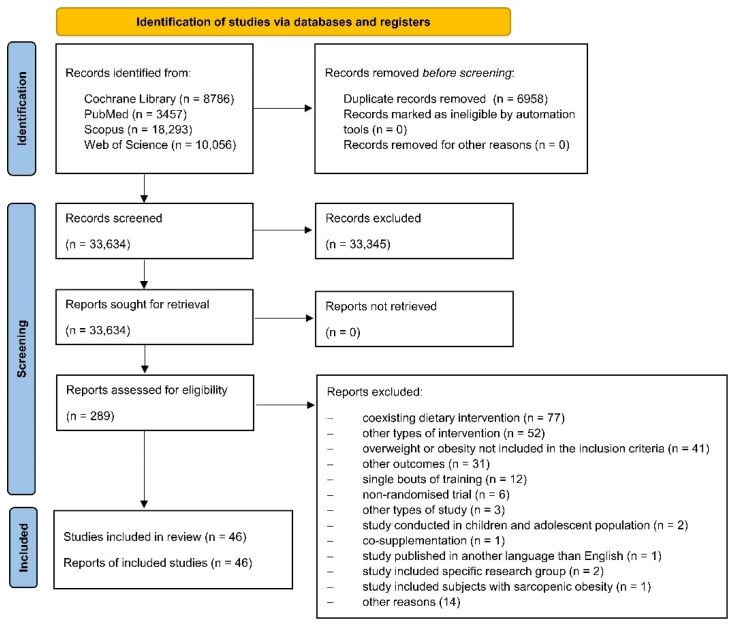
PRISMA 2020 flow diagram.

Each database was screened by two independent researchers (M.J.: PubMed; N.K.: PubMed and Web of Science; M.K.: Cochrane and Scopus; A.M.-B.: Cochrane and Web of Science; A.Ś.: Scopus) and relevant articles were evaluated in three stages (see Figure 1). First, the titles were assessed; subsequently, abstracts were considered, and finally, full texts were assessed. Disagreements were resolved by consensus and all reviewers agreed on the final decision. In case of doubt or missing data, corresponding authors were contacted for more information.

### 2.5. Data Item and Data Collection Process

The following data were extracted from the included papers: first author name, publication year, country, region, the number of subjects included and who completed the intervention, characteristics of the studied population, overweight and/or obesity definition used in the study, age and sex of the study participants, intervention characteristics (study design, type of training, training intensity, frequency, time of intervention and supervision), for each outcome pre- and post-intervention values, changes and *p*-value. The data from the included papers were extracted by two researchers (J.G. & M.J.) and were checked by the third researcher (M.K.). Another investigator (A.M.-B.) converted each parameter to the same units.

### 2.6. Data Analysis

Study participants were categorised using the body mass index (BMI) cut-off values defined by the World Health Organisation (WHO) as overweight (25–29.9 kg/m^2^) or obese (≥30 kg/m^2^) [27]. As the review included the Asian population, special cut-off values for this group were used (overweight: 23–27.5 kg/m^2^ and obesity: >27.5 kg/m^2^) [28]. Waist circumferences (WC) and waist-to-hip ratio (WHR) were categorised according to cut-off points defined by the International Diabetes Federation (IDF) and the WHO, a WC of European men and women should not exceed 94 cm and 80 cm, respectively, whereas 90 cm and 80 cm for Asian men and women [29]. According to WHO guidelines, WHR ≥ 0.9 for men and ≥0.85 for women was defined as abdominal obesity. Moreover, 25% of FM was used as a criterium for diagnosing obesity in men and 32% of FM for women, which is in line with the American Council on Exercise recommendation [30].

The American Diabetes Association recommendations were used to assess glucose metabolism. Impaired glucose tolerance was defined as plasma glucose concentrations of 120 min in the OGTT ranging from 7.8 to 11.0 mmol/L, while impaired fasting glucose was defined as fasting glucose levels from 5.6 to 6.9 mmol/L, normal glucose tolerance was defined as glucose levels at 120 min in the OGTT < 7.8 mmol/L and normal fasting glucose was defined as fasting glucose levels ranging from 3.9 to 5.5 mmol/L. Diabetes mellitus was diagnosed when fasting glucose levels were ≥7.0 mmol/L or glucose levels at 120 min in the OGTT ≥ 11.1 mmol/L or glycated haemoglobin ≥6.5% [31].

Assessment of fasting insulin levels may be performed in numerous ways, and there are no specific reference values. According to the Adult Treatment Panel (ATP) III-Met, insulin resistance is diagnosed if the homeostatic model assessment of the insulin resistance index reaches ≥1.8 [32]. The normal levels of C-peptide were considered to be in a range from 0.9 to 1.8 ng/mL [33].

According to updates to the ATP III of the National Cholesterol Education Program, LDL-C should be <70 mg/dL for patients with a very high risk of cardiovascular disease and <100 mg/dL for those with a high risk of cardiovascular disease. Preferable concentrations of HDL-C are >40 mg/dL for men and >50 mg/dL for women. The levels of TG should not exceed 150 mg/dL and TC levels should remain <200 mg/dL [34].

Methods of selected unit conversion used in the review are presented in Supplementary Table S2. However, the original data were used to perform the meta-analysis, while the tables show the values after unifying the units for easier data interpretation. Moreover, when logarithmic values are presented, data were transformed back to the raw scale.

### 2.7. Risk of Bias in Individual Studies

Two independent researchers (J.G. & M.J.) assessed the risk of bias using the Cochrane risk of bias tool for randomised trials (RoB 2). The following domains were evaluated: bias due to randomisation, bias due to deviations from intended intervention, bias due to missing data, bias due to outcome measurement, and bias due to selection of reported results [35]. Cochrane handbook for systematic reviews of interventions criteria for low risk, some concerns, and high risk of bias was used [25].

### 2.8. Statistical Analysis

Meta-analysis was performed using the Comprehensive Meta-Analysis 3.0 software (Biostat, Inc., Englewood, NJ, USA) and a *p <* 0.05 was considered statistically significant. If data were presented only in a figure, the GetData Graph Digitizer 2.26.0.20 (S. Fedorov, Russia) software was used to extract the data. Data in the tables are presented as means and standard deviations (SD) or equivalent and data synthesis was undertaken, including a calculation of effect sizes with 95% confidence intervals (CIs). If a standard error or a 95% CI was provided instead of SD, these data were converted to SD according to the instructions presented in the Cochrane guidelines. Similarly, if the studies included two or more groups of the same type of training, the groups were combined into a single group according to the formula provided in the Cochrane guidelines [25]. Additionally, if studies provided the median and range instead of means and SD, the mean was calculated by the method of Hozo et al. [36]. Fixed-effects models were used if no heterogeneity was present, while random-effects models were used for moderate and high heterogeneity. Standardised mean differences (SMDs) for post-intervention (or changes) values were used and forest plots were generated to compare effect sizes across studies. Funnel plots were generated and Begg’s and Egger’s tests were performed to assess publication bias. Heterogeneity between studies was evaluated using Cochran Q statistics with *p <* 0.1 indicating significant heterogeneity. The I^2^ test was used to measure consistency between studies. According to the Cochrane handbook for systematic review I^2^ < 40% suggests a low risk of heterogeneity, 40% to 75% is considered a moderate risk of heterogeneity, and >75% indicates a high risk of heterogeneity [25]. A sensitivity analysis was performed to assess the influence of each study on the overall effect. The sensitivity analysis was also performed by excluding studies with a high risk of bias to determine how the exclusion affects the overall effects. A cumulative meta-analysis was performed to evaluate how the effect changed over time with studies sorted from the oldest to the newest. Subgroup analysis was conducted to compare the effect of studies with short (≤12 weeks) and long (>12 weeks) times of the intervention as well as to assess the effectiveness of combined training with the same and longer duration as endurance and strength training alone. Dupuit et al. [37] included two endurance groups. Therefore, in the subgroup analysis, the group which performed the endurance exercises at the same duration as that in combined group was included.

## 3. Results

### 3.1. Search Results

The search process is presented in Figure 1. A total of 40,592 articles were identified, including 6958 duplicate papers. After the screening of the titles and abstracts, 289 full texts were retrieved, with 46 papers finally included in this study [13,14,15,16,20,21,22,38,39,40,41,42,43,44,45,46,47,48,49,50,51,52,53,54,55,56,57,58,59,60,61,62,63,64,65,66,67,68,69,70,71,72,73,74,75], of which the following papers related to the same study conducted on the same population and the same intervention: (1) AbouAssi et al. [20], Bateman et al. [13], Huffman et al. [59], Slentz et al. [15]; (2) Banitalebi et al. [39,40,75]; (3) Stensvold et al. [22,67]. AbouAssi et al. [20], Bateman et al. [13], and Slentz et al. [15] reported the results for the same outcomes. Therefore, only one paper was included in the meta-analysis [20]. Moreover, if several studies reported results from the same project and outcomes but for different time points [13,41,42,59], only those with longer duration were included in the meta-analysis [13,41].

### 3.2. Characteristics of Included Studies

Table 1 presents the characteristics of the included study. All included papers were designed as parallel randomised trials and were published between 2003 [73,74] and 2021 [38,39]. Twenty studies were conducted in Asia [14,16,39,40,41,42,43,45,46,51,52,53,54,55,56,57,58,61,71,72,75], 11 studies were performed in Europe [22,37,38,44,47,60,63,64,65,67,70], seven in North America [13,15,20,49,59,73,74], four in South America [21,48,50,69], three in Australia [62,66,68] and one in Africa [54].

### 3.3. Characteristics of Study Participants

Characteristics of the study population are also shown in Table 1. In total, 2718 adults were included and the most common comorbidities were type 2 diabetes mellitus or impaired glucose tolerance [39,40,45,51,63,65,68,69,70,74], metabolic syndrome [22,41,42,46,49,67] and dyslipidaemia [13,15,20,59]. However, one study included subjects with multiple cardiometabolic syndromes or cardiovascular disease risk factors [49], insulin resistance [50] and non-alcoholic fatty liver disease [53]. Most subjects were middle-aged [13,15,16,20,22,38,39,40,41,43,44,45,46,47,50,51,52,53,54,55,57,59,62,63,65,66,67,68,69,71,73,75] or older [21,37,49,60,64,70,74], while only a few studies included young subjects [14,58,61,72]. One study did not provide information about the age and sex of the study participants [56]. Most studies were performed in a mixed population [13,15,20,22,44,49,57,59,63,66,67,68,69,70] and 15 only included women [21,37,38,39,40,41,42,47,49,50,51,53,54,59,71] and 14 only recruited men [14,16,43,45,46,53,58,60,62,64,65,71,72,73].

### 3.4. Characteristics of Training Intervention

Table 2 presents the characteristics of the exercise intervention. Five articles included an endurance and strength group [43,50,61,68,73], eight studies assess endurance, strength and control intervention [14,16,44,45,49,57,65,71], four papers evaluated the effect of endurance and combined training programmes [38,47,48,54], 11 articles divided the participants into endurance, combined and control groups [21,39,40,52,54,56,60,70,72,74,75], four articles evaluated the effect of endurance, strength and combined training [13,15,20,58] and 11 studies compared endurance, strength, combined and control intervention [22,41,42,46,51,55,62,63,66,67,69]. Moreover, one study included two different types of endurance training and compared them with endurance-strength training [37], one paper included two different types of endurance training and one strength training [53], one study compared two types of combined training with endurance training [64], and one study compared the effect of three types of endurance training, strength training, combined training and control intervention [59]. The duration of intervention ranged from four weeks [14] to 34 weeks [15,20,52]. The length of a single training session lasted from 13 [53] to 90 min [72], while the frequency of intervention varied between one [70] to five times per week [66]. The training intervention was supervised in 39 studies [14,15,16,20,22,37,38,39,40,41,42,43,44,45,46,47,48,49,50,52,53,54,55,59,60,61,62,63,64,65,67,68,69,70,71,73,74,75], not supervised in one study [66] and six studies did not provide information about supervision [21,51,56,57,58,72].

### 3.5. The Effect of Training Intervention on Glucose and Insulin Metabolism

The effect of training programmes on glucose and insulin parameters is presented in Table 3 and Table 4.

#### 3.5.1. The Effect of Training Intervention on Fasting Glucose Levels

In total, 30 studies evaluated the effect of the training intervention on fasting glucose levels [15,16,20,22,37,38,39,40,41,42,43,44,45,46,48,49,50,51,53,54,58,63,65,66,68,69,71,72,73,75]: in 22 studies endurance training was compared with strength training [15,16,20,22,41,42,43,44,45,46,49,50,51,53,58,63,65,66,68,69,71,73], in 19 papers endurance training and combined training were evaluated [15,20,22,37,38,39,40,41,42,46,48,51,54,58,63,66,69,72,75] and 11 studies assessed the effect of strength and combined programmes [13,20,22,41,42,46,51,58,63,66,69].

**Figure 2:**
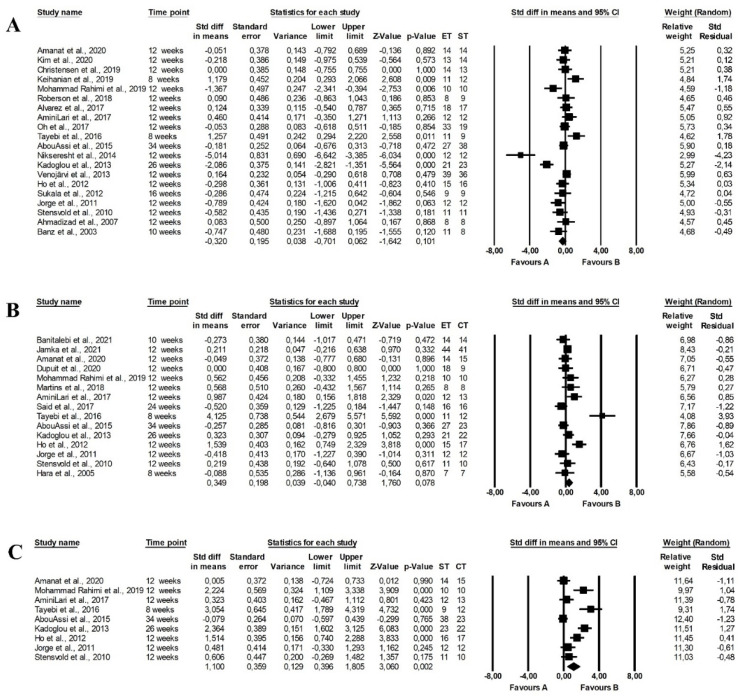
Forest plots of the effect of training programmes on glucose levels: (**A**) endurance (favours A) vs. strength (favours B) training (random model) [16,20,41,43,44,45,46,49,50,51,53,58,63,65,66,67,71,73]; (**B**) endurance (favours A) vs. endurance-strength (favours B) training (random model) [20,22,37,38,39,41,46,48,51,54,58,63,66,69,72]; (**C**) strength (favours A) vs. endurance-strength (favours B) training (random model) [20,22,41,46,51,58,63,66,69]. CI—confidence interval; CT—combined training; ET—endurance training; ST—strength training; Std—standard; Std diff—standard differences.

The meta-analysis reported that endurance and strength training did not differ in the effect on fasting glucose levels (random-effects model, SMD: −0.302, 95% CI: −0.701, 0.062, *p =* 0.101, Figure 2A) with high heterogeneity among the studies (Q-value = 93.888, *p <* 0.001, I^2^ = 79.763%). Similarly, there were no differences between the effect of endurance and combined training programmes on fasting glucose levels (random-effects model, SMD: 0.349, 95% CI: −0.040, 0.738, *p =* 0.078, Figure 2B) and there was moderate heterogeneity among included papers (Q-value = 55.413, *p <* 0.001, I^2^ = 74.735%). However, combined training was more effective in reducing fasting glucose levels than strength training (random-effects model, SMD: 1.100, 95% CI: 0.396, 1.805, *p =* 0.002, Figure 2C) but heterogeneity among included studies was also high (Q-value = 55.743, *p <* 0.001, I^2^ = 85.648%). Funnel plots of standard error by standard differences in means of fasting glucose levels are presented in the Supplementary Materials (see Figure S1).

#### 3.5.2. The Effect of Training Intervention on Fasting Insulin Levels

The effect of training programmes on fasting insulin levels was evaluated in 24 studies [16,20,37,38,39,40,41,42,44,45,46,48,50,51,58,63,65,66,67,68,71,72,73,75], among them 17 papers compared endurance and strength training [16,20,41,42,44,45,46,50,51,58,63,65,66,67,68,71,73], 15 articles assessed endurance and mixed training [20,37,38,39,40,41,42,46,48,51,58,63,66,72,75] and eight studies measured the impact of strength and combined training [20,41,42,46,51,58,63,66].

**Figure 3:**
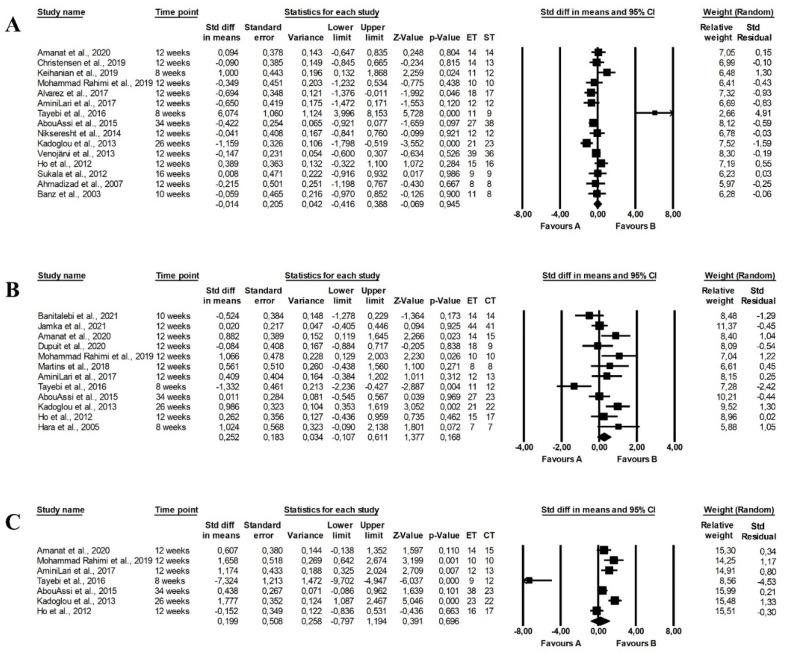
Forest plots of the effect of training programmes on insulin levels: (**A**) endurance (favours A) vs. strength (favours B) training (random model) [16,20,41,44,45,46,50,51,58,63,65,66,68,71,73]; (**B**) endurance (favours A) vs. endurance-strength (favours B) training (random model) [20,38,39,41,42,46,48,51,58,63,66,72]; (**C**) strength (favours A) vs. endurance-strength (favours B) training (random model) [20,41,46,51,58,63,66]. CI—confidence interval; CT—combined training; ET—endurance training; ST—strength training; Std—standard; Std diff—standard differences.

There were no differences between the effect of endurance and strength training (random-effects model, SMD: −0.014, 95% CI: −0.416, 0.388, *p =* 0.945, Figure 3A), endurance and combined training (random-effects model, SMD: 0.252, 95% CI: −0.107, 0.611, *p =* 0.168, Figure 3B) and strength and endurance-strength training (random-effects model, SMD: 0.199, 95% CI: −0.797, 1.194, *p =* 0.696, Figure 3C) on fasting insulin levels in the meta-analysis. The risk of heterogeneity among the studies was high (endurance vs. strength training: Q-value = 58.786, *p <* 0.001, I^2^ = 76.184%, strength vs. combined training: Q-value = 64.421, *p <* 0.001, I^2^ = 90.715%) or moderate (endurance vs. combined training: Q-value = 31.328, *p <* 0.001, I^2^ = 64.887%). Funnel plots of standard error by standard differences in means of fasting insulin levels are provided in Figure S2.

#### 3.5.3. The Effect of Training Intervention on HbA1c Levels

Sixteen studies assessed the effect of training programmes on HbA1c levels [22,37,38,39,40,44,45,46,48,63,65,68,69,70,74,75]. Comparison of the effect of endurance and strength training was evaluated in eight studies [22,44,45,46,63,65,68,69], endurance and endurance-strength training were assessed in 12 articles [22,37,38,39,40,46,48,63,69,70,74,75] and four studies evaluated the strength and mixed training [22,46,66,72].

**Figure 4:**
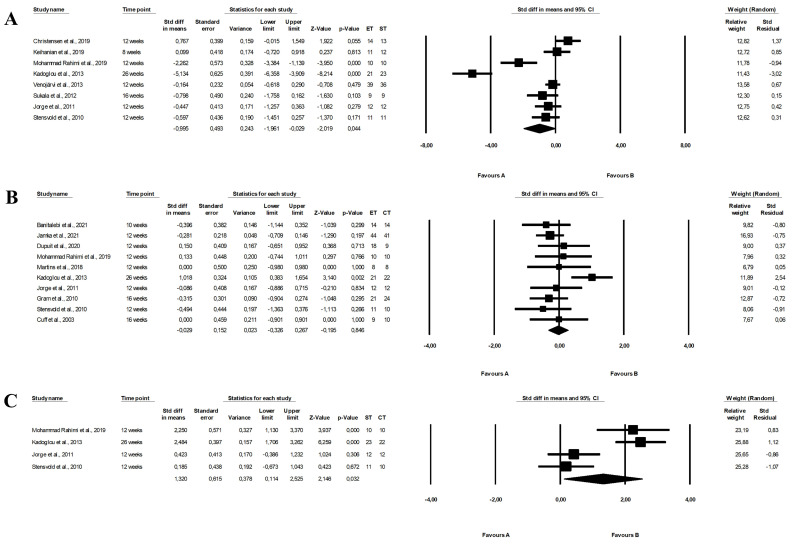
Forest plots of the effect of training programmes on glycated haemoglobin levels: (**A**) endurance (favours A) vs. strength (favours B) training (random model) [22,44,45,46,63,65,68,69]; (**B**) endurance (favours A) vs. endurance-strength (favours B) training (random model) [22,37,38,39,46,48,63,69,70,74]; (**C**) strength (favours A) vs. endurance-strength (favours B) training (random model) [22,46,63,69]. CI—confidence interval; CT—combined training; ET—endurance training; ST—strength training; Std—standard; Std diff—standard differences.

There were significant differences between the effect of endurance and strength training on HbA1c levels, with endurance training being more effective (random-effects model, SMD: −0.995, 95% CI: −1.961, −0.029, *p =* 0.044, Figure 4A). Moreover, combined training had a more favourable effect on HbA1c than strength training (random-effects model, SMD: 1.320, 95% CI: 0.114, 2.525, *p =* 0.032, Figure 4C), but the heterogeneity among studies was high (endurance vs. strength training: Q-value = 79.096, *p <* 0.001, I^2^ = 91.150%; strength vs. combined training: Q-value = 22.648, *p <* 0.001, I^2^ = 86.754). Endurance training did not differ from endurance-strength training in the effect of HbA1c levels (random-effects model, SMD: −0.029, 95% CI: −0.326, 0.267, *p =* 0.846, Figure 4B) with significant heterogeneity among studies (Q-value = 15.015, *p =* 0.090, I^2^ = 40.061%). The effects were plotted against standard error in the funnel plot (see Figure S3).

#### 3.5.4. The Effect of Training Intervention on the HOMA Index

The effect of exercise on HOMA-IR was reported in 26 studies [15,16,20,22,37,38,39,40,41,42,43,45,46,48,50,51,53,55,58,63,65,68,69,71,72,75] including endurance vs. strength training assessed in 19 studies [15,16,20,22,41,42,43,45,46,50,51,53,55,58,63,65,68,69,71], endurance vs. combined programmes reported in 18 articles [15,20,22,37,38,39,40,41,42,46,48,51,55,58,63,69,72,75] and strength vs. endurance-strength exercises evaluated in 11 papers [15,20,22,41,42,46,51,55,58,63,69].

**Figure 5:**
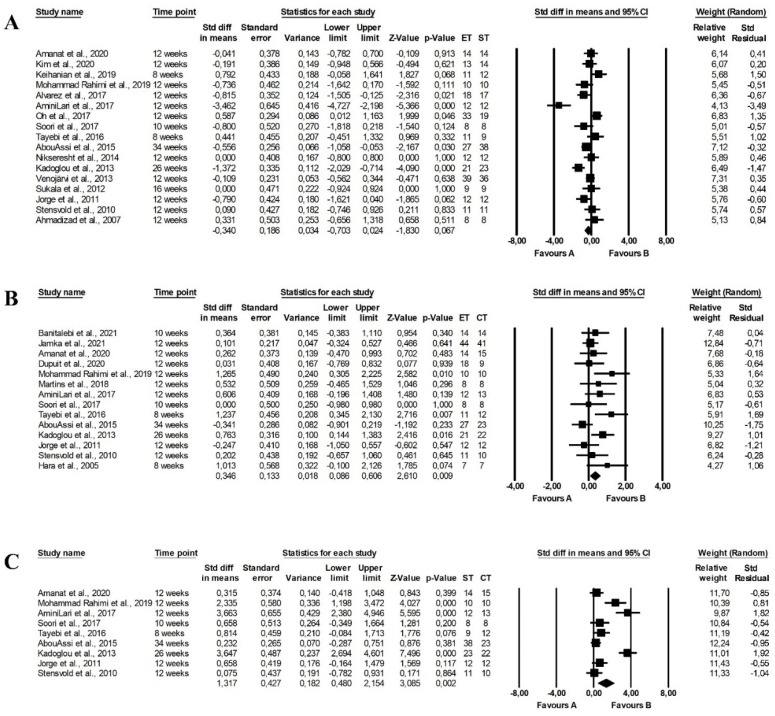
Forest plots of the effect of training programmes on homeostatic model assessment of insulin resistance index: (**A**) endurance (favours A) vs. strength (favours B) training (random model) [16,20,22,41,43,45,46,50,51,53,55,58,63,65,68,69,71]; (**B**) endurance (favours A) vs. endurance-strength (favours B) training (random model); (**C**) strength (favours A) vs. endurance-strength (favours B) training (random model) [20,22,41,46,51,55,58,63,69]. CI—confidence interval; CT—combined training; ET—endurance training; ST—strength training; Std—standard; Std diff—standard differences.

The meta-analysis found no significant differences between the effect of endurance and strength training on the HOMA index (random-effects model, SMD: −0.340, 95% CI: −0.703, 0.024, *p =* 0.067, Figure 5A) and heterogeneity among included studies was moderate (Q-value = 63.198, *p <* 0.001, I^2^ = 74.682%). However, combined training had a more favourable effect on the HOMA index than endurance training (random-effects model, SMD: 0.346, 95% CI: 0.086, 0.606, *p =* 0.009, Figure 5B) and strength training (random-effects model, SMD: 1.317, 95% CI: 0.480, 2.154, *p =* 0.002, Figure 5C) with significant (endurance vs. combined training: Q-value = 21.132, *p =* 0.070, I^2^ = 38.483%) and high (strength vs. combined training: Q-value = 68.722, *p <* 0.001, I^2^ = 88.358%) heterogeneity among the included studies. The publication bias was confirmed by a funnel plot (see Figure S4).

#### 3.5.5. The Effect of Training Intervention on 2 h Glucose Levels

The impact of the intervention on 2 h glucose levels was assessed in two studies. Endurance training was compared with strength training in both studies [62,65] and one study also evaluated the effect of endurance and combined training and resistance and mixed training [62].

**Figure 6:**
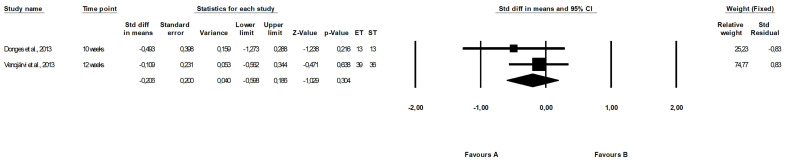
Forest plots of the effect of endurance (favours A) vs. strength (favours B) training programmes on 2 h glucose levels (fixed model) [62,65]. CI—confidence interval; ET—endurance training; ST—strength training; Std—standard; Std diff—standard differences.

The meta-analysis that compared the effect of endurance and strength training reported no differences between the programmes (fixed-effects model, SMD: −0.206, 95% CI: 0.186, −1.029, *p =* 0.304, Figure 6) and low heterogeneity among included studies (Q-value = 0.695, *p =* 0.404, I^2^ = 0.000%).

#### 3.5.6. The Effect of Training Intervention on 2 h Insulin Levels

The effect of training programmes on 2 h insulin concentrations was measured in two studies [62,65]. One study compared endurance with strength training [65] and Donges et al. [62] compared endurance, strength and endurance-strength training.

**Figure 7:**
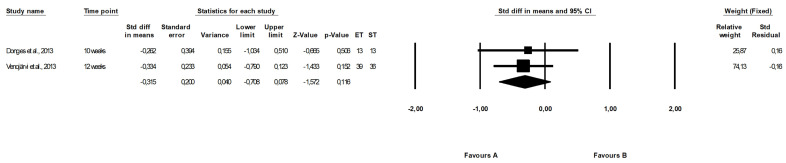
Forest plots of the effect of endurance (favours A) vs. strength (favours B) training programmes on 2 h insulin levels (fixed model) [62,65]. CI—confidence interval; ET—endurance training; ST—strength training; Std—standard; Std diff—standard differences.

No differences between the effect of endurance and strength training were found in the meta-analysis (fixed-effects model, SMD: −0.315, 95% CI: −0.708, 0.078, *p =* 0.116, Figure 7) and the heterogeneity among included papers was low and nonsignificant (Q-value = 0.024, *p =* 0.876, I^2^ = 0.000%).

#### 3.5.7. The Effect of Training Intervention on C-Peptide Levels

C-peptide levels were measured in two studies [22,68], Sukala et al. [68] compared endurance and strength training, while Stensvold et al. [22] assessed the effect of endurance, strength and combined training.

**Figure 8:**
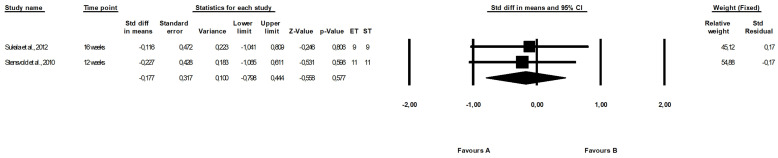
Forest plots of the effect of endurance (favours A) vs. strength (favours B) training programmes on C-peptide levels (fixed model) [22,68]. CI—confidence interval; ET—endurance training; ST—strength training; Std—standard; Std diff—standard differences.

There were no differences between the effect of endurance and strength training (fixed-effects model, SMD: −0.177, 95% CI: −0.798, 0.444, *p =* 0.577, Figure 8) with low heterogeneity among studies (Q-value = 0.030, *p =* 0.861, I^2^ = 0.000%).

### 3.6. The Effect of Training Intervention on Lipid Metabolism

The effect of training programmes on lipid profile is presented in Table 5.

#### 3.6.1. The Effect of Training Intervention on TC levels

The effect of training programmes on TC levels was reported in 28 studies [14,21,22,37,38,41,42,43,44,45,46,47,52,54,55,56,57,60,61,63,64,65,66,68,69,70,72,73]: endurance and strength exercises were compared in 17 papers [14,22,41,42,43,44,45,46,55,57,61,63,65,66,68,69,73], endurance training and endurance-strength training were evaluated in 19 studies [21,22,37,37,41,42,46,47,52,54,55,56,60,63,64,66,69,70,72] and strength training and combined training were assessed in eight articles [22,41,42,46,55,63,66,69].

**Figure 9:**
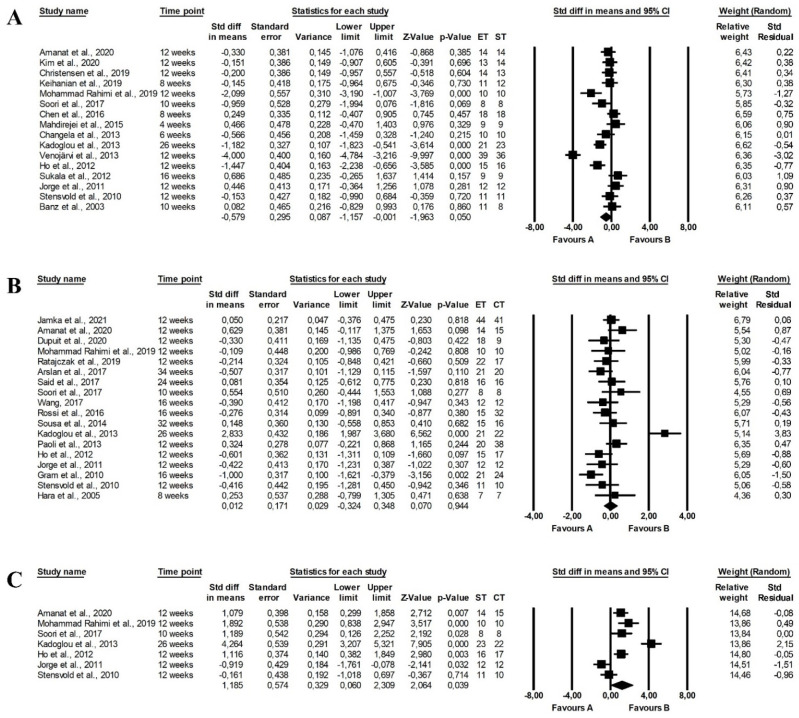
Forest plots of the effect of training programmes on total cholesterol levels: (**A**) endurance (favours A) vs. strength (favours B) training (random model) [14,22,41,43,44,45,46,55,57,61,63,65,66,68,69,73]; (**B**) endurance (favours A) vs. endurance-strength (favours B) training (random model) [21,22,37,38,41,46,47,52,54,55,56,60,63,64,66,69,70,72]; (**C**) strength (favours A) vs. endurance-strength (favours B) training (random model) [22,41,46,55,63,66,69]. CI—confidence interval; CT—combined training; ET—endurance training; ST—strength training; Std—standard; Std diff—standard differences.

The meta-analysis showed that endurance-strength training (random-effects model, SMD: 1.185, 95% CI: 0.060, 2.309, *p =* 0.039, Figure 9C) but not endurance (random-effects model, SMD: −0.579, 95% CI: −1.157, −0.001, *p =* 0.050, Figure 9A) had a more favourable effect on TC levels than strength training with high heterogeneity among studies (Q-value = 66.643, *p <* 0.001, I^2^ = 90.996%, Q-value = 1119.670, *p <* 0.001, I^2^ = 87.465%, respectively). There were no differences between endurance and combined training (random-effects model, SMD: 0.012, 95% CI: −0.324, 0.348, *p =* 0.944, Figure 9B) and heterogeneity among the included papers was high (Q-value = 68.709, *p <* 0.001, I^2^ = 75.256%). The funnel plot was presented in Supplementary Materials (see Figure S5).

#### 3.6.2. The Effect of Training Intervention on LDL-C Levels

The impact of the training programmes on LDL-C concentrations was reported in 24 studies [14,21,37,38,41,42,43,44,45,46,47,52,54,55,56,57,60,63,64,65,66,68,70,73]: endurance vs. strength training was evaluated in 14 papers [14,41,42,43,44,45,46,55,57,63,65,66,68,73], endurance vs. combined training—in 16 studies [21,37,38,41,42,46,47,52,54,55,56,60,63,64,66,70] and strength vs. mixed training—in six studies [41,42,46,55,63,66].

**Figure 10:**
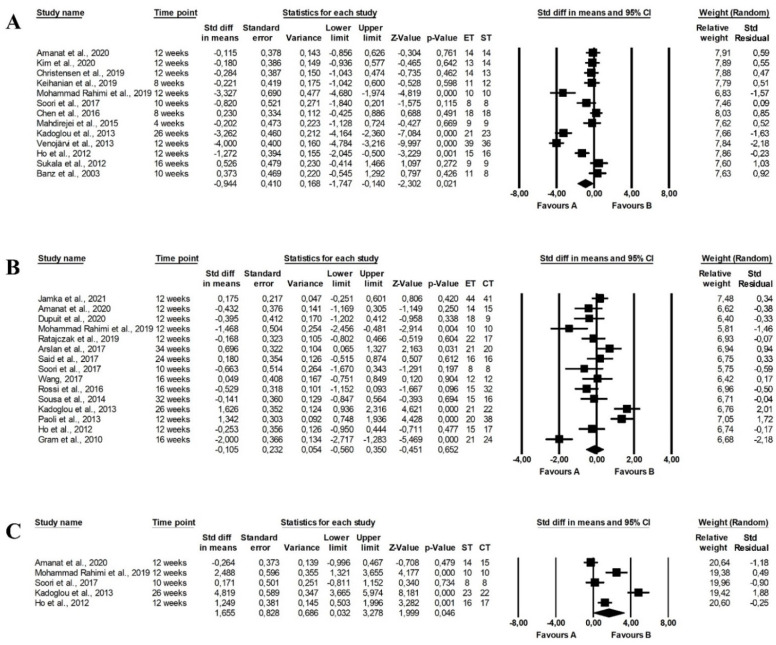
Forest plots of the effect of training programmes on low-density lipoprotein cholesterol levels: (**A**) endurance (favours A) vs. strength (favours B) training (random model) [14,41,43,44,45,46,55,57,63,65,66,68,73]; (**B**) endurance (favours A) vs. endurance-strength (favours B) training (random model) [21,37,38,41,46,47,52,54,55,56,60,63,64,66,70]; (**C**) strength (favours A) vs. endurance-strength (favours B) training (random model) [41,46,55,63,66]. CI—confidence interval; CT—combined training; ET—endurance training; ST—strength training; Std—standard; Std diff—standard differences.

Endurance (random-effects model, SMD: −0.944, 95% CI: −1.747, −0.140, *p =* 0.021, Figure 10A) and endurance-strength training (random-effects model, SMD: 1. 655, 95% CI: 0.032, 3.278, *p =* 0.046, Figure 10C) were more effective in decreasing LDL-C concentrations than strength training, with no differences between endurance and combined training (random-effects model, SMD: −0.105, 95% CI: −0.560, 0.350, *p =* 0.652, Figure 10B) and there was high heterogeneity between studies (endurance vs. strength training: Q-value = 141.921, *p <* 0.001, I^2^ = 91.544%; strength vs. combined training: Q-value = 62.060, *p <* 0.001, I^2^ = 93.544%; endurance vs. combined training: Q-value = 92.565, *p <* 0.001, I^2^ = 84.875%). Funnel plots assessing publication bias are illustrated in Figure S6.

#### 3.6.3. The Effect of Training Intervention on HDL-C Levels

Training programmes that affect HDL-C levels were demonstrated in 31 studies [13,14,21,22,37,38,39,41,42,43,44,45,46,47,49,52,54,55,56,57,60,61,63,64,65,66,68,69,70,72,73], among which endurance was compared with strength programmes in 19 papers [13,14,22,41,42,43,44,45,46,49,55,57,61,63,65,66,68,69,73], endurance and mixed programmes were evaluated in 21 articles [13,21,22,37,38,39,41,46,47,52,54,55,56,60,63,64,66,69,70,72] and strength vs. combined exercises were reported in nine studies [13,22,41,42,46,55,63,66,69].

**Figure 11:**
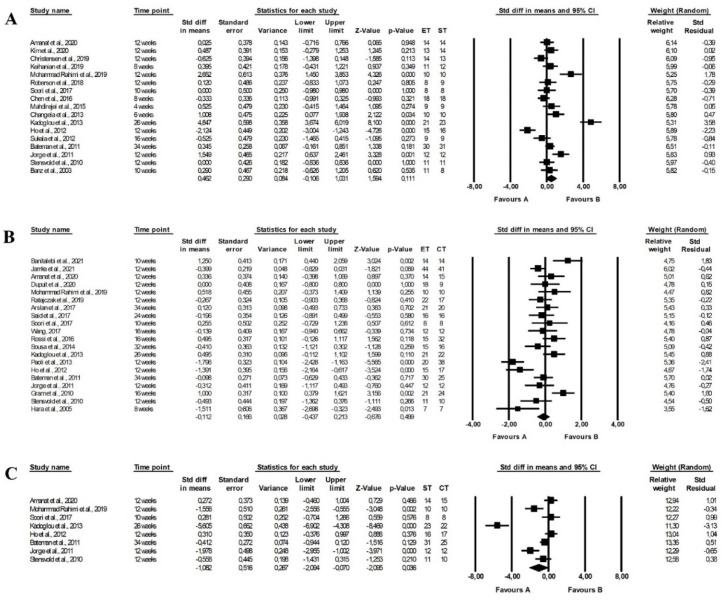
Forest plots of the effect of training programmes on high-density lipoprotein cholesterol levels: (**A**) endurance (favours A) vs. strength (favours B) training (random model) [13,14,22,41,43,44,45,46,49,55,57,61,63,66,68,69,73]; (**B**) endurance (favours A) vs. endurance-strength (favours B) training (random model) [13,21,22,37,38,39,41,46,47,52,54,55,56,60,63,64,66,69,70,72]; (**C**) strength (favours A) vs. endurance-strength (favours B) training (random model) [13,22,41,46,55,63,66,69]. CI—confidence interval; CT—combined training; ET—endurance training; ST—strength training; Std—standard; Std diff—standard differences.

The meta-analysis found no differences between the effect of endurance and strength training (random-effects model, SMD: 0.462, 95% CI: −0.106, 1.031, *p =* 0.111, Figure 11A), endurance and combined training (random-effects model, SMD: −0.112, 95% CI: −0.437, 0.213, *p =* 0. 499, Figure 11B). However, combined training had a more favourable effect on HDL-C levels compared with strength training (random-effects model, SMD: −1.082, 95% CI: −2.094, −0.070, *p =* 0.036, Figure 11C). Heterogeneity between studies was high (endurance vs. strength training: Q-value = 125.025, *p <* 0.001, I^2^ = 87.202%; endurance vs. combined training: Q-value = 81.904, *p <* 0.001, I^2^ = 76.802%; strength vs. combined training: Q-value = 84.119, *p <* 0.001, I^2^ = 91.677%). The results of the funnel plot are shown in Figure S7.

#### 3.6.4. The Effect of Training Intervention on TG Levels

Comparison of endurance, strength and endurance-strength training on TG levels was reported in 28 papers [13,14,22,37,38,39,41,42,43,45,46,47,49,52,53,54,55,56,57,59,60,63,64,65,66,68,69,72], including endurance and strength training results in 18 articles [13,14,22,41,42,43,45,46,49,53,55,57,59,63,65,66,68,69], endurance and endurance-strength training effects in 20 studies [13,22,37,38,39,41,42,46,47,52,54,55,56,59,60,63,64,66,69,72] and the effect of strength and combined training demonstrated in ten papers [13,22,41,42,46,55,59,63,66,69].

**Figure 12:**
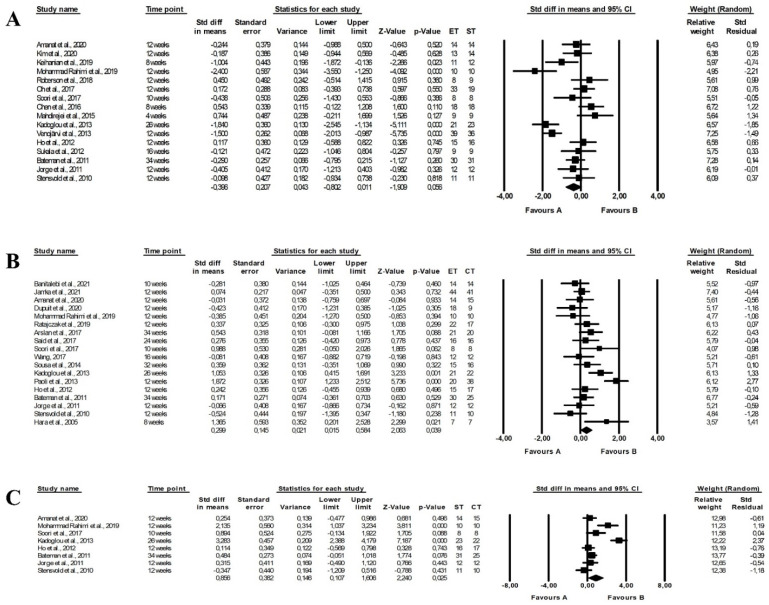
Forest plots of the effect of training programmes on triglycerides levels: (**A**) endurance (favours A) vs. strength (favours B) training (random model) [13,14,22,41,43,45,46,49,53,55,57,63,65,66,68,69]; (**B**) endurance (favours A) vs. endurance-strength (favours B) training (random model) [13,22,37,38,39,41,46,47,52,54,55,56,60,63,64,66,69,72]; (**C**) strength (favours A) vs. endurance-strength (favours B) training (random model) [13,22,41,46,55,63,66,69]. CI—confidence interval; CT—combined training; ET—endurance training; ST—strength training; Std—standard; Std diff—standard differences.

The meta-analysis reported that combined training (random-effects model, SMD: 0.856, 95% CI: 0.107, 1.606, *p =* 0.025, Figure 12C) but not endurance training (random-effects model, SMD: −0.396, 95% CI: −0.802, 0.011, *p =* 0.056, Figure 12A) and had a more favourable effect on TG levels than strength training and combined training was more effective than endurance training (random-effects model, SMD: 0.299, 95% CI: 0.015, 0.584, *p =* 0.039, Figure 12B). The risk of heterogeneity among included studies was high or moderate (endurance vs. strength training: Q-value = 70.873, *p <* 0.001, I^2^ = 78.835%; endurance vs. combined training: Q-value = 49.031, *p <* 0.001, I^2^ = 65.328%; strength vs. combined training: Q-value = 50.068, *p <* 0.001, I^2^ = 86.019%). Funnel plots of standard error by standard differences in means of TG levels are shown in Figure S8.

### 3.7. Risk of Bias

The results of the assessment of the risk of bias are presented in Figures S9 and S10 (see Supplementary Materials). In general, 20 studies were identified as high risk of bias [16,21,37,45,51,52,53,54,55,56,57,58,61,62,64,65,69,71,73,74], 15 papers raised some concerns [14,15,22,42,43,44,46,48,49,50,59,60,63,67,72], and 8 were considered as low risk of bias [13,20,38,39,40,41,47,66,68,70,75].

### 3.8. Sensitivity and Cumulative Analyses

The results of the sensitivity and cumulative analyses are presented in Supplementary Figures S11–S40. The sensitivity analysis results were mostly consistent with those from the primary analysis. However, after excluding studies with a high risk of bias, endurance training was more effective than strength training in reducing glucose levels (random-effects model, SMD: −0.546, 95% CI: −1.090, −0.002, *p =* 0.049, Figure S22A), the HOMA index (random-effects model, SMD: −0.492, 95% CI: −0.844, −0.140, *p =* 0.006, Figure S25A) and TC concentrations (random-effects model, SMD: −0.494, 95% CI: −0.973, −0.015, *p =* 0.043, Figure S26A) and endurance-strength training decreased insulin levels more effectively than endurance (random-effects model, SMD: 0.408, 95% CI: 0.046, 0.770, *p =* 0.027, Figure S23B) and strength training (random-effects model, SMD: 0.828, 95% CI: 0.126, 1.530, *p =* 0.021, Figure S23C). Furthermore, combined training more effectively reduced LDL-C levels than strength training (random-effects model, SMD: 2.033, 95% CI: 0.044, 4.023, *p =* 0.045, Figure S27C) Additionally, differences in HbA1c between endurance and strength training (random-effects model, SMD: −1.565, 95% CI: −3.359, 0.228, *p =* 0.087, Figure S24A) were no longer significant.

### 3.9. Subgroup Analysis

The results of the subgroup analysis are presented in Figures S41–S56. For studies with a short intervention time (≤12 weeks), combined training was more effective than strength training in reducing glucose levels (random-effects model, SMD: 1.085, 95% CI: 0.364, 1.806, *p =* 0.003, Figure S41C), HOMA index (random-effects model, SMD: 1.131, 95% CI: 0.335, 1.927, *p =* 0.005, Figure S44C) and more effective than endurance training in decreasing glucose levels (random-effects model, SMD: 0.359, 95% CI: 0.137, 0.581, *p =* 0.002, Figure S41B), the HOMA index (random-effects model, SMD: 0.368, 95% CI: 0.110, 0.625, *p =* 0.005, Figure S44B). Furthermore, for the long-term interventions (>12 weeks), insulin (random-effects model, SMD: 0.436, 95% CI: 0.018, 0.854, *p =* 0.041, Figure S42B) and TG (random-effects model, SMD: 0.404, 95% CI: 0.100, 0.708, *p =* 0.009, Figure S48B) concentrations were decreased more effectively by endurance-strength training than endurance training. Additionally, combined training more effectively decreased TC (random-effects model, SMD: 4.264, 95% CI: 3.207, 5.321, *p <* 0.001, Figure S45C) and LDL-C (random-effects model, SMD: 4.819, 95% CI: 3.665, 5.974, *p <* 0.001, Figure S46C) levels than strength training but the observation was performed based on the results of one study.

Moreover, the effectiveness of combined training with the same and longer duration as endurance and strength training alone was compared. Interestingly, more efficiency of combined training than strength training was found in studies in which endurance-strength training had a similar duration of volume than strength training alone (glucose (random-effects model, SMD: 1.264, 95% CI: 0.532, 1.997, *p =* 0.001, Figure S49B) and HOMA (random-effects model, SMD: 1.475, 95% CI: 0.517, 2.433, *p =* 0.003, Figure S52B). Moreover, the comparisons of the effect of strength and combined training on HbA1c, TC and LDL-C levels were based on the studies in which both types of activity had the same duration and the analyses also showed that endurance-strength training was more effective than strength training (HbA1c: SMD: 1.320, 95% CI: 0.114, 2.525, *p* = 0.032, Figure 4C; TC: SMD: 1.185, 95% CI: 0.060, 2.309, *p* = 0.039, Figure 9C; LDL-C: SMD: 1. 655, 95% CI: 0.032, 3.278, *p* = 0.046, Figure 10C). Additionally, endurance-strength training was more effective than endurance training alone in decreasing the HOMA index (random-effects model, SMD: 0.415, 95% CI: 0.127, 0.703, *p =* 0.005, Figure S52A) for studies in the similar duration of both programme.

## 4. Discussion

Herein, it is reported that endurance training is more effective in reducing HbA1c and LDL-C levels than strength training, endurance-strength training more effectively decreases glucose, HbA1c, HOMA, TC, LDL-C, HDL-C and TG concentrations than strength training and combined training significantly more reducing HOMA index and TG levels than endurance training. The findings agree with the current physical activity guidelines, which recommend mostly endurance or endurance training combined with strength training for obese subjects at risk of cardiovascular disease [76,77,78].

The recent network meta-analysis of Batrakoulis et al. [23] compared the efficacy of five different exercise modalities (continuous endurance training, interval training, resistance training, combined training and hybrid-type training) on cardiometabolic parameters in overweight and obese subjects and found that hybrid-type training was the most effective for reducing fasting glucose concentrations and increasing HDL-C levels, combined training was the most effective in reducing fasting insulin concentrations, HOMA-IR index and LDL-C levels, and interval training exhibited the highest probability of reducing HbA1c and TG levels. Moreover, subgroup analysis showed that the effects of combined training are more pronounced in males, while hybrid-type training induces more cardiometabolic benefits in females. However, it should be highlighted that the meta-analysis included only studies performed on overweight and obese subjects without comorbidities which may explain the differences in results obtained in this study. Another meta-analysis compared the effect of endurance, strength and combined training in subjects with type 2 diabetes and found that endurance training had a clear but small benefit for TC levels in comparison to strength training, while combined training compared with endurance training was most effective in reducing fasting glucose and HDL-C levels. These findings are mostly in line with these results, but the authors observed no differences between training programmes in the effect of components of the lipid profile of markers of glucose and insulin metabolism [79]. In a meta-analysis, Liang et al. [10] also examined the effects of aerobic, resistance, and combined exercise on metabolic syndrome parameters and cardiovascular risk factors to identify the most effective way of improving metabolic syndrome and preventing cardiovascular disease. The combined exercise was most effective at controlling glucose and TG levels, but there was no statistically significant difference in TC, LDL-C, HDL-C and insulin levels among the exercise groups. Based on the surface under the cumulative ranking curve (SUCRA), combined exercise was also the best for improving insulin and TC levels, resistance exercise was most effective at ameliorating LDL-C levels and aerobic exercise was optimal for improving HDL-C levels.

The mechanism that explains the differences in the effect of endurance, strength and combined training programmes on cardiometabolic parameters has not been clarified. One of the explanations for the more beneficial effect of combined training compared with endurance or strength training is that in several studies, albeit not all, the duration of a single training session was longer compared with endurance or strength training alone [13,15,20,37,48,59,72]. In the Studies of a Targeted Risk Reduction Intervention through Defined Exercise (STRRIDE) study, the participants in the combination groups exercised for approximately double the time of the aerobic and resistance training groups [13,15,20,59]. Martins et al. [48] reported that combined training was two times longer than endurance training. Similarly, in the study performed by Hara et al. [72], combined training consisted of exercises performed in the endurance and strength groups. Therefore, it is not clear if the marked beneficial combination training effects on some markers are due to the greater volume of exercises or a mechanistic synergy of the two exercise modes. However, the results of the subgroup analysis showed that combined training was more effective in decreasing glucose levels and HOMA index than strength training and also than endurance training in decreasing the HOMA index for studies in which combined training had a similar duration as endurance and strength training alone. Nevertheless, it should be highlighted that there was only one study that compared the effect of strength and endurance-strength training on glucose concentrations and HOMA index in which combined training was longer than strength training alone [20].

It was hypothesised that the greater effectiveness of one type of training over another could be related to a higher reduction in body weight and improved body composition. Decreasing visceral FM can particularly affect cardiometabolic parameters and it is well known that abdominal obesity is highly correlated with impaired glycaemic control and lipid profile due to increased visceral fat accumulation [80]. Several studies included in this meta-analysis reported that endurance [16,62] or endurance-strength training [66] was more effective in decreasing body weight compared with strength training. Additionally, Mohammed Rahimi et al. [46] observed that the reduction in %FM and WC in the combined group was significantly greater than in the endurance group, while Tayebi et al. [58] reported that the decrease in %FM in the endurance group was significantly higher than in the strength group, and in endurance-strength training was more pronounced than for both endurance and strength groups. Batrakoulis et al. [23], in the network meta-analysis, found that combined training had the highest probability of being ranked best compared with other exercise types in reducing body weight and the highest likelihood of lowering FM in obese subjects without comorbidities. Liang et al. [10], in another meta-analysis that included studies performed on subjects with a high risk of metabolic syndrome, showed that aerobic, resistance and combined exercise groups achieved significant effects on body fat. However, aerobic exercise was superior to resistance exercise regarding BMI but there was no statistically significant difference in weight and WC among the exercise groups. Notwithstanding, according to the SUCRA results, combined exercise is best for improving weight and WC, while resistance exercise was most effective at ameliorating body fat. Morze et al. [81], in a network meta-analysis performed on subjects with obesity, noted that aerobic training was ranked best for improving body weight, BMI and WC and combined training for improving FM and equally to resistance training for improving free fat mass. By contrast, Yarizadeh et al. [82], in their meta-analysis, compared the effect of aerobic, resistance and combined exercise modalities on subcutaneous abdominal fat and reported that aerobic exercise was shown to produce greater efficacy in decreasing this parameter.

Reduced caloric intake is a crucial factor influencing weight loss and improvement of cardiometabolic parameters. However, in this meta-analysis, only studies in which subjects were instructed not to change their dietary habits during the intervention were included. Indeed, several studies reported no differences in energy values and/or macronutrient distribution in diet between values obtained before and after the intervention period in all study groups [43,49,53]. However, Ho et al. [66] mentioned that when comparing within-group changes, the aerobic and resistance groups had significantly lower daily energy intake at week 12 compared with baseline, but there were no significant differences in total energy intake between groups.

Another mechanism that may explain differences in the effect of endurance, strength and endurance-strength training may be related to differences in energy expenditure between training types. It is important to point out that strength training results in a significantly lower caloric expenditure than a similar amount of time spent in vigorous endurance training. Davidson et al. [83] estimated that the typical strength programme expended 45% of maximal VO_2_, while 75% of maximal VO_2_ was used in the aerobic programme, with 67% more calories likely to be expended in the endurance programme [15]. However, resistance exercise has also been demonstrated to increase basal energy expenditure by increasing muscle volume [84]. Unfortunately, only few studies included in the meta-analysis provided information about energy expenditure by each training programme [13,15,20,50,53,59], and the week energy expenditure was the same in all groups only in one study [50].

Higher adherence to endurance or endurance-strength training than to strength training could partly explain the better effect of the first two training types noted in this meta-analysis. Unfortunately, adherence to the study intervention was reported only in single studies [13,15,20]. AbouAssi et al. [20] showed that participants in the endurance training group were more adherent to the aerobic regimen compared with participants in the combined group. No other group differences in adherence were observed. Bateman et al. [13] found that adherence was slightly lower for each portion of the combined group than for either endurance or strength training. However, the total time accumulated for the combined group remained almost double that of the other exercise groups. Future studies must direct greater attention toward exercise adherence.

It has been demonstrated that more favourable changes in response to training usually occur in subjects with more pronounced disorders at baseline and baseline differences between groups may have an important effect on the obtained results. In the meta-analysis, studies that recruited subjects with and without obesity-related comorbidities were included. Most studies did not have differences at baseline in analysed parameters between groups. Nevertheless, some differences at baseline between groups for study outcomes were reported by Alvarez et al. [50], Oh et al. [53], Hara et al. [72], Venojarvi et al. [65] and Donges et al. [62], which may have some effect on the study results and the meta-analysis findings.

One of the mechanisms by which physical activity can decrease the risk of cardiovascular diseases is the anti-inflammatory effect of exercise [85]. Weight gain may lead to the overproduction of pro-inflammatory cytokines involved in the pathogenesis of cardiometabolic disorders [86]. Therefore, a reduction in low-grade inflammation may accompany improved cardiometabolic markers [87]. Endurance, strength and combined training may affect inflammatory parameters differently. A recent meta-analysis reported that endurance training is more beneficial in reducing C-reactive protein, interleukin 6, and visfatin concentrations in overweight and obese adults than in strength training. Additionally, a combined training programme was significantly more beneficial in lowering tumour necrosis factor α levels compared with a strength training programme [88]. The differences between the effects of particular types of training on inflammatory parameters may be explained by the promotion of other specific cardiovascular and neuromuscular adaptations [89].

The intervention time could also impact the results of the meta-analysis; therefore, a subgroup analysis was performed dividing the studies into two groups with short (≤12 weeks) and long (>12 weeks) intervention periods, showing that for short intervention times, combined training was more effective than strength training in reducing glucose levels and HOMA index and endurance training in decreasing glucose levels and HOMA index. These results can be explained by the difficulty in maintaining high adherence levels in longer intervention studies and could be related to decreased motivation and an increased drop-out rate. However, for the long-term intervention, endurance-strength training more effectively decreased insulin and TG levels than the endurance programme. Additionally, combined training more effectively decreased TC and LDL-C levels than strength training but the observation was performed based on the results of one study. Surprisingly, there were no differences between studies with short and long-term intervention in effect on HbA1c, but HbA1c does not change rapidly and the marker estimates the average glucose levels over the past three months [90].

This is one of the first meta-analyses to compare the effect of endurance, strength and combined training on glucose, insulin and lipid metabolism in overweight and obese subjects (with and without comorbidities) who did not receive dietary intervention or advice. Different criteria were used to define overweight and obesity for different populations and parameters, such as BMI, WC, WHR or %FM, allowing more studies to be included in the analysis. The other strengths of this meta-analysis include the detailed characteristics of the study populations and interventions. Moreover, the effectiveness of combined training with similar and longer duration than endurance and strength training alone was compared.

Nonetheless, this study has several limitations. Firstly, meta-regression and network meta-analysis were not performed. Secondly, there was significant heterogeneity among the included studies despite the strict inclusion and exclusion criteria. In addition, subgroup analysis was not performed regarding sex, age, the health status of participants, intensity and frequency of training. Furthermore, the effect of training programmes on anthropometric parameters and body composition was not assessed as it was comprehensively presented in the recent meta-analysis [81]. Additionally, the use of a strict definition of endurance, strength and combined training in the study protocol was not possible and the effect of each training type on a control group was not compared. The meta-analysis included both studies in which the duration of a single training session was the same in all groups and studies in which combined training had a longer duration than endurance or strength training alone. Therefore, the obtained results may vary in different parameters because of the different duration of a single training session for each type of exercise.

## 5. Conclusions

Endurance and endurance-strength training have a more favourable effect on glucose and insulin homeostasis as well as lipid profile than strength training in overweight and obese adults, with the intervention duration having a significant impact on the obtained results. Moreover, combined training seems to have a more promising effect than endurance training. However, the results from this meta-analysis should be interpreted cautiously due to significant heterogeneity among included studies. Additionally, more studies are needed to assess the impact of training intervention on 2 h glucose and insulin levels and C-peptide.

## Figures and Tables

**Table 1:** Characteristics of included studies.

Author	Year	Country (Region)	Groups	n Included	n Completed	Studied Population	Obesity/Overweight Definition	Age [Years]	Sex [% of Women]
Jamka et al. [38]	2021	Poland (Europe)	ET CT	52 49	44 41	Abdominally obese women	BMI ≥ 30 kg/m^2^ WC > 80 cm %FM ≥ 32%	55 ± 7 ^1^ 55 ± 7 ^1^	100
Banitalebi et al. [39] Banitalebi et al. [40,75]	2021 2019	Iran (Asia)	ET	17	14	Overweight or obese women with T2DM	BMI: 25–48 kg/m^2^	55.36 ± 5.94 ^1^	100
			CT	17	14			54.14 ± 5.43 ^1^	
			CG	18	14			55.71 ± 6.40 ^1^	
Amanat et al. ^a^ [41]	2020	Iran (Asia)	ET	15	14	Overweight or obese women with metabolic syndrome	WC > 88 cm	54.5 ± 6.9 ^1^	100
			ST	15	14				
			CT	15	15				
			CG	15	14				
Dianatinasab et al. ^a^ [42]	2020	Iran (Asia)	ET	15	13	Overweight or obese women with metabolic syndrome	WC > 88 cm	53.47 ± 6.53 ^1^	100
			ST	15	13				
			CT	15	13				
			CG	15	15				
Dupuit et al. [37]	2020	France (Europe)	ET ^2^	10	8	Overweight or obese postmenopausal women	BMI > 25 and ≤40 kg/m^2^	67.1 ± 7.2 ^1^	100
			ET ^3^	10	10			59.9 ± 5.9 ^1^	
			CT	10	9			61.1 ± 5.4 ^1^	
Kim et al. [43]	2020	South Korea (Asia)	ET	19	13	Previously inactive men with obesity	BMI ≥ 25 kg/m^2^	50.15 ± 5.84 ^1^	0
			ST	19	14			51.79 ± 8.22 ^1^	
Christensen et al. [44]	2019	Denmark (Europe)	ET	16	14	Inactive subjects with abdominal obesity	WHR ≥ 0.5 and/or	39 ± 14 ^1^	79
			ST	16	13		WC ≥ 88 cm for women or	38 ± 14 ^1^	62
			CG	18	12		WC ≥ 102 cm for men	47 ± 12 ^1^	83
Keihanian et al. [45]	2019	Iran (Asia)	ET	39	11	Obese men with T2DM	BMI > 30 kg/m^2^	52.4 ± 1.5 ^1^	0
			ST		12			52.4 ± 1.8 ^1^	
			CG		11			53.0 ± 1.1 ^1^	
Mohammad Rahimi et al. [46]	2019	Iran (Asia)	ET	10	10	Sedentary obese men with metabolic syndrome	BMI: 30–40 kg/m^2^	44.8 ± 4.8 ^1^	0
			ST	11	10			46.1 ± 5.1 ^1^	
			CT	12	10			44.9 ± 4.2 ^1^	
			CG	11	10			46.4 ± 5.1 ^1^	
Ratajczak et al. [47]	2019	Poland (Europe)	ET	22	22	Women with simple obesity	BMI ≥ 30 kg/m^2^ and WC > 80 cm and %FM ≥ 33%	51 ± 8 ^1^	100
			CT	22	17			49 ± 10 ^1^	
Martins et al. [48]	2018	Brazil (South America)	ET	14	8	Overweight women with high risk for T2DM, not exercising for at least 6 months	BMI > 24.9 kg/m^2^ and %FM > 40%	64.3 ± 6.7 ^1^	100
			CT	14	8			65.0 ± 6.3 ^1^	
Roberson et al. [49]	2018	USA (North America)	ET	10	8	Older subjects with multiple cardiometabolic syndromes or cardiovascular disease risk factors	WC ≥ 88 cm for women or WC ≥ 102 cm for men	68 ± 3 ^4^	73 (included) 79 (completed)
			ST	10	9			72 ± 3 ^4^	
			CG	10	7			70 ± 3 ^4^	
Alvarez et al. [50]	2017	Chile (South America)	ET	20	18	Sedentary overweight or obese insulin-resistant women	BMI: 25–35 kg/m^2^	38.0 ± 8.0 ^1,5^	100
			ST	20	17			33.0 ± 7.0 ^1,5^	
AminiLari et al. [51]	2017	Iran (Asia)	ET	15	12	Overweight middle-aged women with T2DM	NI	45–60 ^6,7^	100
			ST	15	12				
			CT	15	13				
			CG	15	15				
Arslan et al. [52]	2017	Turkey (Asia)	ET	78	21	Middle-aged overweight premenopausal sedentary women	BMI > 25 kg/m^2^	39.0 ± 3.1 ^1^	100
			CT		20			38.7 ± 2.7 ^1^	
			CG		23			38.9 ± 3.1 ^1^	
Oh et al. [53]	2017	Japan (Asia)	ET ^3^	21	20	Obese sedentary men with non-alcoholic fatty liver disease and no exercise habits	%FM > 25% for men	48.6 ± 1.8 ^4^	0
			ET ^2^	19	13			48.2 ± 2.3 ^4^	
			ST	20	19			51.2 ± 1.9 ^4^	
Said et al. [54]	2017	Tunisia (Africa)	ET	16	NI	Healthy overweight and obese women	BMI: 25–35 kg/m^2^	30.58 ± 3.8 ^1^	100
			CT	16				29.66 ± 4.2 ^1^	
Soori et al. [55]	2017	Iran (Asia)	ET	8	NI	Postmenopausal sedentary obese women	BMI ≥ 30 kg/m^2^	45–60 ^6,7^	100
			ST	8					
			CT	8					
			CG	8					
Wang [56]	2017	China (Asia)	ET	12	NI	Obese undergraduates	BMI ≥ 28 kg/m^2^	NI	NI
			CT	12					
			CG	12					
Chen et al. [57]	2016	Malaysia (Asia)	ET	20	18	Overweight and obese subjects	BMI: 25–40 kg/m^2^	36.8 ± 8.1 ^1^	65
			ST	20	18			34.8 ± 10.6 ^1^	
			CG	20	18			32.4 ± 9.9 ^1^	
Rossi et al. [21]	2016	Brazil (South America)	ET	35	15	Obese postmenopausal Women	BMI > 25 kg/m^2^	60.5 6 ± 7.3 ^1^	100
			CT	35	32			60.3 ± 6.1 ^1^	
			CG	34	18			62.6 ± 5.9 ^1^	
Tayebi et al. [58]	2016	Iran (Asia)	ET	12	11	Non-athlete men with obesity	%FM > 25%	21.48 ± 1.46 ^1,6^	0
			ST	12	9				
			CT	12	12				
AbouAssi et al. ^b^ [20]	2015	USA (North America)	ET	196	27	Sedentary overweight or obese subjects with mild to moderate dyslipidaemia	BMI: 26–35 kg/m^2^	51.4 ± 10 ^1^	52
			ST		38			51.1 ± 11 ^1^	47
			CT		23			46.9 ± 11 ^1^	57
Mahdirejei et al. [14]	2015	Iran (Asia)	ET	9	NI	Obese men with no exercise training history	NI	21/4 ± 15/41 ^1,6^	0
			ST	9					
			CG	8					
Huffman et al. ^b^ [59]	2014	USA (North America)	ET ^8^	15	15	Inactive overweight to mildly obese and dyslipidaemic subjects	BMI: 25–35 kg/m^2^	18–70 ^6,7^	50
			ET ^9^	20	20				
			ET ^10^	17	17				
			ST	20	20				
			CT	20	20				
			CG	20	20				
Nikseresht et al. [16]	2014	Iran (Asia)	ET	12	NI	Sedentary overweight or obese men with no regular exercise and with no history of any medical condition	BMI >25 kg/m^2^	39.6 ± 3.7 ^1^	0
			ST	12				40.4 ± 5.2 ^1^	
			CG	10				38.9 ± 4.1 ^1^	
Sousa et al. [60]	2014	Portugal (Europe)	ET	19	15	Overweight older men	BMI ≥ 25 and <35 kg/m^2^	69.1 ± 5.0 ^1,6^ 65–75 ^6,7^	0
			CT	20	16				
			CG	20	17				
Changela et al. [61]	2013	India (Asia)	ET	10	NI	Young obese sedentary women	BMI > 30 kg/m^2^	22.22 ± 1.98 ^1^	100
			ST	10				22.67 ± 1.50 ^1^	
Donges et al. [62]	2013	Australia (Australia)	ET	13	13	Sedentary overweight middle-aged men	NI	45.4 ± 1.7 ^4^	0
			ST	13	13			51.7 ± 2.1 ^4^	
			CT	13	13			46.2 ± 1.4 ^4^	
			CG	8	8			49.5 ± 2.6 ^4^	
Kadoglou et al. [63]	2013	Greece (Europe)	ET	25	21	Overweight or obese subjects with T2DM	BMI ≥ 25 kg/m^2^	58.3 ± 5.4 ^1^	71
			ST	25	23			56.1 ± 5.3 ^1^	70
			CT	25	22			57.9 ± 6.5 ^1^	77
			CG	25	24			57.9 ± 7.2 ^1^	71
Paoli et al. [64]	2013	Italy (Europe)	ET	21	20	Healthy untrained overweight middle-aged men	BMI > 25 kg/m^2^	61 ± 3.3 ^1,6^	0
			CT ^11^	20	19				
			CT ^12^	19	19				
Venojärvi et al. [65]	2013	Finland (Europe)	ET	48	39	Overweight and obese middle-aged men with impaired glucose tolerance	BMI: 25.1–34.9 kg/m^2^	55 ± 6.2 ^1^	0
			ST	49	36			54 ± 6.1 ^1^	
			CG	47	40			54 ± 7.2 ^1^	
Ho et al. [66]	2012	Australia (Australia)	ET	25	15	Overweight or obese men and women, sedentary or relatively inactive, participating in less than 1 h of moderate-intensity physical activity per week over the last 3 months	BMI >25 kg/m^2^ or WC > 80 cm for women and WC > 90 cm for men	55 ± 1.2 ^13^	80
			ST	26	16			52 ± 1.1 ^13^	81
			CT	25	17			53 ± 1.3 ^13^	82
			CG	21	16			52 ± 1.8 ^13^	94
Stensvold et al. ^c^ [67]	2012	Norway (Europe)	ET	11	11	Inactive subjects with metabolic syndrome	BMI ≥ 30 kg/m^2^ or WC ≥ 80 cm for women or WC ≥ 94 cm for men	49.9 ± 10.1 ^1^	23
			ST	11	10			50.9 ± 7.6 ^1^	
			CG	11	10			47.3 ± 10.2 ^1^	
Sukala et al. [68]	2012	New Zealand (Australia)	ET	13	9	Subjects with T2DM and visceral obesity	WC ≥ 88 cm for women or WC ≥ 102 cm for men	51 ± 4 ^1^ 48 ± 6 ^1^	72
			ST	13	9				
Bateman et al. ^b^ [13]	2011	USA (North America)	ET	73	30	Sedentary overweight dyslipidaemic subjects	BMI: 25–35 kg/m^2^	51.1 ± 9.49 ^1^	47
			ST	66	31			51.8 ± 11.0 ^1^	48
			CT	57	25			45.8 ± 11.8 ^1^	48
Jorge et al. [69]	2011	Brazil (South America)	ET	12	NI ^14^	Overweight or obese subjects with T2DM	BMI: 25–40 kg/m^2^	52.09 ± 8.71 ^1^	58
			ST	12				54.1 ± 8.94 ^1^	58
			CT	12				57.90 ± 8.06 ^1^	67
			CG	12				53.42 ± 9.82 ^1^	67
Slentz et al. ^b^ [15]	2011	USA (North America)	ET	196	48	Sedentary overweight dyslipidaemic subjects	BMI: 26–35 kg/m^2^	49.5 ± 9.8 ^1^	54
			ST		52			49.7 ± 11.4 ^1^	58
			CT		44			46.9 ± 10.0 ^1^	57
Gram et al. [70]	2010	Denmark (Europe)	ET	22	21	Overweight or obese subjects with T2DM	BMI > 25 kg/m^2^	62 ± 10 ^1^	54
			CT	24	24			59 ± 10 ^1^	42
			CG	22	22 ^15^/20 ^16^			61 ± 10 ^1^	41
Stensvold et al. ^c^ [22]	2010	Norway (Europe)	ET	11	NI	Subjects with metabolic syndrome	BMI ≥ 30 kg/m^2^ or WC ≥ 80 cm for women or WC ≥ 94 cm for men	49.9 ± 10.1 ^1^	40
			ST	11				50.9 ± 7.6 ^1^	
			CT	10				52.9 ± 10.4 ^1^	
			CG	11				47.3 ± 10.2 ^1^	
Ahmadizad et al. [71]	2007	Iran (Asia)	ET	8	NI	Sedentary obese healthy men	NI	41.3 ± 5.1 ^1^	0
			ST	8				40.9 ± 3.2 ^1^	
			CG	8				38.6 ± 3.2 ^1^	
Hara et al. [72]	2005	Japan (Asia)	ET	7	7	Young obese men	BMI > 25 kg/m^2^	19.7 ± 1.3 ^1^	0
			CT	7	7			18.4 ± 0.5 ^1^	
			CG	7	7			19.4 ± 1.0 ^1^	
Banz et al. [73]	2003	USA (North America)	ET	14	11	Men with android obesity and at least one risk factor for coronary artery disease	BMI > 27 kg/m^2^ and WHR > 0.95	47 ± 7 ^1^	0
			ST	12	8			48 ± 6 ^1^	
Cuff et al. [74]	2003	Canada (North America)	ET	9	9	Postmenopausal women with T2DM, central obesity and an inactive lifestyle	WC > 90 cm	59.4 ± 1.9 ^4^	100
			CT	10	10			63.4 ± 2.2 ^4^	
			CG	9	9			60.0 ± 2.9 ^4^	

BMI—body mass index; CG—control group; CT—combined training; ET—endurance training; NI—no information; ST—strength training; T2DM—type 2 diabetes mellitus; WC—waist circumference; WHR—waist to hip ratio; %FM—the percentage of fat mass. ^1^ Mean ± standard deviation; ^2^ Moderate-intensity continuous training; ^3^ High-intensity interval training; ^4^ Mean ± standard error; ^5^ 34 ± 6 according to Table 2; ^6^ Data for the total population; ^7^ Range; ^8^ Low-amount moderate-intensity training group; ^9^ Low-amount vigorous-intensity training group; ^10^ High-amount vigorous-intensity training group; ^11^ High-intensity circuit training; ^12^ Low-intensity circuit training; ^13^ Mean ± standard error of means; ^14^ Five subjects dropped out (no information from which groups); ^15^ Data after 16 weeks; ^16^ Data after 52 weeks; ^a–c^ Studies marked with the same letters were conducted in the same population.

**Table 2:** Characteristics of training programmes.

Author	Year	Groups	Characteristic of Groups/Training (Including Volume)	Intensity of Training [%]	Duration of Training [min]	Frequency of Training [Days per Week]	Time of Intervention [Weeks]	Supervision
Jamka et al. [38]	2021	ET	Cycling on ergometer	50–70% of HR max	60	3	12	Yes
		CT	ET: Cycling on ergometer ST: Exercises with a barbell (16 repetitions per set) and a gymnastic ball (30 repetitions per set); between the series 10–15 s pauses were taken	ET: 50–70% of HR max ST: 50–60% of 1 RM				
Banitalebi et al. [39] Banitalebi et al. [40,75]	2021 2019	ET	Sprint interval training on cycle ergometers at a pedalling rate of 20 rpm	60–70% of HR max ^1^	20–50 ^1^	3	10	Yes
		CT	ET: Treadmill or cycle ergometer ST: 1–3 set of 5 exercises of 10–15 repetitions with 10–15 RM ^1^ and 2–3 min rest between sets ^1^; training on weight stack machines: bilateral leg press, lateral pulldown, bench press, bilateral biceps curl, and bilateral triceps pushdown	ET: 60–70% of HR max ^1^ ST:NI				
		CG	Continued their usual medical care and received diabetes recommendations for self-management	N/A	N/A	N/A		
Amanat et al. ^a^ [41] Dianatinasab et al. ^a^ [42]	2020	ET	Running on the treadmill	60–75% of HR max ^1^	30–60 ^1^	3	12 (Amanat et al. [41]) 8 (Dianatinasab et al. [42])	Yes
		ST	2 sets of 10 different exercises of 8–10 repetitions for each exercise and 5–10 min of rest between each set: bench press, seated row, shoulder press, chest press, lateral pulldown, abdominal crunches, leg press, leg extension, triceps pushdown, and seated bicep curls, for upper and lower parts of the body	60–80% of 1 RM ^1^	60	2–3 ^1^		
		CT	Walking on a treadmill, followed by 5 min rest and 1 set of strength training (different exercises similar to the ST group)	ET: 60–75% of HR max ^1^ ST: 60–80% of 1 RM ^1^	60 (including ET: 20)	2–3 ^1^		
		CG	No intervention	N/A	N/A	N/A		
Dupuit et al. [37]	2020	ET ^2^	Cycling program, energy expenditure: 180 ± 22 ^4^	55–60% of peak power output ^1^	40	3	12	Yes
		ET ^3^	Cycling programme (repeated cycles of sprinting/speeding for 8 s followed by slow pedalling (20–30 rpm) for 12 s), energy expenditure: 180 ± 22 ^4^	80–90% of HR peak	20			
		CT	ET: Cycling programme (repeated cycles of sprinting/speeding for 8 s followed by slow pedalling (20–30 rpm) for 12 s) ST: 2 different whole-body training programme each consisting of 1 set of 10 exercises of 8–12 repetitions with 1–1.5 min rest period between exercises: 1. Included leg press, bench press, knee extension, cable row, dumbbell calf raise, elbow flexion, abdominal muscle, triceps exercises with upper pulley, plank, and bum exercises 2. Included knees extension, pullover, leg press, side raise with dumbbells, dumbbell calf raise, triceps exercises with upper pulley, hip thrust, chin rowing, and plank to upright row	ET: 80–90% of HR peak ST: 80% of 1 RM	40 (ET: 20 + ST: 20)			
Kim et al. [43]	2020	ET	Jogging and running outdoors or indoor exercise using stationary cycling	65–85% of HR max	30–60 ^1^	3	12	Yes
		ST	3 sets of 7 exercises of 10–15 repetitions with 1–2 min of rest between each set: crunch, high lat pulldown, seated row, chest press, leg press, leg extension and leg curl	~50% of 1 RM	60			
Christensen et al. [44]	2019	ET	High-intensive interval exercise on an ergometer bicycle	NI	45	3	12	Yes
		ST	3–5 sets of 10 exercises	60–80% of 1 RM ^1^				
		CG	No intervention	N/A	N/A	N/A		
Keihanian et al. [45]	2019	ET	Running	65–75% of HR max (abstract) 75–85% of HR max (method)	30–45	3	10 ^5^	Yes
		ST	3 sets of 10 RM of 7 exercises with 1.5 min rest between sets and 2 min rest between exercises: leg press, bench press, knee extension, seated cable row, knee flexion, military press, and calf rise	NI	60			
		CG	No intervention	N/A	N/A	N/A		
Mohammad Rahimi et al. [46]	2019	ET	4 × 4 min intervals of walking/running on a treadmill, with 3 min exercise between each interval	90% of HR peak (intervals) 70% of HR peak (between intervals)	43	3	12	Yes
		ST	2–3 sets of 7 weight machines exercises of 8–20 repetitions: lateral pulldown, chest press, seated row, triceps pushdown, knee flexion, knee extension, and leg press	40–80% of 1 RM ^1^	45			
		CT	Exercises were similar to the practices of the other two groups	ET: 90% of HR peak (intervals) 70% of HR peak (between intervals) ST: 40–80% of 1 RM ^1^	ET: 43 (performed in the week of 1, 3, 5, 7, 9 and 11 twice a week and ST once a week), ST: 45 (performed in the weeks of 2, 4, 6, 8, 10 and 12 twice a week and ET once a week)			
		CG	The group was advised not to change their physical activity levels throughout the intervention	N/A	N/A	N/A		
Ratajczak et al. [47]	2019	ET	Training on cycle ergometers	60–80% of HR max	60	3	13	Yes
		CT	ET: Similar as described for ET ST: Exercises using a neck barbell and gymnastics ball: upper limb exercises with a neck barbell on Mondays; spine-stabilising exercises, deep muscle-forming exercises, and balance-adjusting exercises with a gymnastic ball on Wednesdays; lower limb exercises with a neck barbell on Fridays; the number of repetitions was systematically increased with the increase in subject’s muscle strength	ET: 60–80% of HR max ST: 50–60% of 1 RM	60 (ET: 25 + ST: 20 + warm up: 5 + cool down: 10)			
Martins et al. [48]	2018	ET	High-intensity interval body weight training; 10 sets of vigorous exercises (30 s of stair climbing and 30 s of body weight squats) interspersed by 60 s of a light walk ^6^	>85% of HR max + recovery at 60–70% of HR max	36	3	12	Yes
		CT	ET: Moderate walking ST: 1–3 sets of 5 resistance exercises of 8–12 repetitions ^1^ with 1.5 min. rest intervals between the sets and exercises (half squat, bench press, leg curl, rowing machine, and unilateral leg extension)	ET: 70% of HR max ST: 70% of 1 RM ^6^	68 (including 30 min. of ET ^6^)			
Roberson et al. [49]	2018	ET	Moderate-intensity treadmill training	55% of HR reserve (±2 bpm)	Prescription time: 35 Actual time: 33 ± 2 ^7^	3	12	Yes
		ST	High-velocity circuit resistance training of 2–3 rotations ^1^ of 11 exercises of 12 repetitions at the specified optimal load (%1RM) in the following order: chest press (50%), leg press (60%), latissimus dorsi pulldown (40%), hip adduction (70%), overhead press (60%), leg curl (60%), seated row (50%), hip abduction (70%), elbow extension (50%), plantar flexion (60%), and elbow flexion (50%)	Börg scale: 6.0 ± 0.2 ^7^	Actual time: 30 ± 2 ^7^			
		CG	No intervention	N/A	N/A	N/A		
Alvarez et al. [50]	2017	ET	High-intensity interval training on cycle ergometers, energy expenditure: 45 kcal/kg/min, ~540 kcal/week	Börg scale: 8–10 70–100% of HR reserve	38	3	12	Yes
		ST	4 exercises per session: biceps curl, shoulder press, and upper row, which were performed using free weights and metal bars, and leg extension using the exercise machine; the programme consisted of an interval of working for 60 s; each interval of work was repeated 3 times and was interspersed by an inactive recovery period of 120 s; energy expenditure: 45 kcal/kg/min, ~540 kcal/week	Börg scale: 8–10 20–50% of 1 RM ^1^	36			
AminiLari et al. [51]	2017	ET	Training on cycle ergometer	50–55% of HR max 5.5–7.1 MET	45 ^1,5^ (25 ET + 20 warm-up)	3	12	NI
		ST	3 sets of 6 weight training exercises of 8 repetitions (leg extension, prone leg curl, abdominal crunch, biceps, triceps, and seated calf)	50–55% of 1 RM 5.5–7.1 MET	NI ^1^ (20 warm-up)			
		CT	Consisted of ET integrated with ST	ET: 50–55% of HR max ST: 50–55% of 1 RM Total: 5.5–7.1 MET	half the execution time of ET/ST			
		CG	No intervention	N/A	N/A	N/A		
Arslan et al. [52]	2017	ET	Major muscle group exercises using basic steps and a minimum of three rhythmic variations of popular dance styles and aerobics	60–70% of HR max	80 (60 ET + 10 warm-up and 10 cool-down)	3	34	Yes
		CT	ET: Similar as described for ET ST: 3 sets of 9 exercises of 15–20 repetitions with 2–3 min between sets; the major muscle groups of the upper and lower limbs were exercised with the use of free weights (dumbbells); exercises used in the programme: biceps curl, triceps extension, sit up, squat, side elevation, shoulder press, side bends, pectoral fly and upright row	ET: 60–70% of HR max ST: 60–70% at 1 RM ^1^	60 (ET: 35 + ST: 25)			
		CG	No intervention	N/A	N/A	N/A		
Oh et al. [53]	2017	ET ^3^	3 sets of 3 min cycling with a 2 min active rest between sets, energy expenditure: 180 kcal	80–85% of VO_2_ max (rest at 50% of VO_2_ max)	13	3	12	Yes
		ET ^2^	Cycling, energy expenditure: 360 kcal	60–65% of VO_2_ max	40			
		ST	Consisted of sit-ups, leg presses, leg extensions, leg curls, chest presses, seated rows, and pulldown, energy expenditure: 180 kcal	To 60% of 1 RM for lower body exercises 30–60% of 1 RM for upper body exercises	NI			
Said et al. [54]	2017	ET	High-impact training involves rhythmic exercises routine in which both feet leave the ground: side by side, step touch, side slot, v-step, grapevine, pivot, cha cha cha, mambo rock-line dance, diamond step, hamstring-curl, heel touch, sit-up and push up, fast walking, turn round, heel side, knee-up, scissors double, hop and jump, jumping jack, side kick, full turn, double kick	70–85% of HR max ^1^	50–60	4	24	Yes
		CT	ET: Low-impact rhythmic exercises ST: 2 sets of muscle-strengthening exercises, with 15 s of rest between exercises and 3 min between sets, conducted on resistance machines: leg extension, leg flexion, bench press, shoulder press, triceps extension, and biceps curl; sit-ups for the abdominal muscles were also performed in all sessions; rhythmic exercises were performed without any jumping (side by side, step touch, side slot, v-step, grapevine, pivot, cha cha cha, mambo rock-line dance, diamond step, hamstring-curl, heel touch, sit-up and push up	ET: 50–65% of HR max ST: 60–80% of 1 RM	60–70 (ET: 30 + ST: 20 + warm-up and cool down: 5–10)			
Soori et al. [55]	2017	ET	Water-based training: swimming or walking in the water	40–60% of HR max ^1^	45	3	10	Yes
		ST	3 sets of 6 dynamic exercises with free weights of 10–12 repetitions: bench press, lateral pulldown, rowing, leg press, hip flexion and extension	40–60% of 1 RM ^1^				
		CT	ET: Swimming ST: 2 sets of 10–12 repetitions of resistance exercises described in the ST group	ET: 40–60% of HR max ST: 40–60% of 1 RM ^1^	44 (ET: 22 + ST: 22)			
		CG	No intervention	N/A	N/A	N/A		
Wang [56]	2017	ET	Aerobics and jogging	60–70% of HR max	60	3	16	NI
		CT	ET: Similar as described for ET ST: 3 groups of 6 movement links repeated 6–8 times: flexion and extension of shoulder joints, elbow joints, hip joints, knee joints, and muscles of the trunk	ET: 60–70% of HR max ST: 60–70% of 1 RM	60 (ET: 40 + ST: 20)	NI		
		CG	No intervention	N/A	N/A	N/A		
Chen et al. [57]	2016	ET	Brisk walking	60–70% of HR max	NI	3	8	NI
		ST	3 sets of 8 exercise stations, 8–15 repetitions for each station of upper and lower body exercises by using dumbbells	NI	45			
		CG	No intervention	N/A	N/A	N/A		
Rossi et al. [21]	2016	ET	Traveling 3 distances (400, 800, and 1,200 m) in the shortest possible	100% of critical velocity	52	3	16	NI
		CT	ET: Similar as described for ET ST: 3–4 sets of 8–15 repetition exercises with 60–90 s between sets: leg press, leg extension, leg curl, bench press, seated row, arm curl, triceps extension, side elevation with dumbbells, and abdominal exercises	ET: 100% of critical velocity ST: 65–80% of maximum ^1^	57 (ET: 30 + ST: 27)			
		CG	No intervention	N/A	N/A	N/A		
Tayebi et al. [58]	2016	ET	Running program	65–85% of HR max ^1^	25–40 ^1^	3	8	NI
		ST	6 sets of 5 exercises of 3–12 repetitions: leg press, knee extension, lat pulldown, biceps curls, dead lift	50–80% of 1 RM	NI			
		CT	ET: Similar as described for ET (one or a half-term ET) ST: 3 sets of 5 listed in the ST group exercises, 4–12 repetitions	ET: 65–85% of HR max ^1^ ST: 50–80% of 1 RM				
AbouAssi et al. ^b^ [20] Bateman et al. ^b^ [13] Slentz et al. ^b^ [15]	2015 2011 2011	ET	Included treadmill, elliptical trainers, cycle ergometers, or any combination of this equivalent to roughly 19.2 km/wk (12 miles/wk), energy expenditure: 14 kcal/kg/week	65–80% of VO_2_ peak ^1^	Prescription time: 44 ± 8 ^4,8^ Actual time: 40 ± 7 ^4,8^	3	34	Yes
		ST	3 sets of 8 exercises of 8–12 repetitions performed on 8 weight-lifting machines designed to target all major muscle groups	70–85% of 1 RM	60			
		CT	The full ET plus the full ST regimens	ET: 65–80% of VO_2_ peak ^1^ ST: 70–85% of 1 RM	ET: Prescription time: 45 ± 92 ^4,8^ Actual time: 35 ± 11 ^4,8^ ST: 60			
Mahdirejei et al. [14]	2015	ET	Run interval training with active relaxation, at 2:1 ratio	65–80% of HR max ^1^	45–60	3	4	Yes
		ST	3 circuits of 8 isotonic exercises of 8–12 repetitions for each movement in a circuit; with 30–60 s intervals between each exercise and with 120–180 s intervals between each circuit: squat to press, arm curl, chest press, knee extension, seated rowing, heel raise, overhead press, and leg curl	60–80% of 1 RM				
		CG	No intervention	N/A	N/A	N/A		
Huffman et al. ^b^ [59]	2014	ET ^9^	Low-amount moderate-intensity exercises, energy expenditure: 1200 kcal/week	40–55% of VO_2_ peak	NI	NI	26	Yes
		ET ^10^	Low-amount vigorous-intensity exercises, energy expenditure: 1200 kcal/week	65–80% of VO_2_ peak		3		
		ET ^11^	High-amount vigorous-intensity exercises, energy expenditure: 2000 kcal/week					
		ST	3 sets, 8–12 repetitions of upper and lower body exercises	NI				
		CT	Linear combination of low-amount vigorous-intensity training and ST	ET: 65–80% of VO_2_ peak ST: NI				
		CG	No intervention	N/A		N/A		
Nikseresht et al. [16]	2014	ET	Running on a treadmill; 4 sets of 4 min with 3 min recovery intervals	80–90% of HR max (recovery intervals at 55–65% of HR max)	25 ^12^	3	12	Yes
		ST	1–4 sets of 12 exercises of 2–20 repetitions with 1–7 min of rest period: knee extension, bench press, incline bench press, seated row, dead lift, pulley crunches, lat pulldowns, calf raise, hamstring curl, press behind neck, upright row, arm curl	40–95% of 1 RM	40–65			
		CG	Continued their normal sedentary life	N/A	N/A	N/A		
Sousa et al. [60]	2014	ET	Trained in a land environment and in an aquatic environment; including walking and/or jogging and/or dancing patterns, and muscular endurance, which included 3 exercises (3 sets, 15–20 repetitions) using only bodyweight and gravity for strengthening the lower and upper limbs in a land environment, and water resistance in an aquatic environment; agility exercises in an informal game format (e. g. relay races, water volleyball and water polo) during the training sessions exclusively in the aquatic environment	Moderate-to-vigorous intensity	60	3	32	Yes
		CT	ET: Similar as described for ET ST: 3 sets of 7 exercises of 8–12 repetitions with 30 s rest periods between sets and 1 min between exercises: bench press, leg press, lateral pulldown, leg extension, military press, leg curl and arm curl and floor exercises for the abdominals and erector spinae muscle groups	65–75% of 1 RM ^1^				
		CG	No intervention	N/A	N/A	N/A		
Changela et al. [61]	2013	ET	Walking, jogging, aerobic dance with music	60–70% of HR max	40	3	6	Yes
		ST	4 sets of 7 different types of exercises of 10 repetitions; training started with 10 lifts with 50% of 10 RM, then 75% of 10 RM and progressed to 100% of 10 RM; seven different types of exercises such as abdominal curl-ups, biceps curls, triceps extension, back extension, leg curls, side leg raises and knee extension were included	NI	NI			
Donges et al. [62]	2013	ET	Cycling with elliptical cross training	75–80% of HR max	40–60 ^1^	3	12	Yes
		ST	Whole-body training program, including chest and shoulder press, seated rows, lat pulldown, leg press, leg curls, lunges, machine squats, and deadlifts; 3–4 sets × 8–10 of each exercise	75–80% of 1 RM ^1^	NI			
		CT	ET: Similar as described for ET ST: 1.5–2 × 8–10 of each exercise described in the ST group	ET: 75–80% of HR max ST: 75–80% of 1 RM ^1^	ET: 20–30 ST:NI			
		CG	No intervention	N/A	N/A	N/A		
Kadoglou et al. [63]	2013	ET	Walking or running on a treadmill, cycling or calisthenics	60–75% of HR max	60	4	26	Yes
		ST	2–3 sets of 8 types of exercises of 8–10 repetitions: seated leg press, knee extension, knee flexion, chest press, lat pulldown, overhead press, biceps curl, and triceps extension	60–80% of 1 RM	60 ^1,5^			
		CT	CT: combined training as in aerobic training group and resistance training group with the following pattern weekly: 1 session of ET programme; 1 session of ST; and 2 sessions combining the types of exercise of both ET and ST in the same session	ET: 60–75% of HR max ST: 60–80% of 1 RM	55 ^1,5^			
		CG	Patients were encouraged to perform self-controlled, leisure-time physical activity (e.g., walking briskly, cycling outdoor)	Low-to-moderate intensity	150/week	N/A		
Paoli et al. [64]	2013	ET	Training on cycloergometer + 4 sets of 20 repetitions of abdominal crunches	50% of HR reserve	50 (ET: 8 + ST: 42)	3	12	Yes
		CT ^13^	ET: training on cycloergometer ST: 2 sets of the following exercises: back: underhand cable pulldowns; chest: pectoral machine; shoulders: lateral shoulder raise; lower limbs: horizontal press; abdomen: 1 set of 20 repetitions abdominal crunches performed with 3 sets of rest-pause; every set consists of 6 RM, 20 min recovery, 2 reps at exhaustion 20 min recovery	ET: 3 min at 50% of HR reserve and 1 min at 75% of HR reserve				
		CT ^14^	ET: training on cycloergometer ST: 2 sets of the following exercises: back: underhand cable pulldowns; chest: pectoral machine; shoulders: lateral shoulder raise; lower limbs: horizontal press, the exercises were performed to reach 15 RM; abdomen: 1 set of 20 repetitions abdominal crunches	ET: 50% of HR reserve				
Venojärvi et al. [65]	2013	ET	Nordic walking consisted of warmup exercises including walking for 5 min and stretching of main muscle groups in addition to walking with poles; after the pole walking, the main muscle groups were stretched for 5 min for cool-down	55–75% of HR reserve ^1^	60	3	12	Yes
		ST	Started with warm-up exercises (cycling or rowing with ergometer for 5 min and stretching of main muscle groups). The main part of programme was performed by using regular resistance equipment, and the training focus was on strength and power exercises of the lower extremities and trunk but also muscles of the upper extremities were trained. Muscle contractions were performed with maximal or high velocity, and external loads were 50–85% from exercise-specific maximal strength, which was determined by the 5RM; At the end of every session, subjects cooled down by cycling or rowing with the ergometer for 5 min and by stretching the main muscle groups	50–85% from exercise-specific maximal strength, which was determined by the 5 RM				
		CG	No intervention	N/A	N/A	N/A		
Ho et al. [66]	2012	ET	Treadmill walking	60% of HR reserve ± 10 beats/min	30	5	12	No
		ST	4 sets of 5 exercises of 8–12 repetitions at 10 RM of leg press, leg curl, leg extension, bench press, rear deltoid row	NI				
		CT	ET: Similar as described for ET ST: 2 sets of 8–12 repetitions at 10 RM of exercises described in the ST group	ET: 60% of HR reserve ± 10 beats/min ST: NI	30 (ET: 15 + ST: 15)			
		CG	No exercise, subjects were requested to continue their normal physical activity and received a placebo dietary supplement only	N/A	N/A	N/A		
Stensvold et al. ^c^ [67] Stensvold et al. ^c^ [22]	2012 2010	ET	Aerobic interval training: as treadmill walking or running (self-selected) consisted of 4 intervals of 4 min at and 3 min active recovery period	Intervals: 90–95% of HR peak Recovery period: 70% of HR peak	43	3	12	Yes
		ST	3 sets of 8–12 repetitions; consisted of two different programs including different muscle groups; the following exercises were performed twice weekly (programme 1): low row, bench press, and hack lift; the alternative programme was performed once each week (programme 2): deltoid exercise (lateral raise exercise), triceps pulldown, biceps curl, and low-row and core exercises (plank exercise)	60–80% of 1 RM ^1^	40–50 ^15^			
		CT	ET twice a week and ST once a week	ET: 90–95% of HR peak ST: 60–80% of 1 RM ^1^	ET: 43 ST: 40–50 ^13^			
		CG	No intervention	N/A	N/A	N/A		
Sukala et al. [68]	2012	ET	Exercises on a cycle ergometer	65–85% of HR reserve ^1^	40–60 ^1^	3	16	Yes
		ST	2–3 sets of 8 exercises of 6–8 repetitions with 1 min rest between sets and exercises; exercises with the use of machine weights targeting all the major muscle groups of the body: seated leg press, knee extension, knee flexion, chest press, lat pulldown, overhead press, biceps curl, and triceps extension	NI				
Jorge et al. [69]	2011	ET	Cycling programme	HR corresponding to the lactate threshold	60	3	12	Yes
		ST	Focused on the large muscle groups and consisted of a 7-exercise circuit as follows: leg press, bench press, lat pulldown, seated rowing, shoulder press, abdominal curls, and knee curls	NI				
		CT	Consisted of ST interchanged with ET performed at the same intensity and half the volume of the ET and ST groups	ET: HR corresponding to the lactate threshold ST:NI				
		CG	Light stretching exercises	N/A	N/A			
Gram et al. [70]	2010	ET	Nordic walking	At least 40% VO_2_ max	45	1–2	16 (36 follow up)	Yes
		CT	Training on ergometer cycles, rowing machines, step machines, and strength training machines (for chest and leg, upper back, and knee extension and flexion)	ET: At least 40% VO_2_ max CT: Börg scale 13–14				
		CG	Written information about exercises and advice to be physically active	N/A	N/A	N/A		
Ahmadizad et al. [71]	2007	ET	Continuous running	75–85% of HR max	20–30 ^1^	3	12	Yes
		ST	4 sets of circuit weight training for 11 stations; the maximum number of repetitions in each station was 12; exercises involving the upper and lower body	50–60% of 1 RM	50–60			
		CG	No intervention	N/A	N/A	N/A		
Hara et al. [72]	2005	ET	Training on treadmills and cycle ergometers	40.8–54.8% of VO_2_ max	30–45	3	8	NI
		CT	ET: Similar as described for ET ST: Exercises: arm curl, triceps extension, and shoulder press for upper-limb training; squat, leg press, leg curl, leg extension, and calf raise for lower-limb training; and bench press, seated butterfly, lat pulldown, trunk curl, back extension, and dead lift for trunk training. Participants selected 2 types each from the upper and lower limb training options, and 3 from trunk training choices, and thus performed 7 exercises in each training session; 3 sets for each exercise consisting of 10 repetition	ET: 40.8–54.8% of VO_2_ max ST: 80% of 1 RM	80–90 (ET: 30+ ST: 50–60)	ET: 3+ R: 2–3	22	
		CG	No intervention	N/A	N/A	N/A	NI	
Banz et al. [73]	2003	ET	Training with ski exercise equipment	60–85% of HR max	40	3	10	Yes
		ST	3 sets of lifts using sub-maximal effort to complete each of 10 lifts/set; 8 different exercises during each workout: military press, leg extension, bench press, leg curl, lateral pulldown, triceps pushdown, biceps curl, and sit-ups	NI	N/A			
Cuff et al. [74]	2003	ET	Programme with using treadmills, stationary bicycles, recumbent steppers, elliptical trainers, and rowing machines	60–75% of HR reserve	75	3	16	Yes
		CT	ET: Similar as described for ET ST: 2 sets of 5 stack weight equipment exercises of 12 repetitions: leg press, leg curl, hip extension, chest press, and latissimus pulldown	ET: 60–75% of HR reserveST: NI				
		CG	No intervention	N/A	N/A	N/A		

CG—control group; CT—combined training; ET—endurance training; HR—heart ratio; MET—metabolic equivalent; N/A—not applicable; NI—no information; RM—repetition maximum; ST—strength training; VO_2_—oxygen uptake. ^1^ Increasing progressively over time; ^2^ Moderate-intensity continuous training; ^3^ High-intensity interval training; ^4^ Mean ± standard deviation; ^5^ Two weeks for the familiarisation with the training and 8 weeks for the main training; ^6^ The goal duration/volume of training; ^7^ Mean ± standard error; ^8^ The total number of min that needed to be obtained was determined by fitness level, as all subjects were prescribed a specific amount of exercise per unit body weight. Higher fit individuals required less time to expend the prescribed number of calories per week; subjects were encouraged not to exceed 60 min/day; ^9^ Low-amount moderate-intensity training group; ^10^ Low-amount vigorous-intensity training group; ^11^ High-amount vigorous-intensity training group; ^12^ Four sets of 4 min training with 3 min recovery; ^13^ High-intensity circuit training; ^14^ Low-intensity circuit training; ^15^ Program 1: 40 min, programme 2: 50 min; ^a–c^ Studies marked with the same letters were conducted in the same population.

**Table 3:** Glucose and insulin metabolism parameters in studied populations.

Author	Year	Group	Glucose [mmol/L]			Insulin [µU/mL]			HbA1c [%]			HOMA		
			Pre	Post	Changes	Pre	Post	Changes	Pre	Post	Changes	Pre	Post	Changes
Jamka et al. [38]	2021	ET CT	5.49 ± 0.72 ^1^ 5.49 ± 0.83 ^1^	5.67 ± 0.89 ^1^ 5.5 ± 0.67 ^1^	0.17 ± 0.55 ^1,2^ 0 ± 0.44 ^1,2^	14.7 ± 7.0 ^1^ 15.7 ± 8.4 ^1^	15.5 ± 10.4 ^1^ 15.3 ± 9.2 ^1^	0.5 ± 10.6 ^1,2^ −1.4 ± 10.7 ^1,2^	5.6 ± 0.4 ^1^ 5.6 ± 0.4 ^1^	5.6 ± 0.4 ^1^ 5.7 ± 0.3 ^1^	0.0 ± 0.5 ^1,2^ 0.1 ± 0.5 ^1,2^	3.66 ± 1.99 ^1^ 3.88 ± 2.19 ^1^	4.04 ± 3.27 ^1^ 3.75 ± 2.35 ^1^	−0.12 ± 2.36 ^1,2^ −0.44 ± 2.44 ^1,2^
Banitalebi et al. [39] Banitalebi et al. [40,75]	2021 2019	ET	11.67 ± 1.83 ^1,3^	7.63 ± 1.83 ^1,3^	−4.04 ^3,5^	10.08 ± 5.43 ^1^	8.18 ± 5.75 ^1^	−1.9 ^5,6^	9.64 ± 1.08 ^1^	7.82 ± 0.93 ^1^	−1.82 (−2.5–−1.14) ^4,7^	1.63 ± 0.83 ^1^	1.15 ± 0.74 ^1^	0.21 ^5^
				8.21 ± 2.29 ^1,3,4^			4.97 ± 1.3 ^1,4^	−5.11 (−7.76–−2.46) ^4,7^						
		CT	11.92 ± 1.54 ^1,3^	9.10 ± 3.97 ^1,3^	−2.82 ^3,5^	10.37 ± 5.35 ^1^	8.83 ± 7.60 ^1^	−1.54 ^5,6^	9.49 ± 0.86 ^1^	8.25 ± 1.22 ^1^	−1.24 (−2.19–−0.29) ^4,7^	1.13 ± 0.28 ^1^	0.95 ± 0.24 ^1^	1.38 ^5^
			11.99 ± 3.50 ^1,3,4^				5.93 ± 2.24 ^1,4^	−4.44 (−7.20–−1.68) ^4,7^						
		CG	11.16 ± 2.60 ^1,3^	10.58 ± 3.32 ^1,3^	−0.58 ^3,5^	9.55 ± 4.05 ^1^	9.16 ± 3.75 ^1^	−0.39 ^5,6^	9.10 ± 0.51 ^1^	9.12 ± 1.41 ^1^	0.02 (−0.67–0.71) ^4,7^	1.39 ± 0.63 ^1^	1.42 ± 0.71 ^1^	1.12 ^5^
			10.95 ± 2.61 ^1,3,4^	11.28 ± 3.37 ^1,3,4^			9.21 ± 2.06 ^1,4^	−0.33 (−2.49–1.83) ^4,7^						
		*p* ^8^	*p =* 0.0001 (group) *p =* 0.03 (time × group) ET: *p =* 0.001 (pre vs. post) ^4^			*p =* 0.02 (group) ET, CT: *p =* 0.001 (pre vs. post) ^4^ 0.036 (between groups) ^4^			*p =* 0.0001 (group) *p =* 0.006 (time × group) ET: *p =* 0.0001 (pre vs. post) CT: 2021: *p =* 0.01, 2019: *p =* 0.002 (pre vs. post)			*p =* 0.007 (group) *p =* 0.02 (time × group)		
Amanat et al. ^a^ [41]	2020	ET ST CT CG	8.77 ± 1.74 ^1,3^ 8.83 ± 1.72 ^1,3^ 9.01 ± 1.51 ^1,3^ 9.08 ± 1.21 ^1,3^	8.51 ± 1.76 ^1,3,9^ 8.65 ± 1.61 ^1,3,9^ 8.64 ± 1.51 ^1,3,9^ 9.13 ± 1.31 ^1,3,9^	−0.26 ± 0.27 ^1,2,3,10,11^ −0.18 ± 0.29 ^1,2,3,10,11^ −0.36 ± 0.27 ^1,2,3,10,11^ 0.05 ± 0.22 ^1,2,3,10,11^	10.62 ± 1.03 ^9^ 10.66 ± 1.50 ^9^ 10.60 ± 1.35 ^9^ 10.34 ± 1.55 ^9^	10.03 ± 0.91 ^9^ 9.91 ± 1.56 ^9^ 9.05 ± 1.27 ^9^ 10.46 ± 1.7 ^9^	−0,58 ± 0.63 ^2,10,11^ −0.74 ± 0.66 ^2,10,11^ −1.55 ± 1.16 ^2,10,11^ 0.12 ± 0.063 ^2,10,11^	NI	NI	NI	4.11 ± 0.74 ^9^ 4.13 ± 0.67 ^9^ 4.24 ± 0.95 ^9^ 4.18 ± 0.85 ^9^	3.69 ± 0.77 ^9^ 3.72 ± 0.68 ^9^ 3.48 ± 0.83 ^9^ 4.20 ± 0.99 ^9^	−0.41 ± 0.27 ^2,10,11^ −0.4 ± 0.53 ^2,10,11^ −0.76 ± 0.46 ^2,10,11^ 0.03 ± 0.29 ^2,10,11^
		*p* ^8^	ET: *p =* 0.003, ST: *p =* 0.037, CT: *p <* 0.001 (pre vs. post) ET vs. CG, CT vs. CG: *p <* 0.05 (post) ET, CT vs. CG: *p <* 0.05 (changes)			ET: *p =* 0.004, ST: *p =* 0.001, CT: *p <* 0.001 (pre vs. post) ST vs. CG, CT vs. CG, CT vs. ET, CT vs. ST: *p <* 0.05 (post) ST vs. CG, CT vs. CG: *p <* 0.05 (changes)						ET: *p <* 0.001, ST: *p =* 0.012, CT: *p <* 0.001 (pre vs. post) ET vs. CG: *p =* 0.022 (post) ST vs. CG: *p=* 0.032 (post) CT vs. CG: *p <* 0.001 (post) ET vs. CG, ST vs. CG, CT vs. CG: *p <* 0.05 (changes)		
Dianatinasab et al. ^a^ [42]	2020	ET ST CT CG	7.16 ± 1.76 ^1,3^ 6.42 ± 0.71 ^1,3^ 7.26 ± 0.53 ^1,3^ 6.62 ± 0.50 ^1,3^	5.84 ± 1.56 ^1^ 6.29 ± 0.72 ^1^ 6.17 ± 0.55 ^1^ 6.80 ± 0.50 ^1^	−1.35 ^3,5,10^ −0.53 ^3,5,10^ −1.08 ^3,5,10^ 0.07 ^3,5,10^	11.57 ± 1.04 ^1^ 10.58 ± 1.55 ^1^ 10.72 ± 1.38 ^1^ 10.15 ± 1.60 ^1^	10.50 ± 1.09 ^1^ 9.32 ± 1.52 ^1^ 8.45 ± 1.62 ^1^ 9.98 ± 1.61 ^1^	NI	NI	NI	NI	3.37 ± 0.49 ^1^ 3.02 ± 0.65 ^1^ 3.46 ± 0.48 ^1^ 2.98 ± 0.35 ^1^	2.72 ± 0.51 ^1^ 2.60 ± 0.64 ^1^ 2.31 ± 0.55 ^1^ 3.01 ± 0.54 ^1^	−0.63 ^5,10^ −0.37 ^5,10^ −1.07 ^5,10^ 0.04 ^5,10^
		*p* ^8^	ET: *p =* 0.011, CT: *p =* 0.022 (pre vs. post) *p =* 0.01 (time × group)			ST: *p =* 0.042, CT: *p =* 0.011 (pre vs. post) *p =* 0.007 (time × group)						ET: *p =* 0.035, ST: *p =* 0.050, CT: *p =* 0.001 (pre vs. post) *p =* 0.001 (time × group)		
Dupuit et al. [37]	2020	ET ^12^ ET ^13^ CT	1.2 ± 0.7 ^1^ 1.2 ± 0.3 ^1^ 1.2 ± 0.6 ^1^	1.2 ± 0.4 ^1^ 1.2 ± 0.3 ^1^ 1.2 ± 0.7 ^1^	NI	11.2 ± 3.0 ^1^ 12.9 ± 14.8 ^1^ 11.5 ± 3.3 ^1^	9.1 ± 3.3 ^1^ 12.5 ± 13.0 ^1^ 11.7 ± 4.4 ^1^	NI	5.6 ± 0.5 ^1^ 6.1 ± 0.9 ^1^ 5.8 ± 0.2 ^1^	5.5 ± 0.4 ^1^ 6.0 ± 0.7 ^1^ 5.7 ± 0.2 ^1^	NI	3.0 ± 1.2 ^1^ 3.9 ± 4.7 ^1^ 2.8 ± 0.8 ^1^	2.2 ± 0.8 ^1^ 3.8 ± 4.4 ^1^ 3.0 ± 1.4 ^1^	NI
Kim et al. [43]	2020	ET ST	5.45 ± 0.76 ^1,3^ 5.52 ± 0.63 ^1,3^	5.40 ± 0.63 ^1,3^ 5.56 ± 0.92 ^1,3^	−0.047 ± 0.46 ^1,3^ 0.059 ± 0.46 ^1,3^	NI	NI	NI	NI	NI	NI	2.24 ± 1.16 ^1^ 2.15 ± 1.15 ^1^	1.88 ± 0.81 ^1^ 2.07 ± 1.14 ^1^	−0.35 ± 0.84 ^1^ −0.08 ± 0.63 ^1^
Christensen et al. [44]	2019	ET ST CG	5.1 ± 0.5 ^1,3^ 4.7 ± 0.4 ^1,3^ 5.1 ± 0.5 ^1,3^	5.1 (4.9–5.3) ^14^ 5.1 (4.8–5.3) ^14^ 5.0 (4.7–5.3) ^14^	−0.2 (−0.4–0.0) ^14^ −0.2 (−0.4–0.1) ^14^ −0.1 (−0.3–0.2) ^14^	NI	12.38 (8.78–15.98) ^3,14^ 12.96 (9.07–16.99) ^3,14^ 16.99 (12.96–21.02) ^3,14^	−1.23 (−5.47–1.87) ^3,14^ −1.15 (−5.18–2.74) ^3,14^ 2.74 (−1.30–6.77) ^3,14^	5.8 ± 0.4 ^1^ 5.7 ± 0.5 ^1^ 6.2 ± 0.6 ^1^	4.8 (4.6–4.8) ^3,14^ 4.6 (4.5–4.8) ^3,14^ 4.6 (4.5–4.8) ^3,14^	4 (1–6) ^14^ 1 (−1–4) ^14^ 3 (0–6) ^14^	NI	NI	NI
Keihanian et al. [45]	2019	ET ST CG	9.59 ± 0.49 ^1,3^ 9.68 ± 0.41 ^1,3^ 9.35 ± 0.39 ^1,3^	7.59 ± 0.27 ^1,3^ 7.18 ± 0.42 ^1,3^ 8.91 ± 0.63 ^1,3^	−1.99 ^3,5^ −2.50 ^3,5^ −0.44 ^3,5^	8.7 ± 0.2 ^1^ 8.6 ± 0.2 ^1^ 8.8 ± 0.2 ^1^	8.3 ± 0.2 ^1^ 8.1 ± 0.2 ^1^ 8.7 ± 0.2 ^1^	−0.39 ^3,5^ −0.50 ^3,5^ −0.10 ^3,5^	7.5 ± 1.2 ^1^ 7.8 ± 1.1 ^1^ 7.2 ± 1.6 ^1^	7 ± 1.2 ^1^ 6.9 ± 0.8 ^1^ 7.2 ± 1.7 ^1^	−6.6 ^5^ −11.5 ^5^ 0 ^5^	3.7 ± 0.4 ^1^ 3.6 ± 0.3 ^1^ 3.5 ± 0.6 ^1^	2.8 ± 0.3 ^1^ 2.6 ± 0.2 ^1^ 3.3 ± 0.6 ^1^	−0.9 ^3,5^ −1.0 ^3,5^ −0.2 ^3,5^
		*p* ^8^	ET, ST: *p <* 0.05 (pre vs. post) ET vs. CG, ST vs. CG, ET vs. ST: *p <* 0.05 (post)			ET, ST: *p <* 0.05 (pre vs. post) ET vs. CG, ST vs. CG: *p <* 0.05 (post)			ET, ST: *p <* 0.05 (pre vs. post) ET vs. CG, ST vs. CG, ET vs. ST: *p <* 0.05 (post)			ET, ST: *p <* 0.05 (pre vs. post) ET vs. CG, ST vs. CG: *p <* 0.05 (post)		
Mohammad Rahimi et al. [46]	2019	ET ST CT CG	5.59 ± 0.27 ^1,3^ 5.62 ± 0.19 ^1,3^ 5.54 ± 0.32 ^1,3^ 5.57 ± 0.21 ^1,3^	4.78 ± 0.54 ^1,3^ 5.43 ± 0.39 ^1,3^ 4.51 ± 0.41 ^1,3^ 5.53 ± 0.3 ^1,3^	NI	23.7 ± 2.7 ^1^ 21.7 ± 3.5 ^1^ 21.8 ± 3.2 ^1^ 22.3 ± 4.2 ^1^	13.9 ± 4.1 ^1^ 15.2 ± 3.3 ^1^ 10.2 ± 2.7 ^1^ 23.1 ± 2.7 ^1^	NI	6.4 ± 0.8 ^1^ 6.5 ± 0.7 ^1^ 6.5 ± 0.7 ^1^ 6.4 ± 0.8 ^1^	4.3 ± 0.7 ^1^ 6.0 ± 0.8 ^1^ 4.2 ± 0.8 ^1^ 6.2 ± 0.9 ^1^	NI	5.9 ± 0.7 ^1^ 5.4 ± 0.9 ^1^ 5.4 ± 0.9 ^1^ 5.5 ± 1.1 ^1^	3.0 ± 1.0 ^1^ 3.7 ± 0.9 ^1^ 2.0 ± 0.5 ^1^ 5.7 ± 0.8 ^1^	NI
		*p* ^8^	ET, CT: *p <* 0.001 (pre vs. post) ET vs. CG, CT vs. CG: *p <* 0.05 (post)			ET, ST, CT: *p <* 0.001 (pre vs. post) ET vs. CG, ST vs. CG, CT vs. CG: *p <* 0.05 (post)			ET, CT: *p <* 0.001 (pre vs. post) ET vs. CG, CT vs. CG, ET vs. ST, CT vs. ST: *p <* 0.05 (post)			ET, ST, CT: *p <* 0.001 (pre vs. post) ET vs. CG, ST vs. CG, CT vs. CG: *p <* 0.05 (post)		
Martins et al. [48]	2018	ET CT	6.09 ± 1.28 ^1,3^ 5.28 ± 0.83 ^1,3^	5.64 ± 0.78 ^1,3^ 5.14 ± 0.95 ^1,3^	−0.46 ^3,5^ −0.14 ^3,5^	13.6 ± 6.11 ^3,15^ 9.9 ± 5.8 ^1,3,15^	11.1 ± 4.2 ^1,3,15^ 8.6 ± 4.7 ^1,3,15^	−18.3 ^5,16^ −13.1 ^5,16^	6.2 ± 0.5 ^1,3^ 6.1 ± 0.4 ^1,3^	5.9 ± 0.3 ^1,3^ 5.9 ± 0.2 ^1,3^	−4.8 ^5^ −3.3 ^5^	3.8 ± 2.2 ^1,3^ 2.4 ± 1.7 ^1,3^	2.8 ± 1.1 ^1,3^ 2.1 ± 1.5 ^1,3^	−1.0 ^3,5^ −0.3 ^3,5^
		*p* ^8^	ET, CT: *p <* 0.05 (pre vs. post) *p =* 0.045 (time × group)			ET, CT: *p <* 0.05 (pre vs. post) *p =* 0.022 (time × group)			ET, CT: *p <* 0.05 (pre vs. post) *p =* 0.021 (time × group)			ET, CT: *p <* 0.05 (pre vs. post) *p =* 0.025 (time × group)		
Roberson et al. [49]	2018	ET ST CG	5.44 ± 0.83 ^3,17^ 5.33 ± 0.41 ^3,17^ 4.94 ± 0.43 ^3,17^	4.94 ± 0.55 ^3,17^ 4.83 ± 0.27 ^3,17^ 5.12 ± 0.37 ^3,17^	−0.5 ± 0.22 ^3,17^	NI	NI	NI	NI	NI	NI	NI	NI	NI
		*p* ^8^	ST: *p* ≤ 0.05 (pre vs. post)											
Alvarez et al. [50]	2017	ET ST	5.8 ± 0.3 ^1,3^ 5.7 ± 0.4 ^1,3^	5.4 ± 0.5 ^1,3^ 5.4 ± 0.4 ^1,3^	−0.39 ± 0.39 ^1,3^ −0.33 ± 0.39 ^1,3^	16.5 ± 4.6 ^1,18^ 18.1 ± 4.9 ^1,18^	8.7 ± 3.3 ^1,18^ 11.2 ± 3.9 ^1,18^	−7.8 ± 1.3 ^1,18^ −6.9 ± 4.4 ^1,18^	NI	NI	NI	4.2 ± 1.1 ^1^ 4.4 ± 1.0 ^1^	2.1 ± 0.7 ^1^ 2.8 ± 1.0 ^1^	−2.1 ± 0.4 ^1^ −1.6 ± 1.0 ^1^
		*p* ^8^	*p <* 0.001 (pre vs. post)			*p =* 0.003 (pre) *p <* 0.0001 (pre vs. post)						*p =* 0.005 (pre) *p <* 0.0001 (pre vs. post) ET vs. ST: *p =* 0.026 (post)		
AminiLari et al. [51]	2017	ET ST CT CG	9.57 ± 1.01 ^1,3^ 9.43 ± 1.26 ^1,3^ 9.44 ± 1.18 ^1,3^ 10.10 ± 0.68 ^1,3^	7.76 ± 1.21 ^1,3^ 7.18 ± 1.36 ^1,3^ 6.86 ± 0.52 ^1,3^ 9.26 ± 0.46 ^1,3^	−2.35 ^3,5^ −2.09 ^3,5^ −2.49 ^3,5^ −0.81 ^3,5^	13.25 ± 3.92 ^1^ 14.43 ± 3.09 ^1^ 12.16 ± 3.62 ^1^ 12.70 ± 3.39 ^1^	14.90 ± 5.51 ^1^ 18.53 ± 5.65 ^1^ 13.01 ± 3.62 ^1^ 14.30 ± 3.36 ^1^	1.73 ^3,5^ 4.23 ^3,5^ 1.22 ^3,5^ 1.83 ^3,5^	NI	NI	NI	5.50 ± 1.2 ^1^ 5.94 ± 0.98 ^1^ 4.97 ± 0.98 ^1^ 5.66 ± 1.37 ^1^	4.53 ± 0.88 ^1^ 18.53 ± 5.65 ^1,19^ 3.92 ± 1.11 ^1^ 5.90 ± 1.56 ^1^	−0.9 ^3,5^ −0.3 ^3,5^ −1.0 ^3,5^ 0.3 ^3,5^
		*p* ^8^	ET, CT: *p =* 0.001 (pre vs. post) ST, CG: *p =* 0.005 (pre vs. post) ET vs. CG, ST vs. CG, CT vs. CG: *p <* 0.05 (post) CG: *p =* 0.02 (pre vs. post) *p =* 0.001 (group)			ST: *p =* 0.02 (pre vs. post)						ET: *p =* 0.004 (pre vs. post) ET vs. ST, ET vs. CG, CT vs. ST, CT vs. CG *p <* 0.05 (post) CT: *p =* 0.005 (pre vs. post) CG: *p =* 0.002 (group)		
Oh et al. [53]	2017	ET ^13^ ET ^12^ ST	2.009 ± 0.008 ^2,17,20^ 1.947 ± 0.021 ^2,17,20^ 1.991 ± 0.010 ^2,17,20^	2.003 ± 0.013 ^2,17,20^ 1.962 ± 0.006 ^2,17,20^ 1.990 ± 0.015 ^2,17,20^	−0.006 ^2,5,17,20^ 0.015 ^2,5,17,20^ −0.001 ^2,5,17,20^	NI	NI	NI	NI	NI	NI	3.45 ± 0.50 ^17^ 2.24 ± 0.37 ^17^ 2.00 ± 0.24 ^17^	3.25 ± 0.48 ^17^ 2.18 ± 0.29 ^17^ 1.88 ± 0.25 ^20^	−0.20 ^5^ −0.06 ^5^ −0.12 ^5^
		*p* ^8^	ET ^13^ vs. ET ^12^ vs. ST: *p <* 0.01 (pre)									ET ^13^ vs. ET12 vs. ST: *p <* 0.05 (pre)		
Said et al. [54]	2017	ET CT	5.69 ± 0.26 ^1,3^ 5.90 ± 0.57 ^1,3^	5.62 ± 0.25 ^1,3^ 5.85 ± 0.57 ^1,3^	−0.091 ± 0.046 ^1,3^ −0.060 ± 0.041 ^1,3^	NI	NI	NI	NI	NI	NI	NI	NI	NI
Soori et al. [55]	2017	ET ST CT CG	NI	NI	NI	NI	NI	NI	NI	NI	NI	2.4 ± 0.6 ^1,3,10^ 1.8 ± 0.7 ^1,3,10^ 2.1 ± 0.9 ^1,3,10^ 1.96 ± 0.85 ^1,3,10^	1.55 ± 0.55 ^1,3,10^ 2.0 ± 0.5 ^1,3,10^ 1.6 ± 0.8 ^1,3,10^ 2.05 ± 0.95 ^1,3,10^	−0.52 ^3,5^ NI −0.66 ^3,5^ NI
		*p* ^8^										ET, CT: *p <* 0.05 (pre vs. post) ET vs. ST: *p <* 0.05 (post) ET: *p =* 0.027, CT: *p =* 0.002 (changes) ET, ST, CT vs. CG: *p =* 0.029 (post hoc) ET vs. ST: *p =* 0.038^19^ (post hoc)		
Tayebi et al. [58]	2016	ET ST CT	NI	5.2 ± 0.05 ^2,3,10^ 4.9± 0.05 ^2,3,10^ 4.4 ± 0.05 ^2,3,10^	NI	NI	14.1 ± 0.2 ^2,3,10^ 10.7 ± 0.15 ^2,3,10^ 15.0 ± 0.2 ^2,3,10^	NI	NI	NI	NI	NI	63 ± 2 ^2,3,10,17^ 61 ± 2 ^2,3,10,17^ 56 ± 2 ^2,3,10,17^	NI
		*p* ^8^	ET vs. ST, CT vs. ST, CT vs. ET: *p =* 0.001			ET vs. ST, CT vs. ST, CT vs. ET: *p =* 0.001						CT vs. ET: *p =* 0.016		
AbouAssi et al. ^b^ [20]	2015	ET ST CT	5.4 ± 0.8 ^1,3^ 5.5 ± 0.6 ^1,3^ 5.1 ± 0.6 ^1,3^	NI	−0.111 ± 0.55 ^1,3^ −0.017 ± 0.05 ^1,3^ 0.022 ± 0.48 ^1,3^	9.66 ± 6.0 ^1^ 8.63 ± 4.0 ^1^ 9.93 ± 5.0 ^1^	NI	−2.03 ± 3.0 ^1^ −0.22 ± 5.0 ^1^ −2.06 ± 2.3 ^1^	NI	NI	NI	2.43 ± 1.72 ^1^ 2.15 ± 1.12 ^1^ 2.21 ± 1.15 ^1^	NI	−0.59 ± 0.9 ^1^ 0.05 ± 1.3 ^1^ −0.24 ± 1.16 ^1^
		*p* ^8^				ET: *p =* 0.001 (pre vs. post) CT: *p =* 0.0005 (pre vs. post)						ET: *p =* 0.002 (pre vs. post)		
Nikseresht et al. [16]	2014	ET ST CG	5.62 ± 0.04 ^1^ 6.21 ± 0.04 ^1^ 5.92 ± 0.05 ^1^	5.36 ± 0.03 ^1^ 5.63 ± 0.07 ^1^ 5.81 ± 0.09 ^1^	NI	5.52 ± 1.72 ^1^ 5.80 ± 1.58 ^1^ 6.60 ± 1.86 ^1^	3.61 ± 1.48 ^1^ 3.66 ± 0.92 ^1^ 6.20 ± 2.64 ^1^	NI	NI	NI	NI	1.39 ± 0.44 ^1^ 1.49 ± 0.47 ^1^ 1.72 ± 0.42 ^1^	0.84 ± 0.34 ^1^ 0.84 ± 0.27 ^1^ 1.62 ± 0.56 ^1^	−0.57 ^3,5^ −0.72 ^3,5^ NI
		*p* ^8^				ET, ST: *p* ≤ 0.05 (pre vs. post) ET, ST vs. CG: *p* ≤ 0.05 (post) *p =* 0.001 (time) *p =* 0.014 (group × time) *p =* 0.012 (group)						ET, ST: *p* ≤ 0.05 (pre vs. post) ET, ST vs. CG: *p* ≤ 0.05 (post) *p =* 0.001 (time) *p =* 0.006 (group × time) *p =* 0.003 (group)		
Donges et al. [62]	2013	ET ST CT CG	5.62 ± 0.14 ^17^ 5.35 ± 0.13 ^17^ 5.53 ± 0.15 ^17^ 5.48 ± 0.19 ^17^	NI	NI	12.8 ± 2.3 ^17^ 11.5 ± 1.8 ^17^ 13.1 ± 2.9 ^17^ 10.4 ± 2.5 ^17^	NI	NI	5.4 ± 0.1 ^17^ 5.3 ± 0.1 ^17^ 5.3 ± 0.1 ^17^ 5.4 ± 0.1 ^17^	NI	NI	NI	NI	NI
Kadoglou et al. [63]	2013	ET ST CT CG	11.59 ± 2.88 ^1^ 10.54 ± 1.55 ^1^ 11.15 ± 2.88 ^1^ 9.87 ± 1.99 ^1^	NI	−3.21 ± 1.49 ^1^ −0.99 ± 0.38 ^1^ −3.71 ± 1.60 ^1^ −0.33 ± 0.61 ^1^	6.96 ± 2.72 ^1^ 7.82 ± 1.84 ^1^ 7.46 ± 2.99 ^1^ 8.93 ± 2.12 ^1^	NI	−2.97 ± 0.84 ^1^ −2.05 ± 0.75 ^1^ −4.22 ± 1.57 ^1^ −0.22 ± 0.59 ^1^	8.3 ± 1.1 ^1^ 8 ± 0.7 ^1^ 8.2 ± 1 ^1^ 7.8 ± 0.8 ^1^	NI	−0.6 ± 0.1 ^1^ −0.2 ± 0.05 ^1^ −0.9 ± 0.4 ^1^ −0.05 ± 0.01 ^1^	3.59 ± 0.66 ^1^ 3.67 ± 0.78 ^1^ 3.7 ± 1.04 ^1^ 3.92 ± 0.43 ^1^	NI	−2.11 ± 0.87 ^1^ −1.22 ± 0.34 ^1^ −2.63 ± 0.43 ^1^ −0.21 ± 0.05 ^1^
		*p* ^8^	ET, ST, CT: *p <* 0.05 (pre vs. post) CT vs. CG: *p <* 0.001 (changes) CT vs. ST: *p =* 0.032 (changes) ET vs. CG: *p =* 0.008 (changes) ST vs. CG: *p =* 0.018 (changes) ET vs. ST: *p <* 0.05 (changes)			ET, ST, CT: *p <* 0.05 (pre vs. post) CT vs. CG: *p <* 0.001 (changes) CT vs. ST: *p =* 0.007 (changes) ET vs. CG: *p <* 0.001 (changes) ST vs. CG: *p =* 0.019 (changes) ET vs. ST: *p <* 0.05 (changes)			ET, ST, CT: *p <* 0.05 (pre vs. post) CT vs. CG: *p <* 0.001 (changes) CT vs. ST: *p =* 0.043 (changes) ET vs. CG: *p =* 0.002 (changes) ST vs. CG: *p =* 0.048 (changes) ET vs. ST: *p <* 0.05 (changes)			ET, ST, CT: *p <* 0.05 (pre vs. post) CT vs. CG: *p <* 0.001 (changes) CT vs. ST: *p <* 0.001 (changes) ET vs. CG: *p <* 0.001 (changes) ST vs. CG: *p <* 0.001 (changes) ET vs. ST: *p <* 0.05 (changes)		
Venojärvi et al. [65]	2013	ET ST CG	6.2 ± 0.1 ^17^ 6.1 ± 0.1 ^17^ 6.1 ± 0.1 ^17^	NI	−0.0 ± 0.1 ^17^ −0.1 ± 0.1 ^17^ −0.2 ± 0.1 ^17^	12.6 ± 1.2 ^17^ 12.9 ± 0.7 ^17^ 7.7 ± 0.7 ^17^	NI	−1.7 ± 1.0 ^17^ −0.8 ± 1.0 ^17^ 1.0 ± 0.9 ^17^	5.5 ± 0.1 ^17^ 5.4 ± 0.1 ^17^ 5.4 ± 0.1 ^17^	NI	0.0 ± 0.1 ^17^ 0.1 ± 0.1 ^17^ 0.2 ± 0.1 ^17^	3.5 ± 0.4 ^17^ 3.6 ± 0.5 ^17^ 2.1 ± 0.2 ^17^	NI	−0.5 ± 0.3 ^17^ −0.3 ± 0.3 ^17^ 0.3 ± 0.3 ^157^
		*p* ^8^				CG vs. ET; CG vs. ST: *p =* 0.002 (0.006, 0.006) ^28^ (pre)						CG vs. ET; CG vs. ST: *p =* 0.004 (0.012, 0.015) ^28^ (pre)		
Ho et al. [66]	2012	ET	5.68 ± 0.17 ^1^	5.78 ± 0.18 ^1,9^	NI	13.05 ± 1.01 ^22^	16.67 ± 1.48 ^9,22^	NI	NI	NI	NI	1.72 ± 0.52 ^22^	NI	NI
				5.73 ± 0.10 ^1,21^			15.87 ± 1.86 ^21,22^							
		ST	5.81 ± 0.46 ^1^	5.81 ± 0.17 ^1,9^		13.98 ± 1.40 ^22^	16.82 ± 1.33 ^9,22^					1.86 ± 0.18 ^22^		
				5.77 ± 0.16 ^1,21^		14.24 ± 1.03 ^22^	13.48 ± 1.24 ^21,22^							
		CT	5.38 ± 0.13 ^1^	5.31 ± 0.10 ^1,9^			17.07 ± 1.33 ^9,22^					1.86 ± 0.13 ^22^		
				5.55 ± 0.13 ^1,21^		14.89 ± 2.29 ^22^	14.25 ± 1.25 ^21,22^							
		CG	5.35 ± 0.13 ^1^	5.46 ± 0.10 ^1,9^			14.82 ± 1.66 ^9,22^					1.92 ± 0.28 ^22^		
				5.26 ± 0.18 ^1,21^			14.76 ± 1.69 ^21,22^							
		*p* ^8^				ET, ST, CT: *p <* 0.05 (pre vs. post)								
Stensvold et al. ^c^ [6]	2012	ET ST CG	6.0 ± 1.1 ^1^ 6.6 ± 2.0 ^1^ 6.2 ± 2.1 ^1^	NI	NI	NI	NI	−0.48 (2.11–1.81) ^10,23^ 0.68 (7.45–2.49) ^10,23^ 2.66 (7.03–1.96) ^10,23^	NI	NI	NI	NI	NI	NI
Sukala et.al. [68]	2012	ET ST	10.2 ± 3.3 ^1^ 9.5 ± 3.5 ^1^	10.4 ± 2.9 ^1^ 11.4 ± 4 ^1^	0.2 ± 1.6 ^1^ 1.9 ± 3.2 ^1^	25.54 ± 11.88 ^1,3^ 20.26 ± 14.41 ^1,3^	19.41 ± 9.23 ^1,3^ 19.31 ± 14.85 ^1,3^	−6.15 ± 9.47 ^1,3^ −0.95 ± 3.46 ^1,3^	8.9 ± 1.9 ^1^ 10.7 ± 2.1^1^	8.8 ± 2.1 ^1^ 10.6 ± 2.4 ^1^	−0.1 ± 0.6 ^1^ −0.1 ± 1.1 ^1^	3.9 ± 1.9 ^1^ 2.9 ± 2 ^1^	2.9 ± 1.3 ^1^ 2.9 ± 1.9 ^1^	−0.9 ± 1.6 ^1^ 0.0 ± 0.5 ^1^
		*p* ^8^				ET: *p =* 0.09 (pre vs. post)								
Bateman et al. ^b^ [13]	2011	ET ST CT	5.35 ± 0.74 ^1,3^ 5.54 ± 0.64 ^1,3^ 5.02 ± 0.51 ^1,3^	NI	−0.22 ± 9.54 ^1^ −0.37 ± 9.22 ^1^ 1.86 ± 7.95 ^1^	NI	NI	NI	NI	NI	NI	NI	NI	NI
Jorge et al. [69]	2011	ET ST CT CG	8.14 ± 2.33 ^1,3^ 10.79 ± 4.42 ^1,3^ 8.59 ± 2.35 ^1,3^ 8.27 ± 2.40 ^1,3^	7.04 ± 2.00 ^1,3^ 9.23 ± 3.37 ^1,3^ 7.89± 2.04 ^1,3^ 6.94 ± 1.14 ^1,3^	NI	NI	NI	NI	7.63 ± 1.70 ^1^ 8.51 ± 2.45 ^1^ 7.6 ± 1.12 ^1^ 6.94 ± 0.74 ^1^	7.42 ± 1.48 ^1^ 8.24 ± 2.13 ^1^ 7.53 ± 1.05 ^1^ 7.07 ± 0.70 ^1^	NI	2.45 ± 1.31 ^1^ 4.54 ± 3.94 ^1^ 3.14 ± 2.12 ^1^ 3.91 ± 4.42 ^1^	2.24 ± 1.52 ^1^ 4.07 ± 2.90 ^1^ 2.59 ± 1.31 ^1^ 4.28 ± 5.74 ^1^	NI
		*p* ^8^	ET, ST, CT, CG: *p <* 0.05 (pre vs. post)											
Slentz et al. ^b^ [15]	2011	ET ST CT	NI	NI	NI	NI	NI	NI	NI	NI	NI	2.37 ± 1.6 ^1^ 2.08 ± 1.1 ^1^ 2.12 ± 1.2 ^1^	NI	−0.40 ± 0.8 ^1^ −0.09 ± 1.3 ^1^ −0.50 ± 0.9 ^1^
		*p* ^8^										ET: *p =* 0.004 (pre vs. post) CT: *p =* 0.002 (pre vs. post)		
Gram et al. [70]	2010	ET	NI	NI	NI	NI	NI	NI	7.2 ± 1.0 ^1^	6.9 ± 0.2^14,24^	NI	NI	NI	NI
										7.0 ± 0.2^14,25^				
		CT							7.2 ± 0.9 ^1^	7.2 ± 0.2^14,24^				
										7.5 ± 0.3^14,25^				
		CT							7.2 ± 0.9 ^1^	7.9 ± 0.3 ^14,24^				
										7.6 ± 0.3 ^14,25^				
Stensvold et al. ^c^ [22]	2010	ET ST CT CG	6.0 ± 1.1 ^1^ 6.6 ± 2.0 ^1^ 6.0 ± 2.4 ^1^ 6.2 ± 2.1 ^1^	5.9 ± 0.8 ^1^ 6.6 ± 1.5 ^1^ 5.6 ± 1.8 ^1^ 6.1 ± 2.3 ^1^	−0.2 (−0.71–0.35) ^26^ 0.1 (−0.5–0.6) ^26^ −0.4 (−1.0–0.3) ^26^ −0.1 (−0.68–0.43) ^26^	NI	NI	NI	6.19 ± 0.80 ^1^ 6.44 ± 0.95 ^1^ 6.28 ± 0.78 ^1^ 6.12 ± 1.62 ^1^	5.95 ± 0.66 ^1^ 6.47 ± 1.04 ^1^ 6.30 ± 0.76 ^1^ 6.24 ± 1.40 ^1^	−0.25 (−0.51–0.01) ^26^ 0.03 (−0.21–0.33) ^26^ 0.03 (−0.28–0.33) ^26^ 0.10 (−0.17–0.37) ^26^	40.4 ± 8.3 ^1,16^ 38.1 ± 17.4 ^1,16^ 42.7 ± 24.8 ^1,16^ 41.0 ± 12.8 ^1,16^	48.4 ± 17.1 ^1,16^ 46.5 ± 24.4 ^1,16^ 44.9 ± 17.6 ^1,16^ 44.3 ± 20.4 ^1,16^	8.0 (−2.7–18.8) ^16,26^ 7.5 (−3.8–18.8) ^16,26^ 3.0 (−9.6–15.6) ^16,26^ 3.5 (−7.8–14.7) ^16,26^
Ahmadizad et al. [71]	2007	ET ST CG	5.21 ± 0.77 ^1^ 5.09 ± 0.65 ^1^ NI	5.16 ± 1.0 ^1^ 5.09 ± 0.64 ^1^ NI	NI	8.54 ± 4.75 ^1^ 10.55 ± 3.57 ^1^ NI	5.73 ± 3.24 ^1^ 6.41 ± 3.07 ^1^ NI	NI	NI	NI	NI	2.0 ± 0.7 ^1,10^ 2.4 ± 0.7 ^1,10^ 2.25 ± 0.75 ^1,10^	1.7 ± 0.3 ^1,10^ 1.5 ± 0.8 ^1,10^ 2.5 ± 0.7 ^1,10^	−0.7 ^3,5^ −0.9 ^3,5^ NI
		*p* ^8^				*p <* 0.05 (pre vs. post)						ET vs. CG, ST vs. CG: *p <* 0.05 (post) *p <* 0.05 (pre vs. post) ET vs. CG, ST vs. CG: *p <* 0.05 (changes)		
Hara et al. [72]	2005	ET CT CG	5.3 ± 0.3 ^1,3^ 5.6 ± 0.5 ^1,3^ 5.9 ± 0.8 ^1,3^	5.2 ± 0.1 ^1,3^ 5.2 ± 0.3 ^1,3^ 5.6 ± 0.5 ^1,3^	NI	16.0 ± 6.5 ^1^ 8.4 ± 2.9 ^1^ 15.0 ± 4.5 ^1^	11.0 ± 4.1 ^1^ 8.0 ± 0.6 ^1^ 16.6 ± 5.9 ^1^	NI	NI	NI	NI	3.78 ± 1.62 ^1^ 2.15 ± 0.89 ^1^ 3.78 ± 0.73 ^1^	2.54 ± 0.93 ^1^ 1.85 ± 0.25 ^1^ 3.92 ± 1.43 ^1^	NI
		*p* ^8^				CG vs. CT, ET vs. CT: *p <* 0.05 (pre)						CG vs. CT, ET vs. CT: *p <* 0.05 (pre)		
Banz et al. [73]	2003	ET ST	6.23 ± 1.43 ^1,3^ 8.95 ± 2.9 ^1,3^	6.48 ± 1.29 ^1,3^ 8.07 ± 2.93 ^1,3^	NI	3.24 ± 0.98 ^3^ 2.92 ± 1.43 ^3^	2.89 ± 1.27 ^3^ 2.97 ± 1.17 ^3^	NI	NI	NI	NI	NI	NI	NI
Cuff et al. [74]	2003	ET CT CG	NI	NI	NI	NI	NI	NI	6.3 ± 0.2 ^17^ 6.9 ± 0.4 ^17^ 6.9 ± 0.4 ^17^	NI	−0.10 ± 0.11 ^22^ −0.1 ± 0.22 ^22^ −0.03 ± 0.20 ^22^	NI	NI	NI

CG—control group; CT—combined training; ET—endurance training; NI—no information; ST—strength training. ^1^ Mean ± standard deviation; ^2^ Adjusted means; ^3^ Units converted; ^4^ The study from 2021; ^5^ Mean; ^6^ The study from 2019; ^7^ Mean and 95% CI; ^8^ Only significant values were presented; ^9^ 8 weeks; ^10^ Data from figure; ^11^ Changes (post-intervention value minus pre-intervention value); ^12^ Mild intensity continuous training; ^13^ High-intensity interval training; ^14^ Least square means (means adjusted for baseline) with (95% CI); ^15^ mU/mL; ^16^ %; ^17^ Mean ± standard error; ^18^ µU/dL; ^19^ Wrong value; ^20^ Data shown as log value; ^21^ 12 weeks; ^22^ Means ± standard error of the mean; ^23^ Median and range; ^24^ 16 weeks; ^25^ 48 weeks; ^26^ Estimated margins of the mean (95% CI); ^28^ Bonferroni correction; ^a–c^ Studies marked with the same letters were conducted in the same population.

**Table 4:** Glucose and insulin metabolism parameters in studied populations.

Author	Year	Groups	2 h Glucose [mmol/L]			2 h Insulin [µU/mL]			C-Peptide [nmol/l]		
			Pre	Post	Changes	Pre	Post	Changes	Pre	Post	Changes
Donges et al. [62]	2013	ET	4311 ± 410 ^1,2^	3035 ± 384 ^1,2^	NI	9167 ± 1222 ^1,2^	5304 ± 560 ^1,2^	NI	0.93 ± 0.11 ^2,3^	NI	NI
		ST	4812 ± 690 ^1,2^	3765 ± 436 ^1,2^		7857 ± 1425 ^1,2^	6080 ± 1018 ^1,2^		0.86 ± 0.07 ^2,3^		
		CT	4594 ± 820 ^1,2^	3958 ± 718 ^1,2^		6342 ± 764 ^1,2^	5075 ± 763 ^1,2^		0.80 ± 0.06 ^2,3^		
		CG	4607 ± 667 ^1,2^	4714 ± 974 ^1,2^		5591 ± 1019 ^1,2^	6411 ± 1222 ^1,2^		0.81 ± 0.14 ^2,3^		
		*p* ^4^	ET: *p <* 0.05 (pre vs. post)			CG vs. ET: *p <* 0.05 (pre, post hoc) ET, CT: *p <* 0.05 (pre vs. post)					
Venojärvi et al. [65]	2013	ET	6.8 ± 0.3 ^2^	NI	−0.5 ± 0.3 ^2^	80.7 ± 10.1 ^2^	NI	−15.9 ± 6.4 ^2^	NI	NI	NI
		ST	6.6 ± 0.3 ^2^		−0.3 ± 0.3 ^2^	63.0 ± 8.2 ^2^		−4.2 ± 4.8 ^2^			
		CG	6.1 ± 0.2 ^2^		−0.3 ± 0.3 ^2^	48.9 ± 6.7 ^2^		−9.1 ± 3.9 ^2^			
		*p* ^4^				*p =* 0.036 (pre) CG vs. ET: *p =* 0.042 ^5^ (pre, post hoc)					
Sukala et al. [68]	2012	ET	NI	NI	NI	NI	NI	NI	1.4 ± 0.3 ^6^	1.5 ± 0.7 ^6^	0.1 ± 0.5 ^6^
		ST							1.6 ± 1.1 ^6^	1.6 ± 1 ^6^	0.1 ± 0.5 ^6^
Stensvold et al. [22]	2010	ET	NI	NI	NI	NI	NI	NI	1.11 ± 0.24 ^6^	1.00 ± 0.42 ^6^	−0.33 (−0.60–(−0.06)) ^7^
		ST							1.88 ± 2.37 ^6^	1.10 ± 0.46 ^6^	−0.29 (−0.58–0.1) ^7^
		CT							1.30 ± 0.68 ^6^	1.08 ± 0.30 ^6^	−0.27 (−0.5–0.49) ^7^
		CG							1.12 ± 0.26 ^6^	1.18 ± 0.58 ^6^	−0.15 (−0.44–(−0.14)) ^7^
		*p* ^4^							ET: *p <* 0.05 (pre vs. post)		

CG—control group; CT—combined training; ET—endurance training; ST—strength training; NI—no information. ^1^ Data from the figure, the area under the curve; ^2^ Mean ± standard error; ^3^ Converted values; ^4^ Only statistically significant values are shown; ^5^ Value after Bonferroni correction; ^6^ Mean ± standard deviation; ^7^ Estimated margins of the mean (95% confidence intervals).

**Table 5:** Lipid metabolism parameters in studied populations.

Author	Year	Group	TC [mg/dL]			LDL-C [mg/dL]			HDL-C [mg/dL]			TG [mg/dL]		
			Pre	Post	Changes	Pre	Post	Changes	Pre	Post	Changes	Pre	Post	Changes
Jamka et al. [38]	2021	ET	210 ± 48 ^1^	209 ± 45 ^1^	−2 ± 46 ^1,2^	124 ± 39 ^1^	127 ± 37 ^1^	0 ± 41 ^1,2^	55 ± 14 ^1^	55 ± 13 ^1^	0 ± 17 ^1,2^	148 ± 93 ^1^	134 ± 57 ^1^	−5 ± 111 ^1,2^
		CT	210 ± 34 ^1^	207 ± 34 ^1^	−4 ± 17 ^1,2^	122 ± 30 ^1^	121 ± 31 ^1^	−2 ± 49 ^1,2^	61 ± 13 ^1^	60 ± 12 ^1^	−1 ± 15 ^1,2^	134 ± 66 ^1^	130 ± 50 ^1^	−2 ± 118 ^1,2^
Banitalebi et al. [39]	2021	ET	NI	NI	NI	NI	NI	NI	55.43 ± 8.55 ^1^	58.50 ± 1.22 ^1^	NI	179.14 ± 79.36 ^1^	125.00 ± 21.75 ^1^	−54.14 (−93.26–−15.02) ^3^
		CT							49.07 ± 8.26 ^1^	50.79 ± 8.64 ^1^		159.07 ± 28.64 ^1^	135.07 ± 45.86 ^1^	−24.00 (−49.70–1.70) ^3^
		CG							54.50 ± 4.48 ^1^	51.21 ± 6.27 ^1^		149.21 ± 74.72 ^1^	126.00 ± 40.23 ^1^	−23.21 (−63.55–17.13) ^3^
		*p* ^4^										ET: *p =* 0.025 (pre vs. post)		
Amanat et al. ^a^ [41]	2020	ET	189.85 ± 34.77 ^1^	184.57 ± 34.52^1^	−5.29 ± 2.88 ^1,5^ −2.79 ± 1.52% ^1,6,7^	93.64 ± 16.86 ^1^	91.57 ± 16.35 ^1^	−1.95 ± 3.27 ^1,5^ −2.08 ± 3.49% ^1,6,7^	52.35 ± 10.42 ^1^	56.92 ± 10.11 ^1^	4.56 ± 7.48 ^1,5^	193.21 ± 50.12 ^1^	190.42 ± 50.37 ^1^	−2.8 ± 3.78 ^1,5^ −1.45 ± 1.96% ^1,6,7^
		ST	197.28 ± 15.96 ^1^	193.50 ± 16.44^1^	−3.84 ± 5.81 ^1,5^ −1.95 ± 2.95% ^1,6,7^	97.14 ± 34.37 ^1^	94.71 ± 34.95 ^1^	−2.32 ± 2.49 ^1,5^ −2.39 ± 2.56% ^1,6,7^	54.28 ± 12.28 ^1^	56.64 ± 12.41 ^1^	2.27 ± 5.27 ^1,5^	209.28 ± 54.95 ^1^	202.64 ± 49.81 ^1^	−6.39 ± 6.39 ^1,5^ −3.05 ± 3.05% ^1,6,7^
		CT	166.92 ± 37.76 ^1^	161.84 ± 37.56^1^	−5.12 ± 5.6 ^1,5^ −3.07± 3.35% ^1,6,7^	109.69 ± 35.94 ^1^	104.38 ± 38.04 ^1^	−5.18 ± 5.76 ^1,5^ −4.72 ± 5.25% ^1,6,7^	49.61 ± 8.93 ^1^	53.69 ± 9.14 ^1^	3.99 ± 3.47 ^1,5^	199.92 ± 34.17 ^1^	191.76 ± 35.04 ^1^	−8,13 ± 5.04 ^1,5^ −4.07 ± 2.52% ^1,6,7^
		CG	184.85 ± 25.42 ^1^	186.42 ± 27.02^1^	1.59 ± 3.85 ^1,5^ 0.86 ± 2.08% ^1,6,7^	118.75 ± 32.25 ^1^	120.59 ± 33.13 ^1^	1.92 ± 2.88 ^1,5^ 1.61 ± 2.43% ^1,6,7^	50.92 ± 11.71 ^1^	52.50 ± 13.24 ^1^	1.53 ± 4.18 ^1,5^	175.63 ± 27.01 ^1^	177.13 ± 29.05 ^1^	1.56 ± 3.78 ^1,5^ 0.89 ± 2.15% ^1,6,7^
		*p* ^4^	*p =* 0.037 (post) ET: *p <* 0.001, ST: *p =* 0.030, CT: *p =* 0.003 (pre vs. post) ET, ST, CT vs. CG: *p <* 0.05 (changes, post hoc) ET, CT vs. CG: *p <* 0.05 (post, post hoc)			ET: *p =* 0.034, ST, CT: *p =* 0.003 (pre vs. post) CT vs. CG: *p <* 0.05 (post, post hoc) CT, ST vs. CG: *p <* 0.05 (changes, post hoc)			ET: *p =* 0.041, CT: *p <* 0.001 (pre vs. post)			ET: *p =* 0.017, CT: *p <* 0.001 (pre vs. post) CT vs. GC: *p <* 0.05 (post, post hoc) ST, CT vs. CG: *p <* 0.05 (changes, post hoc)		
Dianatinasab et al.^a^ [42]	2020	ET	161.25 ± 10.12 ^1^	157.00 ± 9.12 ^1^	−4.4 ^5^	131.58 ± 11.96 ^1^	129.50 ± 12.74 ^1^	NI	51.91 ± 9.26 ^1^	51.08 ± 9.03 ^1^	NI	154.41 ± 12.08 ^1^	140.75 ± 12.67 ^1^	−14.34 ^5^
		ST	169.25 ± 14.55 ^1^	167.33 ± 12.85 ^1^	−3.13 ^5^	129.58 ± 11.98 ^1^	126.33 ± 12.04 ^1^		58.16 ± 13.24 ^1^	58.83 ± 14.07 ^1^		155.41 ± 11.27 ^1^	148.66 ± 10.94 ^1^	−8.36 ^5^
		CT	153.41 ± 13.05 ^1^	147.83 ± 13.20 ^1^	−5.78 ^5^	122.41 ± 11.03 ^1^	117.25 ± 11.29 ^1^		47.25 ± 8.40 ^1^	50.08 ± 8.45 ^1^		155.41 ± 12.68 ^1^	142.75 ± 8.33 ^1^	−13.57 ^5^
		CG	152.23 ± 8.47 ^1^	151.11 ± 9.85 ^1^	−0.27 ^5^	136.61 ± 12.37 ^1^	137.30 ± 11.75 ^1^		51.84 ± 9.59 ^1^	52.00 ± 10.97 ^1^		160.53 ± 11.70 ^1^	160.00 ± 13.44 ^1^	−1.04 ^5^
		*p* ^4^	ET: *p =* 0.033, CT: *p =* 0.022 (pre vs. post) CT vs. CG: *p <* 0.05 (post, change, post hoc)						CT: *p =* 0.050 (pre vs. post)			*p =* 0.02 (time × group) ET: *p =* 0.011, ST: *p =* 0.022, CT: *p =* 0.011 (pre vs. post) ET, ST, CT vs. CG: *p <* 0.05 (post, post hoc)		
Dupuit et al. [37]	2020	ET ^8^	244 ± 50 ^1,6^	244 ± 50 ^1,6^	NI	135 ± 62 ^1,6^	139 ± 54 ^1,6^	NI	65.7 ± 15.5 ^1,6^	61.9 ± 3.9 ^1,6^	NI	124 ± 97.4 ^1,6^	97.4 ± 44.2 ^1,6^	NI
		ET ^9^	217 ± 43 ^1,6^	209 ± 46 ^1,6^		128 ± 31 ^1,6^	131 ± 31 ^1,6^		65.7 ± 34.8 ^1,6^	65.7 ± 19.3 ^1,6^		106.3 ± 62 ^1,6^	79.7 ± 35.4 ^1,6^	
		CT	240 ± 39 ^1,6^	240 ± 39 ^1,6^		151 ± 39 ^1,6^	151 ± 31 ^1,6^		65.7 ± 19.3 ^1,6^	65.7 ± 15.5 ^1,6^		106.3 ± 44.2 ^1,6^	106.3 ± 53.2 ^1,6^	
		*p* ^4^										*p <* 0.05 (time)		
Kim et al. [43]	2020	ET	199.85 ± 42.75 ^1^	195.15 ± 37.83 ^1^	−4.69 ± 11.94 ^1^	119.62 ± 50.81 ^1^ −2.3 ± 6.0% ^1,6,7^	117.46 ± 41.22 ^1^	−2.15 ± 17.03 ^1^ −1.80± 14.27% ^1,6,7^	56.85 ± 0.09 ^1^	57.92 ± 13.48 ^1^	1.08 ± 5.27 ^1^ 1.90 ± 9.27% ^1,6,7^	127.39 ± 89.30 ^1^	120.31 ± 99.20 ^1^	−7.08 ± 66.47 ^1^ −5.56 ± 52.20% ^1,6,7^
		ST	205.21 ± 37.77 ^1^	201.43 ± 44.88 ^1^	−3.79 ± 22.98 ^1^	129.93 ± 32.40 ^1^ −1.9 ± 11.2% ^1,6,7^	124.71 ± 39.58 ^1^	−5.21 ± 23.43 ^1^ −4.01 ± 18.03% ^1,6,7^	52.00 ± 9.81 ^1^	52.14 ± 10.17 ^1^	0.14 ± 6.97 ^1^ 0.27 ± 13.40% ^1,6,7^	147.79 ± 112.30 ^1^	136.93 ± 77.97 ^1^	−10.86 ± 50.14 ^1^ −7.35 ± 33.93% ^1,6,7^
Christensen et al. [44]	2019	ET	185.6 ± 34.8 ^1^	189 (178–201) ^6,10^	−3.3 (−13.3–6.7) ^6,8^ −1.2 (−6.3–4.0)% ^7,8^	119.9 ± 23.2 ^1^	100 (93.3–106.7) ^6,8^	−3.33 (−10–3.33) ^6,10^ −3.6 (−10.9–3.70)% ^7,10^	54.1 ± 11.6 ^1^	50.3 (46.4–54.1) ^6,10^	0.0 (−3.86–3.86) ^6,10^ −0.3 (−6.0–5.4)% ^7,10^	NI	NI	NI
		ST	185.6 ± 30.9 ^1^	189 (178–201) ^6,10^	−3.3 (−13.3–6.7) ^6,10^ −2.3 (−7.6–3.1)% ^7,10^	119.9 ± 27.1 ^1^	96.7 (90–106.7) ^6,8^	−6.67 (−13.33–0.0) ^6,10^ −6.0 (−13.5–1.6) % ^7,10^	50.3 ± 7.7 ^10^	50.3 (46.4–50.3) ^6,10^	0.0 (−3.86–0.0) ^6,10^ −3.2 (−9.1–2.7)% ^7,10^			
		CG	197.2 ± 30.9 ^1^	182 (4.4–193) ^6,10^	3.3 (−6.7–13.3) ^6,10^ 1.1 (−4.7–7.0)% ^7,10^	127.6 ± 30.9 ^1^	110 (100–116.7) ^6,10^	3.33 (−3.33–13.33) ^6,10^ 5.0 (−3.2–13.2)% ^7,10^	46.4 ± 11.6 ^1^	50.3 (46.4–54.1) ^6,10^	0.0 (−3.86–3.86) ^6,10^ 2.3 (−4.0–8.7)% ^7,10^			
Keihanian et al. [45]	2019	ET	193.6 ± 12.9 ^1^	158.2 ± 17.8 ^1^	−35.2 ^6^ −18.2% ^7^	101.5 ± 6.7 ^1^	89.8 ± 5.4 ^1^	−11.67 ^6^ −11.5% ^7^	34.9 ± 6.3 ^1^	42.8 ± 7.6 ^1^	7.88 ^6^ 22.6% ^7^	199.2 ± 12.3 ^1^	163.9 ± 17.5 ^1^	−35.26 ^6^ −17.7% ^7^
		ST	187.8 ± 20 ^1^	161 ± 20.7 ^1^	−26.7 ^6^ −14.2% ^7^	102.6 ± 10.1 ^1^	91.5 ± 9.3 ^1^	−11.08 ^6^ −10.8% ^7^	32.9 ± 6.9 ^1^	39.9 ± 7.1 ^1^	6.97 ^6^ 21.2% ^7^	216.6 ± 29.2 ^1^	188.9 ± 30.1 ^1^	−27.51 ^6^ −12.7% ^7^
		CG	184.5 ± 18.9 ^1^	180.8 ± 17 ^1^	−3.7 ^6^ −2% ^7^	100 ± 8.1 ^1^	98.5 ± 8.2 ^1^	−1.5 ^6^ −1.5% ^7^	35.5 ± 7 ^1^	35 ± 6.3 ^1^	−0.04 ^6^ −0.1% ^7^	184.6 ± 37.5 ^1^	183.5 ± 35.6 ^1^	−1.11 ^6^ −0.6% ^7^
		*p* ^4^	ET, ST: *p <* 0.05 (pre vs. post) ET, ST vs. CG: *p <* 0.05, ET vs. ST: *p <* 0.05 (post, post hoc)			ET, ST: *p <* 0.05 (pre vs. post) ET, ST vs. CG: *p <* 0.05 (post, post hoc)			ET, ST: *p <* 0.05 (pre vs. post) ET, ST vs. CG: *p <* 0.05 (post, post hoc)			ET, ST: *p <* 0.05 (pre vs. post) ET, ST vs. CG: *p <* 0.05, ET vs. ST: *p <* 0.05 (post, post hoc)		
Mohammad Rahimi et al. [46]	2019	ET	221.0 ± 14.6 ^1^	179.3 ± 14.9 ^1^	NI	174.4 ± 14.6 ^1^	120.1 ± 17.0 ^1^	NI	32.6 ± 3.6 ^1^	49.6 ± 3.7 ^1^	NI	164.5 ± 16.8 ^1^	121.2 ± 11.3 ^1^	NI
		ST	222.2 ± 15.6 ^1^	211.1 ± 15.4 ^1^		173.6 ± 12.7 ^1^	169.6 ± 12.4 ^1^		34.1 ± 5.3 ^1^	36.7 ± 5.8 ^1^		165.8 ± 15.4 ^1^	152.8 ± 14.8 ^1^	
		CT	225.5 ± 13.3 ^1^	181.0 ± 16.4 ^1^		175.2 ± 12.4 ^1^	140.9 ± 10.6 ^1^		33.9 ± 4.0 ^1^	46.7 ± 7.0 ^1^		167.3 ± 13.5 ^1^	125.4 ± 10.5 ^1^	
		CG	223.4 ± 17.6 ^1^	225.3 ± 16.1 ^1^		173.5 ± 11.1 ^1^	174.2 ± 12.1 ^1^		34.6 ± 4.3 ^1^	34.8 ± 4.1 ^1^		163.6 ± 14.8 ^1^	162.6 ± 13.7 ^1^	
		*p* ^4^	ET, ST, CT: *p <* 0.05 (pre vs. post) ET, CT vs. CG: *p <* 0.05; ET, CT vs. ST: *p <* 0.05 (post, post hoc)			ET, ST, CT: *p <* 0.05 (pre vs. post) ET, CT vs. CG: *p <* 0.05; ET, CT vs. ST: *p <* 0.05 (post, post hoc)			ET, ST, CT: *p <* 0.05 (pre vs. post) ET, CT vs. CG: *p <* 0.05; ET vs. ST: *p <* 0.05 (post, post hoc)			ET, ST, CT: *p <* 0.05 (pre vs. post) ET, CT vs. CG: *p <* 0.05; ET vs. ST: *p <* 0.05 (post, post hoc)		
Ratajczak et al. [47]	2019	ET	217.3 ± 39.6 ^1,6^	203.8 ± 37.7 ^1,6^	NI	130.4 ± 30.8 ^1,6^	121.5 ± 33.8 ^1,6^	NI	51.2 ±13.8 ^1,6^	55.8 ± 13.1 ^1,6^	NI	133.74 ± 54.91 ^1,6^	136.40 ± 66.43 ^1,6^	NI
		CT	225 ± 41.2 ^1,6^	212.3 ± 41.9 ^1,6^		138.1 ± 26.9 ^1,6^	126.9 ± 29.6 ^1,6^		55.4 ± 21.5 ^1,6^	60 ± 18.8 ^1,6^		117.80 ± 46.06 ^1,6^	116.03 ± 51.37 ^1,6^	
		*p* ^4^	ET, CT: *p <* 0.05 (pre vs. post)			CT: *p <* 0.05 (pre vs. post)			ET: *p <* 0.01 (pre vs. post)					
Roberson et al. [49]	2018	ET	NI	NI	NI	NI	NI	NI	51.5 ± 5.4 ^11^	55.9 ± 6.6 ^11^ 56.5 ± 2.6 ^2^	4.38 ± 1.76 ^11^	147.5 ± 19.8 ^11^	138.4 ± 19.9 ^11^ 128.0 ± 14.2 ^2^	NI
		ST							50.2 ± 3.0 ^11^	54.1 ± 3.6 ^11^ 56.1 ± 2.5 ^2^	NI	127.9 ± 16.8 ^11^	117.8 ± 11.3 ^11^ 125.8 ± 13.3^2^	
		CG							55.7 ± 5.2 ^11^	61.2 ± 6.2 ^11^ 57.4 ± 3.0 ^2^		134.5 ± 30.4 ^11^	142.5 ± 42.1 ^11^ 144.3 ± 16.3 ^2^	
		*p* ^4^							ET: *p =* 0.04 (pre vs. post)					
Arslan et al. [52]	2017	ET	195.7 ± 13.8 ^1^	181.5 ± 13.6 ^1^	−15.2 ^6^ −7.9% ^7^	119.6 ± 13.8 ^1^	114.8 ± 13.5 ^1^	−4.8 ^6^ −4.4% ^7^	54.2 ± 7.9 ^1^	57.0 ± 8.0 ^1^	2.80 ^6^ 4.8% ^7^	107.9 ± 12.3 ^1^	102.0 ± 12.1 ^1^	−9 ^6^ −5.8% ^7^
		CT	207.1 ± 18.5 ^1^	188.8 ± 15.2 ^1^	−18.3 ^6^ −9.7% ^7^	110.2 ± 13.4 ^1^	105.3 ± 13.8 ^1^	−4.9 ^6^ −4.6% ^7^	53.1 ± 8.3 ^1^	56.0 ± 8.7 ^1^	2.9 ^6^ 5.3% ^7^	101.3 ± 14.6 ^1^	94.8 ± 14.4 ^1^	−6.5 ^6^ −6.9% ^7^
		CG	200.3 ± 18.9 ^1^	202.2 ± 19.1 ^1^	1.9 0.9% ^7^	112.2 ± 12.9 ^1^	115.6 ± 13.3 ^1^	3.4 ^6^ 2.9% ^7^	51.6 ± 8.1 ^1^	50.8 ± 7.5 ^1^	−0.8 ^6^ −1.4% ^7^	103.4 ± 9.1 ^1^	105.6 ± 8.6 ^1^	2.2 ^6^ 2.0% ^7^
		*p* ^4^	ET, CT vs. CG: *p =* 0.022 (changes, post hoc)									ET, CT vs. CG: *p =* 0.012 (changes, post hoc)		
Oh et al. [53]	2017	ET ^9^	NI	NI	NI	NI	NI	NI	NI	NI	NI	130.62 ± 1.12 ^1,6,12^	123.31 ± 1.10 ^1,6,12^	−0.025 ^12^
		ET ^8^										151.01 ± 1.22 ^1,6,12^	128.53 ± 1.17 ^1,6,12^	−0.070 ^12^
		ST										110.15 ± 1.15 ^1,6,12^	114.29 ± 1.15 ^1,6,12^	0.016 ^12^
Said et al. [54]	2017	ET	207.5 ± 14.36 ^1^	196.5 ± 17.88 ^1^	−14.0 ± 12. 1 ^1,6^ −6.75 ± 5.84% ^1,7^	152.4 ± 15.3 ^1^	143.6 ± 16.6 ^1^	−9.30 ± 6.49 ^1,6^ −6.1 ± 4.26% ^1,7^	47.2 ± 2.7 ^1^	51.4 ± 3.62 ^1^	4.48 ± 2.55 ^1,6^ 9.5 ± 5.4% ^1,7^	133.8 ± 5.1 ^1^	121.8 ± 8.3 ^1^	−15.30 ± 7.65 ^1,6^ −11.57 ± 5.75% ^1,7^
		CT	203.4 ± 21.40 ^1^	195.0 ± 19.02 ^1^	−10.6 ± 9.2 ^1,6^ −5.23 ± 4.51% ^1,7^	147.7 ± 18.13 ^1^	140.9 ± 13.25 ^1^	−7.55 ± 6.20 ^1,6^ −5.11 ± 4.2% ^1,7^	48.4 ± 6.7 ^1^	52.2 ± 4.5 ^1^	4.02 ± 6.3 ^1,6^ 8.3 ± 6.3% ^1,7^	130.7 ± 12.07 ^1^	119.0 ± 11.68 ^1^	−13.16 ± 12.46 ^1,6^ −10.07 ± 9.53% ^1,7^
		*p* ^4^	ET, CT: *p <* 0.05 (pre vs. post)			ET, CT: *p <* 0.05 (pre vs. post)			ET, CT: *p <* 0.01 (pre vs. post)			CT: *p <* 0.05; ET: *p <* 0.01 (pre vs. post)		
Soori et al. [55]	2017	ET	242 ± 23 ^1^	215 ± 19 ^1^	NI	169 ± 22 ^1^	139 ± 22 ^1^	NI	50.9 ± 8.7 ^1^	58.6 ± 10.4 ^1^	NI	120 ± 20 ^1^	102 ± 19 ^1^	NI
		ST	253 ± 49 ^1^	250 ± 48 ^1^		170 ± 38 ^1^	167 ± 43 ^1^		59.5 ± 6.8 ^1^	58.6 ± 6.9 ^1^		116 ± 52 ^1^	118 ± 48 ^1^	
		CT	233 ± 40 ^1^	198 ± 39 ^1^		153 ± 45 ^1^	160 ± 39 ^1^		47.4 ± 11.7 ^1^	55.3 ± 15.1 ^1^		115 ± 27 ^1^	87 ± 10 ^1^	
		CG	217 ± 25 ^1^	238 ± 39 ^1^		145 ± 27 ^1^	167 ± 34 ^1^		50.9 ± 7.1 ^1^	50.5 ± 6.6 ^1^		107 ± 54 ^1^	105 ± 25 ^1^	
		*p* ^4^	ET, CT: *p <* 0.05 (pre vs. post) ET, ST, CT vs. CG: *p =* 0.006 (post, post hoc)						ET, CT: *p <* 0.05 (pre vs. post) ET, ST, CT vs. CG: *p =* 0.008 (post, post hoc)			ET, CT: *p <* 0.05 (pre vs. post)		
Wang [56]	2017	ET	196 ± 51 ^1,6^	121 ± 51 ^1,6^	NI	51 ± 18 ^1,6^	44 ± 24 ^1,6^	NI	46.4 ± 21.7 ^1,6^	52.2 ± 16.6 ^1,6^	NI	271 ± 40.7 ^1,6^	207.3 ± 96.5 ^1,6^	NI
		CT	197 ± 55 ^1,6^	142 ± 60 ^1,6^		60 ± 21 ^1,6^	43 ± 23 ^1,6^		45.6 ± 27.1 ^1,6^	54.9 ± 22 ^1,6^		286.1 ± 77.1 ^1,6^	215.2 ± 100.1 ^1,6^	
		CG	204 ± 48 ^1,6^	205 ± 59 ^1,6^		51 ± 18 ^1,6^	54 ± 21 ^1,6^		42.9 ± 21.7 ^1,6^	45.2 ± 23.6 ^1,6^		293.2 ± 54 ^1,6^	290.5 ± 56.7 ^1,6^	
		*p* ^4^	ET, CT: *p <* 0.05 (pre vs. post) ET, CT vs. CG: *p <* 0.05 (post, post hoc)			ET, CT: *p <* 0.05 (pre vs. post) ET, CT vs. CG: *p <* 0.05 (post, post hoc)			ET, CT: *p <* 0.05 (pre vs. post) ET, CT vs. CG: *p <* 0.05 (post, post hoc)			ET, CT: *p <* 0.05 (pre vs. post) ET, CT vs. CG: *p <* 0.05 (post, post hoc)		
Chen et al. [57]	2016	ET	209 ± 54 ^1,5,6^	205 ± 43 ^1,5,6,13^ 197 ± 54 ^1,5,6,14^	NI	147 ± 27 ^1,5,6^	135 ± 39 ^1,5,6,13^ 135 ± 39 ^1,5,6,14^	NI	42.5 ± 11.6 ^1,5,6^	39.8 ± 11.6 ^1,5,6,13^ 43.7 ± 12.4 ^1,5,6,14^	NI	141.7 ± 101.9 ^1,5,6^	141.7 ± 79.7 ^1,5,6,13^ 132.9 ± 44.3 ^1,5,6,14^	NI
		ST	193 ± 46 ^1,5,6^	201 ± 31 ^1,5,6,13^ 193 ± 43 ^1,5,6,14^		131 ± 35 ^1,5,6^	131 ± 27 ^1,5,6,13^ 124 ± 46 ^1,5,6,14^		42.2 ± 10.8 ^1,5,6^	42.5 ± 17.4 ^1,5,6,13^ 46 ± 13.9 ^1,5,6,14^		132.9 ± 88.6 ^1,5,6^	106.3 ± 53.1 ^1,5,6,13^ 106.3 ± 44.3 ^1,5,6,14^	
		CG	201 ± 46 ^1,5,6^	209 ± 46 ^1,5,6,13^ 205 ± 46 ^1,5,6,14^		135 ± 39 ^1,5,6^	143 ± 39 ^1,5,6,13^ 143 ± 39 ^1,5,6,14^		46.4 ± 11.6 ^1,5,6^	48.3 ± 13.5 ^1,5,6,13^ 46.4 ± 14.7 ^1,5,6,14^		106.3 ± 88.6 ^1,5,6^	97.4 ± 53.1 ^1,5,6,13^ 106.3 ± 53.1 ^1,5,6,14^	
		*p* ^4^							ET: *p <* 0.05 (post vs. mild) ST: *p <* 0.05 (pre vs. post) ET vs. CG: *p <* 0.5 (mild, post hoc)			ST: *p <* 0.05 (pre vs. mild)		
Rossi et al. [21]	2016	ET	202.5 ± 36.6 ^1^	200.0 ± 37.2 ^1^	−2.5 ± 17.8 ^1^ (−12.3–7.4) ^15^ −1.2 ± 8.8 ^1^ (−5.9–3.6)% ^6,7^	124.5 ± 34.7 ^1^	114.7 ± 29.9 ^1^	−9.9 ± 19.8 ^1^ (−20.8–1.1) ^15^ −7.95 ± 15.90 ^1^ (−16.70–0.88)% ^6,7^	57.9 ± 12.9 ^1^	60.9 ± 13.0 ^1^	3.0 ± 6.8 ^1^ (−0.8–6.8) ^15^ 5.18 ± 11.74 ^1^ (−1.38–11.74)% ^6,7^	NI	NI	NI
		CT	203.6 ± 30.4 ^1^	209.8 ± 34.8 ^1^	6.24 ± 30.8 (−4.0–16.5) ^15^ 3.1 ± 15.1 (−2.0–8.2)% ^6,7^	122.5 ± 25.3 ^1^	129.7 ± 27.6 ^1^	7.1 ± 27.5 (−2.0–16.3) ^15^ 5.80 ± 22.45 (−1.63–13.32)% ^6,7^	51.9 ± 10.7 ^1^	54.8 ± 12.0 ^1^	2.9 ± 6.7 (0.6–5.0) 5.59 ± 12.91 ^15^ (1.16–9.64)% ^6,7^			
		CG	195.8 ± 34.0 ^1^	201.3 ± 34.9 ^1^	5.5 ± 33.7 (−11.9–22.8) ^15^ 2.8 ± 17.2 (−6.1–11.6)% ^6,7^	117.4 ± 26.7 ^1^	123.3 ± 24.7 ^1^	5.9 ± 24.8 (−6.8–18.7) ^15^ 5.04 ± 21.20 (−8.46–15.97)% ^6,7^	49.1 ± 7.7 ^1^	50.0 ± 8.4 ^1^	0.9 ± 5.8 (−2.0–3.9) ^15^ 1.83 ± 11.81 (−4.07–7.93)% ^6,7^			
		*p* ^4^							CT: *p =* 0.013 (pre vs. post)					
Mahdirejei et al. [14]	2015	ET	211.88 ± 34.23 ^1^	204.22 ± 30.31 ^1^	NI	123.55 ± 20.15 ^1^	111.33 ± 36.74 ^1^	−12.22 ^6^ −9.89% ^7^	38.66 ± 4.24 ^1^	41.66 ± 4.35 ^1^	NI	236 ± 162 ^1^	207.22 ± 90 ^1^	NI
		ST	185.3 ± 28.91 ^1^	190.2 ± 29.84 ^1^		108.44 ± 21.69 ^1^	117.66 ± 24.78 ^1^	NI	36.66 ± 3.84 ^1^	39.11 ± 5.32 ^1^	2.45 ^6^ 6.68% ^7^	166 ± 77.51 ^1^	151.33 ± 56.46 ^1^	−14.66 ^6^ −8.83% ^7^
		CG	182.12 ± 43.69 ^1^	190.12 ± 48.52 ^1^		106.50 ± 22.77 ^1^	113.62 ± 36.74 ^1^		38.37 ± 3.24 ^1^	37.87 ± 4.94 ^1^	NI	150 ± 47.74 ^1^	153.87 ± 63.55 ^1^	NI
		*p* ^4^				ET: *p =* 0.05 (pre vs. post)								
Huffman et al. ^b^ [59]	2014	ET ^16^	NI	NI	NI	NI	NI	NI	NI	NI	NI	147 ± 67.3 ^1,6^	124 ± 39 ^1,6^	NI
		ET ^17^										123.1 ± 48.7 ^1,6^	124 ± 65.5 ^1,6^	
		ET ^18^										139.1 ± 54.0 ^1,6^	120.5 ± 45.2 ^1,6^	
		ST										132.9 ± 63.8 ^1,6^	126.7 ± 57.6 ^1,6^	
		CT										130.2 ± 43.4 ^1,6^	112.5 ± 50.5 ^1,6^	
		CG										163.9 ± 74.4 ^1,6^	153.2 ± 83.3 ^1,6^	
		*p* ^4^										ET: *p <* 0.05 (pre vs. post)		
Sousa et al. [60]	2014	ET	194 ± 31.3 ^1^	195 ± 28.7 ^1,14^ 200 ± 15.6 ^1,19^ 196 ± 21.2 ^1,20^ 193 ± 27.3 ^1,21^	−1.0 −0.5% ^6,7^	126 ± 24.4 ^1^	120 ± 25.2 ^1,14^ 125 ± 11.9 ^1,19^ 122 ± 15.5 ^1,20^ 117 ± 19.0 ^1,21^	−8.9 −7.06% ^6,7^	52.5 ± 6.8 ^1^	52.4 ± 7.2 ^1,14^ 50.7 ± 7.8 ^1,19^ 48.4 ± 6.0 ^1,20^ 48.7 ± 6.3 ^1,21^	−3.68 ^6^ −7% ^7^	114 ± 56.6 ^1^	86.5 ± 31.6 ^1,14^ 103 ± 31.7 ^1,19^ 101 ± 26.7 ^1,20^ 104 ± 59.7 ^1,21^	−10.1 −8.86% ^6,7^
		CT	207 ± 30.7 ^1^	205 ± 40.1 ^1,14^ 195 ± 32.8 ^1,19^ 196 ± 32.7 ^1,20^ 189 ± 26.9 ^1,21^	−17.6 −8.5% ^6,7^	137 ± 30.5 ^1^	127 ± 31.8 ^1,14^ 125 ± 23.9 ^1,19^ 122 ± 23.3 ^1,20^ 120 ± 23.1 ^1,21^	−17.2 −12.56% ^6,7^	56.1 ± 13.0 ^1^	52.6 ± 12.4 ^1,14^ 52.1 ± 10.4 ^1,19^ 52.4 ± 10.5 ^1,20^ 52.0 ± 9.4 ^1,21^	−4.49 ^6^ −8% ^7^	112 ± 56.8 ^1^	101 ± 43.4 ^1,14^ 91.8 ± 32.4 ^1,19^ 96.4 ± 35.7 ^1,20^ 86.5 ± 35.7 ^1,21^	−25.8 −20.04% ^6,7^
		CG	191 ± 30.5 ^1^	196 ± 27.0 ^1,15^ 210 ± 23.5 ^1,19^ 202 ± 25.6 ^1,20^ 200 ± 25.5 ^1,21^	8.7 4.6% ^6,7^	128 ± 22.8 ^1^	120 ± 20.6 ^1,15^ 133 ± 23.0 ^1,19^ 122 ± 22.2 ^1,20^ 125 ± 22.6 ^1,21^	NI	52.5 ± 12.1 ^10^	51.0 ± 10.5 ^1,14^ 51.9 ± 12.1 ^1,19^ 53.3 ± 12.5 ^1,20^ 52.1 ± 13.3 ^1,21^	NI	93.5 ± 32.1 ^10^	117 ± 48.8 ^1,14^ 122 ± 71.3 ^1,19^ 110 ± 66.1 ^1,20^ 107 ± 54.2 ^1,21^	13.5 14.44% ^6,7^
Changela et al. [61]	2013	ET	242.70 ± 21.176 ^1^	233.00 ± 19.539 ^1^	NI	NI	NI	NI	45.40 ± 3.533 ^1^	53.60 ± 3.134 ^1^	NI	NI	NI	NI
		ST	247.50 ± 13.360 ^1^	242.60 ± 13.945 ^1^					46.10 ± 5.724 ^1^	49.40 ± 4.993 ^1^				
		*p* ^4^							ET: *p <* 0.05 (pre vs. post)					
Donges et al. [62]	2013	ET	204 ± 10 ^6,11^	NI	NI	119 ± 9 ^6,11^	NI	NI	43.33 ± 2.33 ^11^	NI	NI	177.1 ± 34.5 ^11^	NI	NI
		ST	188 ± 7 ^6,11^			113 ± 7 ^6,11^			43.0 ± 2.33 ^11^			128.4 ± 16.8 ^11^		
		CT	223 ± 12 ^6,11^			138 ± 10 b ^6,11^			46.33 ± 2.33 ^11^			149.7 ± 13.3 ^11^		
		CG	187 ± 17 ^6,11^			111 ± 15 ^6,11^			42.0 ± 4.67 ^11^			138.2 ± 27.5 ^11^		
		*p* ^4^	CT vs. ST: *p <* 0.05 (pre, post hoc)			CT vs. ST: *p <* 0.05 (pre, post hoc)								
Kadoglou et al. [63]	2013	ET	235 ± 52 ^1,6^	NI	−16 ± 5 ^1,6^ −6.7 ± 2.06% ^1,6,7^	148 ± 51 ^1,6^	NI	−11 ± 4 ^1,6^ −7.31 ± 2.61% ^1,6,7^	51 ± 12.8 ^1,6^	NI	7 ± 2.7 ^1,6^ 13.73 ± 5.29% ^1,6,7^	154.1 ± 47.8 ^1,6^	NI	−34.5 ± 8.9 ^1,6^ −22.39 ± 5.78% ^1,6,7^
		ST	224 ± 34 ^1,6^		−11 ± 4 ^1,6^ −4.8 ± 1.7% ^1,6,7^	142 ± 28^1,6^		−2 ± 0.8 ^1,6^ −1.36 ± 0.55% ^1,6,7^	49.9 ± 17 ^1,6^		−2.7 ± 1 ^1,6^ −5.41 ± 2.00% ^1,6,7^	144.4 ± 64.7 ^1,6^		−21.3 ± 5.3 ^1,6^ −14.75 ± 3.67% ^1,6,7^
		CT	2.33 ± 59 ^1,6^		−29 ± 5 ^1,6^ −12.4 ± 2.0% ^1,6,7^	142 ± 43^1,6^		−18 ± 5 ^1,6^ −12.53 ± 3.27% ^1,6,7^	48.7 ± 9.7 ^1,6^		5.8 ± 1.9 ^1,6^ 11.91 ± 3.90% ^1,6,7^	157.7 ± 75.3 ^1,6^		−43.4 ± 8 ^1,6^ −27.52 ± 5.07% ^1,6,7^
		CG	227 ± 48 ^1,6^		−5 ± 2 ^1,6^ −2.0 ± 0.9% ^1,6,7^	143 ± 45^1,6^		0.8 ± 0.4 ^1,31,6^ 0.54 ± 0.27% ^1,6,7^	53 ± 10.8 ^1,6^		−3.9 ± 1.9 ^1,6^ −7.36 ± 3.59% ^1,6,7^	147.9 ± 62.9 ^1,6^		−2.7 ± 0.9 ^1,6^ −1.83 ± 0.61% ^1,6,7^
		*p* ^4^	*p =* 0.047 (changes) ET, CT: *p <* 0.05 (pre vs. post) CT vs. CG: *p =* 0.041 (changes, post hoc)			*p =* 0.044 (changes) ET, CT: *p <* 0.05 (pre vs. post) CT vs. CG: *p =* 0.039 (changes, post hoc)			*p =* 0.046 (changes) ET: *p <* 0.05 (pre vs. post) ET vs. CG: *p =* 0.029 (changes, post hoc)			*p =* 0.003 (changes) ET, ST, CT: *p <* 0.05 (pre vs. post) CT vs. CG: *p <* 0.001, ET vs. CG: *p =* 0.004, ST vs. CG: *p =* 0.011 (changes, post hoc)		
Paoli et al. [64]	2013	ET	216.3 ± 4 ^1^	210.9 ± 4.3 ^1^	NI	120.6 ± 4.2 ^1^	116.9 ± 4.7 ^1^	NI	49.3 ± 1.6 ^1^	49.1 ± 1.8 ^1^	NI	231.7 ± 2.9 ^1^	224.2 ± 4.3 ^1^	NI
		CT ^22^	213 ± 3.7 ^1^	193 ± 2.4 ^1^		115 ± 3.8 ^1^	97 ± 3.2 ^1^		51 ± 0.6 ^1^	56 ± 1.2 ^1^		235 ± 4 ^1^	200 ± 1.7 ^1^	
		CT ^23^	227 ± 3.6 ^1^	221 ± 3.6 ^1^		117.6 ± 4.5 ^1^	114.4 ± 4.8 ^1^		50.2 ± 1.2 ^1^	51.2 ± 1.4 ^1^		235 ± 3.2 ^1^	218.8 ± 3.4 ^1^	
		*p* ^4^	ET, CT ^23^: *p <* 0.05, CT ^22^: *p <* 0.005 (pre vs. post) CT ^22^ vs. CT ^23^, ET: *p <* 0.05 (post, post hoc)			ET, CT ^22^, CT ^23^: *p <* 0.05 (pre vs. post) CT ^22^ vs. CT ^23^, ET: *p <* 0.05 (post, post hoc)			CT ^22^: *p <* 0.001 (pre vs. post) CT ^22^ vs. CT ^23^, ET: *p <* 0.05 (post, post hoc)			CT^22^: *p <* 0.001, ET, CT^23^: *p <* 0.005 (pre vs. post) CT^22^ vs. CT^23^, ET: *p <* 0.05 (post, post hoc)		
Venojärvi et al. [65]	2013	ET	205 ± 8 ^1,6^	NI	−8 ± 4 ^1,6^ −3.8 ± 1.9% ^1,6,7^	131 ± 4 ^1,6^	NI	−8 ± 4 ^1,6^ −6.11 ± 3.05% ^1,6,7^	46.4 ± 3.9 ^1,6^	NI	0.0 ± 0.0 ^1,6^ 0.0 ± 0.0% ^1,6,7^	168.3 ± 17.7 ^1,6^	NI	−26.6 ± 17.7 ^1,6^ −15.81 ± 10.52% ^1,6,7^
		ST	186 ± 8 ^1,6^		8 ± 4 ^1,6^ 4.2 ± 2.1% ^1,6,7^	112 ± 4 ^1,6^		8 ± 4 ^1,6^ 7.14 ± 3.57% ^1,6,7^	46.4 ± 3.9 ^1,6^		3.9 ± 0.0 ^1,6^ 8.41 ± 0.0% ^1,6,7^	168.3 ± 26.6 ^1,6^		0.0 ± 17.7 ^1,6^ 0.0 ± 17.7% ^1,6^
		CG	201 ± 8 ^1,6^		4 ± 4 ^1,6^ 1.9 ± 1.9% ^1,6,7^	128 ± 4 ^1,6^		4 ± 4 ^1,6^ 3.13 ± 3.13% ^1,6,7^	46.4 ± 3.9 ^1,6^		3.9 ± 0.0 ^1,6^ 8.41 ± 0.0% ^1,6,7^	141.7 ± 17.7 ^1,6^		−8.9 ± 17.7 ^1,6^ −6.28 ± 12.49% ^1,6,7^
		*p* ^4^	*p =* 0.005 (changes) ET vs. ST: *p =* 0.003 ^24^ (changes, post hoc)			*p =* 0.035 (pre) *p =* 0.012 (changes) ET vs. ST: *p =* 0.048 ^24^ (pre and changes, post hoc)								
Ho et al. [66]	2012	ET	225 ± 12 ^6^	221 ± 11 ^6,15^ 215 ± 14 ^6,25^	NI	150 ± 12 ^6^	148 ± 10 ^6,14^ 140.54 ± 13 ^6,25^	NI	53.4 ± 3.5 ^6^	50.7 ± 3.1 ^6,14^ 49.5 ± 10.4 ^6,25^	NI	120.5 ± 16.8 ^6^	114.3 ± 9.7 ^6,14^ 124 ± 14.2 ^6,25^	NI
		ST	212 ± 15 ^6^	223 ± 14 ^6,15^ 238 ± 17 ^6,25^		138 ± 13 ^6^	145 ± 12 ^6,14^ 157.53 ± 14 ^6,256^		51.8 ± 3.1 ^6^	55.7 ± 3.1 ^6,14^ 55.7 ± 3.1 ^6,25^		112.5 ± 10.6 ^6^	107.2 ± 14.2 ^6,14^ 122.2 ± 15.9 ^6,25^	
		CT	221 ± 12 ^6^	222 ± 10 ^6,15^ 222 ± 10 ^6,25^		146 ± 10 ^6^	147 ± 9 ^6,14^ 143.24 ± 8.5 ^6,25^		55.3 ± 4.3 ^6^	55.7 ± 3.9 ^6,14^ 54.5 ± 4.3 ^6,25^		97.4 ± 8.9 ^6^	95.7 ± 8.9 ^6,14^ 120.5 ± 15.1 ^6,25^	
		CG	213 ± 11 ^6^	227 ± 12 ^6,15^ 212 ± 11 ^6,25^		136 ± 10 ^6^	147 ± 12 ^6,14^ 133.9 ± 9.6 ^6,25^		1.42 ± 0.3 ^6^	156.1 ± 4.3 ^6,14^ 52.2 ± 3.9 ^6,25^		110.7 ± 15.1^6^	121.3 ± 16.8 ^6,14^ 131.1 ± 20.4 ^6,25^	
		*p* ^4^	ST: *p* <0.05 (pre vs. post) ST vs. ET, CG: *p* <0.05 (post, post hoc)			ST: *p* <0.05 (pre vs. post) ST vs. ET, CT, CG: *p* <0.05 (post, post hoc)			ET, ST: *p* <0.05 (pre vs. post) ST vs. ET: *p <* 0.05 (mild and post, post hoc) ST vs. CG: *p <* 0.05 (post, post hoc)			CT: *p* <0.05 (pre vs. post)		
Stensvold et al. ^c^ [67]	2012	ET	NI	NI	NI	NI	NI	NI	45.2 ± 13.5 ^1,6^	NI	NI	203.7 ± 88.6 ^1,6^	NI	NI
		ST							44.5 ± 7 ^1,6^			159.4 ± 79.7 ^1,6^		
		CG							49.9 ± 13.5 ^1,6^			150.6 ± 70.9 ^1,6^		
Sukala et al. [68]	2012	ET	174 ± 15 ^1,6^	182 ± 15 ^1,6^	12 ± 23 ^1,6^ 6.7 ± 13.3% ^1,6,7^	101 ± 23 ^1,6^	104 ± 15 ^1,6^	4 ± 19 ^1,6^ 3.96 ± 18.81% ^1,6,7^	42.5 ± 7.7 ^1,6^	42.5 ± 7.7 ^1,6^	0.0 ± 3.9 ^1,6^	141.7 ± 44.3 ^1,6^	168.3 ± 53.2 ^1,6^	26.6 ± 17.7 ^1,6^ 18.77 ± 12.49% ^1,6,7^
		ST	189 ± 58 ^1,6^	174 ± 39 ^1,6^	−15 ± 35 ^1,6^ −8.2 ± 18.4% ^1,6,7^	104 ± 54 ^1,6^	93 ± 27 ^1,6^	−12 ± 3 1^1,6^ −11.54 ± 29.81% ^1,6,7^	50.3 ± 15.5 ^1,6^	50.3 ± 19.3 ^1,6^	0.0 ± 3.9 ^1,6^	194.9 ± 106.3 ^1,6^	177.1 ± 88.6 ^1,6^	−17.7 ± 53.1 ^1,6^ −9.08 ± 27.24% ^1,6,7^
		*p* ^4^	ET vs. ST: *p =* 0.08 (changes, post hoc)									ET: *p =* 0.004 (pre vs. post) ET vs. ST: *p =* 0.03 (changes, post hoc)		
Bateman et al. ^b^ [13]	2011	ET	NI	NI	NI	NI	NI	NI	41.5 ± 14.2 ^1^	NI	1.03 ± 4.81 ^1^ 2.48 ± 11.59% ^1,6,7^	154 ± 81.3 ^1^	NI	−21 ± 56 ^1^ −13.64 ± 36.36% ^1,6,7^
		ST							46.8 ± 13.9 ^1^		−0.63 ± 4.81 ^1^ −1.35 ± 10.28% ^1,6,7^	140 ± 81.0 ^1^		−5.25 ± 52.6 ^1^ −3.75 ± 37.57% ^1,6,7^
		CT							45.0 ± 11.0 ^1^		1.55 ± 5.84 ^1^ 3.44 ± 12.98% ^1,6,7^	152 ± 93.9 ^1^		−30.1 ± 49.8 ^1^ −19.80 ± 32.76% ^1,6,7^
		*p* ^4^										ET: *p =* 0.049, CT: *p =* 0.006 (pre vs. post) CT vs. ST: *p <* 0.10 (changes, post hoc)		
Jorge et al. [69]	2011	ET	183.13 ± 23.09 ^1^	165.75 ± 31.38 ^1^	NI	103.2 ± 22.16 ^1^	NI	NI	47.15 ± 9.54 ^1^	44.11 ± 7.74 ^1^	NI	141.88 ± 47.63 ^1^	127.63 ± 55.22 ^1^	NI
		ST	164.38 ± 30.10 ^1^	153.00 ± 25.56 ^1^		88.5 ± 28.85 ^1^			39.38 ± 7.78 ^1^	34.75 ± 3.62 ^1^		236.38 ± 231.37 ^1^	154.63 ± 76.44 ^1^	
		CT	181.13 ± 29.23 ^1^	178.75 ± 30.27 ^1^		99.11 ± 21.03 ^1^			46.13 ± 7.97 ^1^	46.50 ± 7.58 ^1^		157.88 ± 86.62 ^1^	131.75 ± 68.72 ^1^	
		CG	179.45 ± 33.76 ^1^	167.91 ± 35.76 ^1^		93.58 ± 36.88 ^1^			44 ± 8.20 ^1^	41.89 ± 7.65 ^1^		208.36 ± 76.63 ^1^	157.09 ± 64.46 ^1^	
		*p* ^4^	*p <* 0.05 (changes)						ET, ST, CG: *p <* 0.05 (pre vs. post)			*p <* 0.05 (changes)		
Gram et al. [70]	2010	ET	166.5 ± 34.2 ^1,6^	173.1 ± 7.7 ^6,19,26^ 153.8 ± 7.7 ^6,27,27^	NI	98.5 ± 32.7 ^1,6^	100 ± 7.7 ^6,19,26^ 69.2 ± 7.7 ^6,26,27^	NI	44.2 ± 11.2 ^1,6^	46.2 ± 3.9 ^6,20,27^ 42.3 ± 3.9 ^6,26,28^	NI	170.1 ± 113.4 ^1,6^	NI	NI
		CT	168.8 ± 29.2 ^1,6^	180.8 ± 7.7 ^6,19,26^ 176.9 ± 7.7 ^6,26,27^		98.9 ± 23.5 ^1,6^	115.4 ± 7.7 ^6,19,26^ 100 ± 15.4 ^6,26,27^		44.6 ± 16.5 ^1,6^	42.3 ± 3.9 ^6,20,27^ 103.9 ± 7.7 ^6,27,28^		181.6 ± 134.6 ^1,6^		
		CG	176.5 ± 53.5 ^1,6^	173.1 ± 7.7 ^6,19,26^ 169.2 ± 7.7 ^6,26,27^		98.5 ± 25.7 ^1,6^	96.2 ± 3.9 ^6,19,26^ 100 ± 19.2 ^6,26,27^		42.3 ± 14.2 ^1,6^	42.3 ± 3.9 ^6,20,26^ 92.3 ± 7.7 ^6,26,27^		236.5 ± 296.3 ^1,6^		
Stensvold et al. ^c^ [22]	2010	ET	236 ± 37 ^1,6^	218 ± 24 ^1,6^	−13 (−27–1) ^6,28^ −5.6 (−11.5–0.3)% ^6,7,28^	NI	NI	NI	45.2 ± 13.5 ^1,6,19^	47.6 ± 15.5 ^1,6,19^	2.3 (−4.3–8.9) ^6,28^ 5.09 (−9.51–19.70)% ^6,7,28^	203.7 ± 88.6 ^1,6^	159.4 ± 70.9 ^1,6^	−35.4 (−79.7–17.7) ^6,29^ −17.38 (−39.12- 8.69)% ^6,7,28^
		ST	212 ± 47 ^1,6^	223 ± 39 ^1,6^	5 (−10–20) ^6,28^ 2.4 (−4.6–9.4)% ^6,7,28^				44.5 ± 7 ^1,6^	47.6 ± 8.1 ^1,6^	3.1 (−3.9–10.1) ^6,28^ 6.97 (−8.76–22.71)% ^6,7,28^	159.4 ± 79.7 ^1,6^	168.3 ± 106.3 ^1,6^	−8.9 (−53.1–44.3) ^6,29^ −5.58 (−33.31–27.77)% ^6,7,28^
		CT	244 ± 34 ^1,6^	229 ± 26 ^1,6^	−13 (−29–3) ^6,28^ −5.2 (−11.9–1.4)% ^6,7,28^				53.8 ± 14.7 ^1,6^	57.6 ± 24.7 ^1,6^	4.6 (−3.5–12.4) ^6,28^ 8.55 (−6.51–23.05)% ^6,7,28^	230.3 ± 124 ^1,6^	203.7 ±97.4 ^1,6^	−8.9 (−70.9–44.3) ^6,29^ −3.87 (−30.79–19.26)% ^6,7,28^
		CG	221 ± 49 ^1,6^	216 ± 37 ^1,6^	−7 (−21–8) ^6,29^ −3.1 (−9.6–3.4)% ^6,7,28^				49.9 ± 13.5 ^1,6^	49.1 ± 24.4 ^1,6^	−0.8 (−7.3–6.2) ^6,28^ −1.60 (−14.63–12.4)% ^6,7,28^	150.6 ± 70.9 ^1,6^	150.6 ± 88.6 ^1,6^	−8.9 (−53.1–44.3) ^6,29^ −5.91 (−35.26–29.42)% ^6,7,28^
Hara et al. [72]	2005	ET	164.7 ± 31.5 ^1^	172.9 ± 36.9 ^1^	NI	NI	NI	NI	39.6 ± 6.6 ^1^	42.0 ± 6.7 ^1^	NI	136.7 ± 40.1 ^1^	138.9 ± 43.7 ^1^	NI
		CT	153.4 ± 23.2 ^1^	165.8 ± 14.7 ^1^					39.9 ± 5.0 ^1^	53.9 ± 8.9 ^1^		99.6 ± 46.8 ^1^	90.7 ± 24.2 ^1^	
		CG	170.0 ± 41.3 ^1^	175.4 ± 37.7 ^1^					38.9 ± 9.7 ^1^	42.3 ± 8.6 ^1^		119.9 ± 74.8 ^1^	126.3 ± 59.7 ^1^	
		*p* ^4^	CT: *p <* 0.05 (pre vs. post)						CT: *p <* 0.01 (pre vs. post)					
Banz et al. [73]	2003	ET	205.0 ± 44.3 ^1^	209.1 ± 42.3 ^1^	NI	129.8 ± 56.7 ^1^	133.2 ± 39.5 ^1^	NI	29.8 ± 7.0 ^1^	33.7 ± 4.0 ^1^	NI	NI	NI	NI
		ST	203.0 ± 41.6 ^1^	205.6 ± 43.6 ^1^		93.0 ± 70.4 ^1^	114.3 ± 63.2 ^1^		31.7 ± 8.4 ^1^	32.0 ± 7.8 ^1^				
		*p* ^4^							*p <* 0.05 (pre vs. post)					

TC—total cholesterol; LDL-C—low-density lipoprotein cholesterol; HDL-C—high-density lipoprotein cholesterol; TG—triglyceride; CG—control group; CT—combined training; ET—endurance training; ST—strength training; NI—no information. ^1^ Mean ± standard deviation; ^2^ Adjusted values; ^3^ Mean and 95% confidence intervals; ^4^ Only statistically significant values are shown; ^5^ Data from figure; ^6^ Converted values; ^7^ Relative changes; ^8^ Moderate-intensity continuous training group; ^9^ High-intensity interval training group; ^10^ Least square means (means adjusted for baseline) with (95% confidence intervals); ^11^ Means ± standard error; ^12^ Data shown as log; ^13^ 4th week of intervention; ^14^ 8th week of intervention; ^15^ No information about data format; ^16^ Low-amount moderate-intensity training group; ^17^ Low-amount vigorous-intensity training group; ^18^ High-amount vigorous-intensity training group; ^19^ 16th week of intervention; ^20^ 24th week of intervention; ^21^ 32nd week of intervention; ^22^ High-intensity circuit training; ^23^ Low-intensity circuit training; ^24^ Bonferroni correction; ^25^ 12th week of intervention; ^26^ Least squares means ± standard error; ^27^ 52nd week of intervention; ^28^ Estimated margins of the mean (95% confidence intervals); ^a–c^ Studies marked with the same letters were conducted in the same population.

## Data Availability

Template data collection forms, data extracted from included studies, data used for analysis, analytic code, and any other materials used in the review are available on reasonable request from the corresponding author (J.W.).

## Supplementary Materials

The following supporting information can be downloaded at: https://www.mdpi.com/article/10.3390/ijerph192214928/s1, Figure S1: Funnel plot of standard error by standard differences in means of glucose levels: (A) endurance vs. strength training (Begg-Mazumdar test: Kendall’s tau = −0.152, *p =* 0.346, Egger test: bias = (−2.780% confidence interval: −6.578; 1.017), *p =* 0.070); (B) endurance vs. endurance-strength training (Begg-Mazumdar test: Kendall’s tau = 0.323, *p =* 0.092, Egger test: bias = 3.168 (95% confidence interval: −0.582; 6.691), *p =* 0.091); (C) strength vs. endurance-strength training (Begg-Mazumdar test: Kendall’s tau = 0.472, *p =* 0.076, Egger test: bias = 7.420 (95% confidence interval: 0.725; 14.079), *p =* 0.034). Std diff—standard differences; Figure S2: Funnel plot of standard error by standard differences in means of insulin levels: (A) endurance vs. strength training (Begg-Mazumdar test: Kendall’s tau = 0.285, *p =* 0.137, Egger test: bias = 4.128 (95% confidence interval: 0.804; 7.453), *p =* 0.018); (B) endurance vs. endurance-strength training (Begg-Mazumdar test: Kendall’s tau = 0.166, *p =* 0.450, Egger test: bias = 1.046 (95% confidence interval: −3.121; 5.214), *p =* 0.588); (C) strength vs. endurance-strength training (Begg-Mazumdar test: Kendall’s tau = 0.000, *p =* 1.000, Egger test: bias = −4.798 (95% confidence interval: −14.012; 4.416), *p =* 0.238). Std diff—standard differences; Figure S3: Funnel plot of standard error by standard differences in means of glycated haemoglobin levels: (A) endurance vs. strength training (Begg-Mazumdar test: Kendall’s tau = −0.821, *p =* 0.004, Egger test: bias = −6.250 (95% confidence interval: −14.302; 1.803), *p =* 0.106); (B) endurance vs. endurance-strength training (Begg-Mazumdar test: Kendall’s tau = 0.222, *p =* 0.371, Egger test: bias = 0.648 (95% confidence interval: −3.142; 4.438), *p =* 0.704); (C) strength vs. endurance-strength training (Begg-Mazumdar test: Kendall’s tau = −0.166, *p =* 0.734, Egger test: bias = 3.034 (95% confidence interval: −51.298; 57.365), *p =* 0.832). Std diff—standard differences; Figure S4: Funnel plot of standard error by standard differences in means of homeostatic model assessment of insulin resistance index: (A) endurance vs. strength training (Begg-Mazumdar test: Kendall’s tau = −0.007, *p =* 0.967, Egger test: bias = −1.536 (95% confidence interval: −5.535; 2.462), *p =* 0.425); (B) endurance vs. endurance-strength training (Begg-Mazumdar test: Kendall’s tau = 0.285, *p =* 0.154, Egger test: bias = 2.151 (95% confidence interval: −0.386; 4.689), *p =* 0.089); (C) strength vs. endurance-strength training (Begg-Mazumdar test: Kendall’s tau = 0.638, *p =* 0.016, Egger test: bias = 7.287 (95% confidence interval: 0.440; 14.134), *p =* 0.040). Std diff—standard differences; Figure S5: Funnel plot of standard error by standard differences in means of total cholesterol levels: (A) endurance vs. strength training (Begg-Mazumdar test: Kendall’s tau = 0.025, *p =* 0.892, Egger test: bias = 0.179 (95% confidence interval: −10.916; 11.276), *p =* 0.972); (B) endurance vs. endurance-strength training (Begg-Mazumdar test: Kendall’s tau = 0.143, *p =* 0.404, Egger test: bias = 1.503 (95% confidence interval: −3.059; 6.066), *p =* 0.494); (C) strength vs. endurance-strength training (Begg-Mazumdar test: Kendall’s tau = 0.285, *p =* 0.367, Egger test: bias = 10.660 (95% confidence interval: −10.815; 32.136), *p =* 0.258). Std diff—standard differences; Figure S6: Funnel plot of standard error by standard differences in means of low-density lipoprotein cholesterol levels: (A) endurance vs. strength training (Begg-Mazumdar test: Kendall’s tau = −0.269, *p =* 0.200, Egger test: bias = −5.756 (95% confidence interval: −18.840; 7.327), *p =* 0.356); (B) endurance vs. endurance-strength training (Begg-Mazumdar test: Kendall’s tau = −0.419, *p =* 0.029, Egger test: bias = −4.473 (95% confidence interval: −11.154; 2.206), *p =* 0.171); (C) strength vs. endurance-strength training (Begg-Mazumdar test: Kendall’s tau = 0.500, *p =* 0.220, Egger test: bias = 12.691 (95% confidence interval: −9.612; 34.995), *p =* 0.167). Std diff—standard differences; Figure S7: Funnel plot of standard error by standard differences in means of high-density lipoprotein cholesterol levels: (A) endurance vs. strength training (Begg-Mazumdar test: Kendall’s tau = 0.257, *p =* 0.149, Egger test: bias = 4.905 (95% confidence interval: −1.554; 11.365), *p =* 0.126); (B) endurance vs. endurance-strength training (Begg-Mazumdar test: Kendall’s tau = −0.078, *p =* 0.626, Egger test: bias = −0.138 (95% confidence interval: −4.696; 4.418), *p =* 0.949); (C) strength vs. endurance-strength training (Begg-Mazumdar test: Kendall’s tau = −0. 535, *p =* 0.063, Egger test: bias = −8. 209 (95% confidence interval: −17.585; 1.166), *p =* 0.075). Std diff—standard differences; Figure S8: Funnel plot of standard error by standard differences in means of triglycerides levels: (A) endurance vs. strength training (Begg-Mazumdar test: Kendall’s tau = −0.041, *p =* 0.821, Egger test: bias = 0.621 (95% confidence interval: −4.519; 5.763), *p =* 0.799); (B) endurance vs. endurance-strength training (Begg-Mazumdar test: Kendall’s tau = −0.209, *p =* 0.225, Egger test: bias = −0.089 (95% confidence interval: −3.932; 3.753), *p =* 0.961); (C) strength vs. endurance-strength training (Begg-Mazumdar test: Kendall’s tau = 0.321, *p =* 0.265, Egger test: bias = 4.997 (95% confidence interval: −5.087; 15.082), *p =* 0.270). Std diff—standard differences; Figure S9: Traffic-light plot of the risk of bias [13,14,15,16,20,21,22,38,39,40,41,42,43,44,45,46,47,48,49,50,51,52,53,54,55,56,57,58,59,60,61,62,63,64,65,66,67,68,69,70,71,72,73,74,75]; Figure S10: Summary plot of the risk of bias; Figure S11: Sensitivity analysis by the jack-knife approach presenting mean differences with 95% confidence interval in glucose levels between different training programmes: (A) endurance (favours A) vs. strength (favours B) training (random model) [16,20,41,43,44,45,46,49,50,51,53,58,63,65,66,67,71,73]; (B) endurance (favours A) vs. endurance-strength (favours B) training (random model) [20,22,37,38,39,41,46,48,51,54,58,63,66,69,72]; (C) strength (favours A) vs. endurance-strength (favours B) training (random model) [20,22,41,46,51,58,63,66,69]. CI—confidence interval; Std diff—standard differences; Figure S12: Sensitivity analysis by the jack-knife approach presenting mean differences with 95% confidence interval in insulin levels between different training programmes: (A) endurance (favours A) vs. strength (favours B) training (random model) [16,20,41,44,45,46,50,51,58,63,65,66,68,71,73]; (B) endurance (favours A) vs. endurance-strength (favours B) training (random model) [20,38,39,41,42,46,48,51,58,63,66,72]; (C) strength (favours A) vs. endurance-strength (favours B) training (random model) [20,41,46,51,58,63,66]. CI—confidence interval; Std diff—standard differences; Figure S13: Sensitivity analysis by the jack-knife approach presenting mean differences with 95% confidence interval in glycated haemoglobin levels between different training programmes: (A) endurance (favours A) vs. strength (favours B) training (random model) [22,44,45,46,63,65,68,69]; (B) endurance (favours A) vs. endurance-strength (favours B) training (random model) [22,37,38,39,46,48,63,69,70,74]; (C) strength (favours A) vs. endurance-strength (favours B) training (random model) [22,46,63,69]. CI—confidence interval; Std diff—standard differences; Figure S14: Sensitivity analysis by the jack-knife approach presenting mean differences with 95% confidence interval in the homeostatic model assessment of insulin resistance index between different training programmes: (A) endurance (favours A) vs. strength (favours B) training (random model) [16,20,22,41,43,45,46,50,51,53,55,58,63,65,68,69,71]; (B) endurance (favours A) vs. endurance-strength (favours B) training (random model) [20,22,37,38,39,41,46,48,51,55,58,63,69,72]; (C) strength (favours A) vs. endurance-strength (favours B) training (random model) [20,22,41,46,51,55,58,63,69]. CI—confidence interval; Std diff—standard differences; Figure S15: Sensitivity analysis by the jack-knife approach presenting mean differences with 95% confidence interval in 2 h glucose levels between endurance (favours A) vs. strength (favours B) training programmes (fixed model) [62,65]. CI—confidence interval; Std diff—standard differences; Figure S16: Sensitivity analysis by the jack-knife approach presenting mean differences with 95% confidence interval in 2 h insulin levels between endurance (favours A) vs. strength (favours B) training programmes (fixed model) [62,65]. CI—confidence interval; Std diff—standard differences; Figure S17: Sensitivity analysis by the jack-knife approach presenting mean differences with 95% confidence interval in C-peptide levels between endurance (favours A) vs. strength (favours B) training programmes (fixed model) [22,68]. CI—confidence interval; Std diff—standard differences; Figure S18: Sensitivity analysis by the jack-knife approach presenting mean differences with 95% confidence interval in total cholesterol levels between different training programmes: (A) endurance (favours A) vs. strength (favours B) training (random model) [14,22,41,43,44,45,46,55,57,61,63,65,66,68,69,73]; (B) endurance (favours A) vs. endurance-strength (favours B) training (random model) [21,22,37,38,41,46,47,52,54,55,56,60,63,64,66,69,70,72]; (C) strength (favours A) vs. endurance-strength (favours B) training (random model) [22,41,46,55,63,66,69]. CI—confidence interval; Std diff—standard differences; Figure S19: Sensitivity analysis by the jack-knife approach presenting mean differences with 95% confidence interval in low-density lipoprotein cholesterol levels between different training programmes: (A) endurance (favours A) vs. strength (favours B) training (random model) [14,41,43,44,45,46,55,57,63,65,66,68,73]; (B) endurance (favours A) vs. endurance-strength (favours B) training (random model) [21,37,38,41,46,47,52,54,55,56,60,63,64,66,70]; (C) strength (favours A) vs. endurance-strength (favours B) training (random model) [41,46,55,63,66]. CI—confidence interval; Std diff—standard differences; Figure S20: Sensitivity analysis by the jack-knife approach presenting mean differences with 95% confidence interval in high-density lipoprotein cholesterol levels between different training programmes: (A) endurance (favours A) vs. strength (favours B) training (random model) [13,14,22,41,43,44,45,46,49,55,57,61,63,66,68,69,73]; (B) endurance (favours A) vs. endurance-strength (favours B) training (random model) [13,21,22,37,38,39,41,46,47,52,54,55,56,60,63,64,66,69,70,72]; (C) strength (favours A) vs. endurance-strength (favours B) training (random model) [13,22,41,46,55,63,66,69]. CI—confidence interval; Std diff—standard differences; Figure S21: Sensitivity analysis by the jack-knife approach presenting mean differences with 95% confidence interval in triglycerides levels between different training programmes: (A) endurance (favours A) vs. strength (favours B) training (random model) [13,14,22,41,43,45,46,49,53,55,57,63,65,66,68,69]; (B) endurance (favours A) vs. endurance-strength (favours B) training (random model) [13,22,37,38,39,41,46,47,52,54,55,56,60,63,64,66,69,72]; (C) strength (favours A) vs. endurance-strength (favours B) training (random model) [13,22,41,46,55,63,66,69]. CI—confidence interval; Std diff—standard differences; Figure S22: Sensitivity analysis presenting mean differences with 95% confidence interval in glucose levels between different training programmes after exclusion of studies with an overall high risk of bias: (A) endurance (favours A) vs. strength (favours B) training (random model) [20,41,43,44,46,49,50,63,66,67]; (B) endurance (favours A) vs. endurance-strength (favours B) training (random model) [20,22,38,39,41,46,48,63,66,72]; (C) strength (favours A) vs. endurance-strength (favours B) training (random model) [20,22,41,46,63,66]. CI—confidence interval; CT—combined training; ET—endurance training; ST—strength training; Std—standard; Std diff—standard differences; Figure S23: Sensitivity analysis presenting mean differences with 95% confidence interval in insulin levels between different training programmes after exclusion of studies with an overall high risk of bias: (A) endurance (favours A) vs. strength (favours B) training (random model) [20,41,44,46,50,63,66,68]; (B) endurance (favours A) vs. endurance-strength (favours B) training (random model) [20,38,39,41,42,46,48,63,66,72]; (C) strength (favours A) vs. endurance-strength (favours B) training (random model) [20,41,46,63,66]. CI—confidence interval; CT—combined training; ET—endurance training; ST—strength training; Std—standard; Std diff—standard differences; Figure S24: Sensitivity analysis presenting mean differences with 95% confidence interval in glycated haemoglobin levels between different training programmes after exclusion of studies with an overall high risk of bias: (A) endurance (favours A) vs. strength (favours B) training (random model) [22,44,46,63,68]; (B) endurance (favours A) vs. endurance-strength (favours B) training (random model) [22,38,39,46,48,63,70]; (C) strength (favours A) vs. endurance-strength (favours B) training (random model) [22,46,63]. CI—confidence interval; CT—combined training; ET—endurance training; ST—strength training; Std—standard; Std diff—standard differences; Figure S25: Sensitivity analysis presenting mean differences with 95% confidence interval in homeostatic model assessment of insulin resistance index between different training programmes after exclusion of studies with an overall high risk of bias: (A) endurance (favours A) vs. strength (favours B) training (fixed model) [20,22,41,43,46,50,63,68]; (B) endurance (favours A) vs. endurance-strength (favours B) training (fixed model) [20,22,38,39,41,46,48,63,72]; (C) strength (favours A) vs. endurance-strength (favours B) training (random model) [20,22,41,46,63]. CI—confidence interval; CT—combined training; ET—endurance training; ST—strength training; Std—standard; Std diff—standard differences; Figure S26: Sensitivity analysis presenting mean differences with 95% confidence interval in total cholesterol levels between different training programmes after exclusion of studies with an overall high risk of bias: (A) endurance (favours A) vs. strength (favours B) training (random model) [14,22,41,43,44,46,63,66,68]; (B) endurance (favours A) vs. endurance-strength (favours B) training (random model) [22,38,41,46,47,60,63,66,70,72]; (C) strength (favours A) vs. endurance-strength (favours B) training (random model) [22,41,46,63,66]. CI—confidence interval; CT—combined training; ET—endurance training; ST—strength training; Std—standard; Std diff—standard differences; Figure S27: Sensitivity analysis presenting mean differences with 95% confidence interval in low-density lipoprotein cholesterol levels between different training programmes after exclusion of studies with an overall high risk of bias: (A) endurance (favours A) vs. strength (favours B) training (random model) [14,41,43,44,46,63,66,68]; (B) endurance (favours A) vs. endurance-strength (favours B) training (random model) [38,41,46,47,60,63,66,70]; (C) strength (favours A) vs. endurance-strength (favours B) training (random model) [41,46,63,66]. CI—confidence interval; CT—combined training; ET—endurance training; ST—strength training; Std—standard; Std diff—standard differences; Figure S28: Sensitivity analysis presenting mean differences with 95% confidence interval in high-density lipoprotein cholesterol levels between different training programmes after exclusion of studies with an overall high risk of bias: (A) endurance (favours A) vs. strength (favours B) training (random model) [13,14,22,41,43,44,46,49,63,66,68]; (B) endurance (favours A) vs. endurance-strength (favours B) training (random model) [13,22,38,39,41,46,47,60,63,66,70,72]; (C) strength (favours A) vs. endurance-strength (favours B) training (random model) [13,22,41,46,63,66]. CI—confidence interval; CT—combined training; ET—endurance training; ST—strength training; Std—standard; Std diff—standard differences; Figure S29: Sensitivity analysis presenting mean differences with 95% confidence interval in triglycerides levels between different training programmes after exclusion of studies with an overall high risk of bias: (A) endurance (favours A) vs. strength (favours B) training (random model) [13,14,22,41,43,46,49,63,66,68]; (B) endurance (favours A) vs. endurance-strength (favours B) training (random model) [13,22,38,39,41,46,47,60,63,66,72]; (C) strength (favours A) vs. endurance-strength (favours B) training (random model) [13,22,41,46,63,66]. CI—confidence interval; CT—combined training; ET—endurance training; ST—strength training; Std—standard; Std diff—standard differences; Figure S30: Cumulative meta-analysis of the effect of training programmes on glucose levels: (A) endurance (favours A) vs. strength (favours B) training [16,20,41,43,44,45,46,49,50,51,53,58,63,65,66,67,71,73]; (B) endurance (favours A) vs. endurance-strength (favours B) training [20,22,37,38,39,41,46,48,51,54,58,63,66,69,72]; (C) strength (favours A) vs. endurance-strength (favours B) training [20,22,41,46,51,58,63,66,69]. CI—confidence interval; CT—combined training; ET—endurance training; ST—strength training; Std—standard; Std diff—standard differences; Figure S31: Cumulative meta-analysis of the effect of training programmes on insulin levels: (A) endurance (favours A) vs. strength (favours B) training [16,20,41,44,45,46,50,51,58,63,65,66,68,71,73]; (B) endurance (favours A) vs. endurance-strength (favours B) training [20,38,39,41,42,46,48,51,58,63,66,72]; (C) strength (favours A) vs. endurance-strength (favours B) training [20,41,46,51,58,63,66]. CI—confidence interval; CT—combined training; ET—endurance training; ST—strength training; Std—standard; Std diff—standard differences; Figure S32: Cumulative meta-analysis of the effect of training programmes on glycated haemoglobin levels: (A) endurance (favours A) vs. strength (favours B) training [22,44,45,46,63,65,68,69]; (B) endurance (favours A) vs. endurance-strength (favours B) training [22,37,38,39,46,48,63,69,70,74]; (C) strength (favours A) vs. endurance-strength (favours B) training [22,46,63,69]. CI—confidence interval; CT—combined training; ET—endurance training; ST—strength training; Std—standard; Std diff—standard differences; Figure S33: Cumulative meta-analysis of the effect of training programmes on homeostatic model assessment of insulin resistance index: (A) endurance (favours A) vs. strength (favours B) training [16,20,22,41,43,45,46,50,51,53,55,58,63,65,68,69,71]; (B) endurance (favours A) vs. endurance-strength (favours B) training [20,22,37,38,39,41,46,48,51,55,58,63,69,72]; (C) strength (favours A) vs. endurance-strength (favours B) training [20,22,41,46,51,55,58,63,69]. CI—confidence interval; CT—combined training; ET—endurance training; ST—strength training; Std—standard; Std diff—standard differences; Figure S34: Cumulative meta-analysis of the effect of endurance (favours A) vs. strength (favours B) training programmes on 2 h glucose levels [62,65]. CI—confidence interval; ET—endurance training; ST—strength training; Std—standard; Std diff—standard differences.; Figure S35: Cumulative meta-analysis of the effect of endurance (favours A) vs. strength (favours A) training programmes on 2 h insulin levels [62,65]; Figure S36: Cumulative meta-analysis of the effect of endurance (favours A) vs. strength (favours B) training programmes on C-peptide levels [22,68]. CI—confidence interval; ET—endurance training; ST—strength training; Std—standard; Std diff—standard differences; Figure S37: Cumulative meta-analysis of the effect of training programmes on total cholesterol levels: (A) endurance (favours A) vs. strength (favours B) training [14,22,41,43,44,45,46,55,57,61,63,65,66,68,69,73]; (B) endurance (favours A) vs. endurance-strength (favours B) training [21,22,37,38,41,46,47,52,54,55,56,60,63,64,66,69,70,72]; (C) strength (favours A) vs. endurance-strength (favours B) training [22,41,46,55,63,66,69]. CI—confidence interval; CT—combined training; ET—endurance training; ST—strength training; Std—standard; Std diff—standard differences; Figure S38: Cumulative meta-analysis of the effect of training programmes on low-density lipoprotein cholesterol levels: (A) endurance (favours A) vs. strength (favours B) training [14,41,43,44,45,46,55,57,63,65,66,68,73]; (B) endurance (favours A) vs. endurance-strength (favours B) training [21,37,38,41,46,47,52,54,55,56,60,63,64,66,70]; (C) strength (favours A) vs. endurance-strength (favours B) training [41,46,55,63,66]. CI—confidence interval; CT—combined training; ET—endurance training; ST—strength training; Std—standard; Std diff—standard differences; Figure S39: Cumulative meta-analysis of the effect of training programmes on high-density lipoprotein cholesterol levels: (A) endurance (favours A) vs. strength (favours B) training [13,14,22,41,43,44,45,46,49,55,57,61,63,66,68,69,73]; (B) endurance (favours A) vs. endurance-strength (favours B) training [13,21,22,37,38,39,41,46,47,52,54,55,56,60,63,64,66,69,70,72]; (C) strength (favours A) vs. endurance-strength (favours B) training [13,22,41,46,55,63,66,69]. CI—confidence interval; CT—combined training; ET—endurance training; ST—strength training; Std—standard; Std diff—standard differences; Figure S40. Cumulative meta-analysis of the effect of training programmes on triglycerides levels: (A) endurance (favours A) vs. strength (favours B) training [13,14,22,41,43,45,46,49,53,55,57,63,65,66,68,69]; (B) endurance (favours A) vs. endurance-strength (favours B) training [13,22,37,38,39,41,46,47,52,54,55,56,60,63,64,66,69,72]; (C) strength vs. endurance-strength training [13,22,41,46,55,63,66,69]. CI—confidence interval; CT—combined training; ET—endurance training; ST—strength training; Std—standard; Std diff—standard differences; Figure S41: Subgroup meta-analysis according to time of the intervention (short (≤12 weeks) vs. long (>12 weeks)) of the effect of training programmes on glucose levels: (A) endurance (favours A) vs. strength (favours B) training [16,20,41,43,44,45,46,49,50,51,53,58,63,65,66,67,71,73]; (B) endurance (favours A) vs. endurance-strength (favours B) training [20,22,37,38,39,41,46,48,51,54,58,63,66,69,72]; (C) strength (favours A) vs. endurance-strength (favours B) training [20,22,41,46,51,58,63,66,69]. CI—confidence interval; CT—combined training; ET—endurance training; ST—strength training; Std—standard; Std diff—standard differences; Figure S42: Subgroup meta-analysis according to time of the intervention (short (≤12 weeks) vs. long (>12 weeks)) of the effect of training programmes on insulin levels: (A) endurance (favours A) vs. strength (favours B) training [16,20,41,44,45,46,50,51,58,63,65,66,68,71,73]; (B) endurance (favours A) vs. endurance-strength (favours B) training [20,38,39,41,42,46,48,51,58,63,66,72]; (C) strength (favours A) vs. endurance-strength (favours B) training [20,41,46,51,58,63,66]. CI—confidence interval; CT—combined training; ET—endurance training; ST—strength training; Std—standard; Std diff—standard differences; Figure S43: Subgroup meta-analysis according to time of the intervention (short (≤ 12 weeks) vs. long (>12 weeks)) of the effect of training programmes on glycated haemoglobin levels: (A) endurance (favours A) vs. strength (favours B) training [22,44,45,46,63,65,68,69]; (B) endurance (favours A) vs. endurance-strength (favours B) training [22,37,38,39,46,48,63,69,70,74]. CI—confidence interval; CT—combined training; ET—endurance training; ST—strength training; Std—standard; Std diff—standard differences; Figure S44: Subgroup meta-analysis according to time of the intervention (short (≤12 weeks) vs. long (>12 weeks)) of the effect of training programmes on homeostatic model assessment of insulin resistance index: (A) endurance (favours A) vs. strength (favours B) training [16,20,22,41,43,45,46,50,51,53,55,58,63,65,68,69,71]; (B) endurance (favours A) vs. endurance-strength (favours B) training [20,22,37,38,39,41,46,48,51,55,58,63,69,72]; (C) strength (favours A) vs. endurance-strength (favours B) training [20,22,41,46,51,55,58,63,69]. CI—confidence interval; CT—combined training; ET—endurance training; ST—strength training; Std—standard; Std diff—standard differences; Figure S45: Subgroup meta-analysis according to time of the intervention (short (≤12 weeks) vs. long (>12 weeks)) of the effect of training programmes on total cholesterol levels: (A) endurance (favours A) vs. strength (favours B) training [14,22,41,43,44,45,46,55,57,61,63,65,66,68,69,73]; (B) endurance (favours A) vs. endurance-strength (favours B) training [21,22,37,38,41,46,47,52,54,55,56,60,63,64,66,69,70,72]; (C) strength (favours A) vs. endurance-strength (favours B) training [22,41,46,55,63,66,69]. CI—confidence interval; CT—combined training; ET—endurance training; ST—strength training; Std—standard; Std diff—standard differences.; Figure S46: Subgroup meta-analysis according to time of the intervention (short (≤12 weeks) vs. long (>12 weeks)) of the effect of training programmes on low-density lipoprotein cholesterol levels: (A) endurance (favours A) vs. strength (favours B) training [14,41,43,44,45,46,55,57,63,65,66,68,73]; (B) endurance (favours A) vs. endurance-strength (favours B) training [21,37,38,41,46,47,52,54,55,56,60,63,64,66,70]; (C) strength (favours A) vs. endurance-strength (favours B) training [41,46,55,63,66]. CI—confidence interval; CT—combined training; ET—endurance training; ST—strength training; Std—standard; Std diff—standard differences; Figure S47: Subgroup meta-analysis according to time of the intervention (short (≤12 weeks) vs. long (>12 weeks)) of the effect of training programmes on high-density lipoprotein cholesterol levels: (A) endurance (favours A) vs. strength (favours B) training [13,14,22,41,43,44,45,46,49,55,57,61,63,66,68,69,73]; (B) endurance (favours A) vs. endurance-strength (favours B) training [13,21,22,37,38,39,41,46,47,52,54,55,56,60,63,64,66,69,70,72]; (C) strength (favours A) vs. endurance-strength (favours B) training [13,22,41,46,55,63,66,69]. CI—confidence interval; CT—combined training; ET—endurance training; ST—strength training; Std—standard; Std diff—standard differences; Figure S48: Subgroup meta-analysis according to time of the intervention (short (≤12 weeks) vs. long (>12 weeks)) of the effect of training programmes on triglycerides levels: (A) endurance (favours A) vs. strength (favours B) training [13,14,22,41,43,45,46,49,53,55,57,63,65,66,68,69]; (B) endurance (favours A) vs. endurance-strength (favours B) training [13,22,37,38,39,41,46,47,52,54,55,56,60,63,64,66,69,72]; (C) strength (favours A) vs. endurance-strength (favours B) training [13,22,41,46,55,63,66,69]. CI—confidence interval; CT—combined training; ET—endurance training; ST—strength training; Std—standard; Std diff—standard differences; Figure S49: Subgroup meta-analysis comparing the effectiveness of combined training with the same vs. longer duration as endurance and strength training alone on glucose levels: (A) endurance (favours A) vs. endurance-strength (favours B) training [20,22,37,38,39,41,46,48,51,54,58,63,66,69,72]; (B) strength (favours A) vs. endurance-strength (favours B) training [20,22,41,46,51,58,63,66,69]. CI—confidence interval; CT—combined training; ET—endurance training; ST—strength training; Std—standard; Std diff—standard differences; Figure S50: Subgroup meta-analysis comparing the effectiveness of combined training with the same vs. longer duration as endurance and strength training alone on insulin levels: (A) endurance (favours A) vs. endurance-strength (favours B) training [20,38,39,41,42,46,48,51,58,63,66,72]; (B) strength (favours A) vs. endurance-strength (favours B) training [20,41,46,51,58,63,66]. CI—confidence interval; CT—combined training; ET—endurance training; ST—strength training; Std—standard; Std diff—standard differences; Figure S51: Subgroup meta-analysis comparing the effectiveness of combined training with the same vs. longer duration as endurance and strength training alone on glycated haemoglobin levels: (A) endurance (favours A) vs. endurance-strength (favours B) training [22,37,38,39,46,48,63,69,70,74]. CI—confidence interval; CT—combined training; ET—endurance training; Std—standard; Std diff—standard differences; Figure S52: Subgroup meta-analysis comparing the effectiveness of combined training with the same vs. longer duration as endurance and strength training alone on homeostatic model assessment of insulin resistance index: (A) endurance (favours A) vs. endurance-strength (favours B) training [20,22,37,38,39,41,46,48,51,55,58,63,69,72]; (B) strength (favours A) vs. endurance-strength (favours B) training. CI—confidence interval; CT—combined training; ET—endurance training; ST—strength training [20,22,41,46,51,55,58,63,69]; Std—standard; Std diff—standard differences; Figure S53: Subgroup meta-analysis comparing the effectiveness of combined training with the same vs. longer duration as endurance and strength training alone on total cholesterol levels: (A) endurance (favours A) vs. endurance-strength (favours B) training [21,22,37,38,41,46,47,52,54,55,56,60,63,64,66,69,70,72];. CI—confidence interval; CT—combined training; ET—endurance training; Std—standard; Std diff—standard differences.; Figure S54: Subgroup meta-analysis comparing the effectiveness of combined training with the same vs. longer duration as endurance and strength training alone on low-density lipoprotein cholesterol levels: endurance (favours A) vs. endurance-strength (favours B) training [21,37,38,41,46,47,52,54,55,56,60,63,64,66,70]. CI—confidence interval; CT—combined training; ET—endurance training; Std—standard; Std diff—standard differences; Figure S55: Subgroup meta-analysis comparing the effectiveness of combined training with the same vs. longer duration as endurance and strength training alone on high-density lipoprotein cholesterol levels: (A) endurance (favours A) vs. endurance-strength (favours B) training [13,21,22,37,38,39,41,46,47,52,54,55,56,60,63,64,66,69,70,72]; (B) strength (favours A) vs. endurance-strength (favours B) training [13,22,41,46,55,63,66,69]. CI—confidence interval; CT—combined training; ET—endurance training; ST—strength training; Std—standard; Std diff—standard differences; Figure S56: Subgroup meta-analysis comparing the effectiveness of combined training with the same vs. longer duration as endurance and strength training alone on triglycerides levels: (A) endurance (favours A) vs. endurance-strength (favours B) training [13,21,22,37,38,39,41,46,47,52,54,55,56,60,63,64,66,69,70,72]; (B) strength (favours A) vs. endurance-strength (favours B) training [13,22,41,46,55,63,66,69]. CI—confidence interval; CT—combined training; ET—endurance training; ST—strength training; Std—standard; Std diff—standard differences; Table S1: PRISMA checklist; Table S2: Methods of unit conversion.
