# Supplementary material for: Comparison of the Effect of Endurance, Strength and Endurance-Strength Training on Glucose and Insulin Homeostasis and the Lipid Profile of Overweight and Obese Subjects: A Systematic Review and Meta-Analysis
Source: Int J Environ Res Public Health. 2022 Nov 13;19(22):14928. doi: 10.3390/ijerph192214928 (PMC9690009; doi:10.3390/ijerph192214928)
Supplement: Supplementary file 1 [file ijerph-19-14928-s001.zip › Table S2.pdf]

Table S2. Methods of unit conversion.

Biochemical parameter	Conversion
Glucose [mmol/l]	1 mg/dl = 0.556 mmol/l
Insulin [µU/ml]	1 pmol/l = 0.144 µU/ml
HbA1c [%]	1 mmol/l = 2.2%
C-peptide [nmol/l]	1 ng/ml = 0.3311 nmol/l
TC, LDL-C, HDL-C [mmol/l]	1 mg/dl = 0.03 mmol/l
TG [mmol/l]	1 mg/dl = 0.0113 mmol/l

HbA1c – glycated haemoglobin; HDL-C – high-density lipoprotein cholesterol; LDL-C – low-density lipoprotein cholesterol; TC – total cholesterol; TG - triglycerides.
