# Supplementary figures and images for: Comparison of the Effect of Endurance, Strength and Endurance-Strength Training on Glucose and Insulin Homeostasis and the Lipid Profile of Overweight and Obese Subjects: A Systematic Review and Meta-Analysis
Source: Int J Environ Res Public Health. 2022 Nov 13;19(22):14928. doi: 10.3390/ijerph192214928 (PMC9690009; doi:10.3390/ijerph192214928)

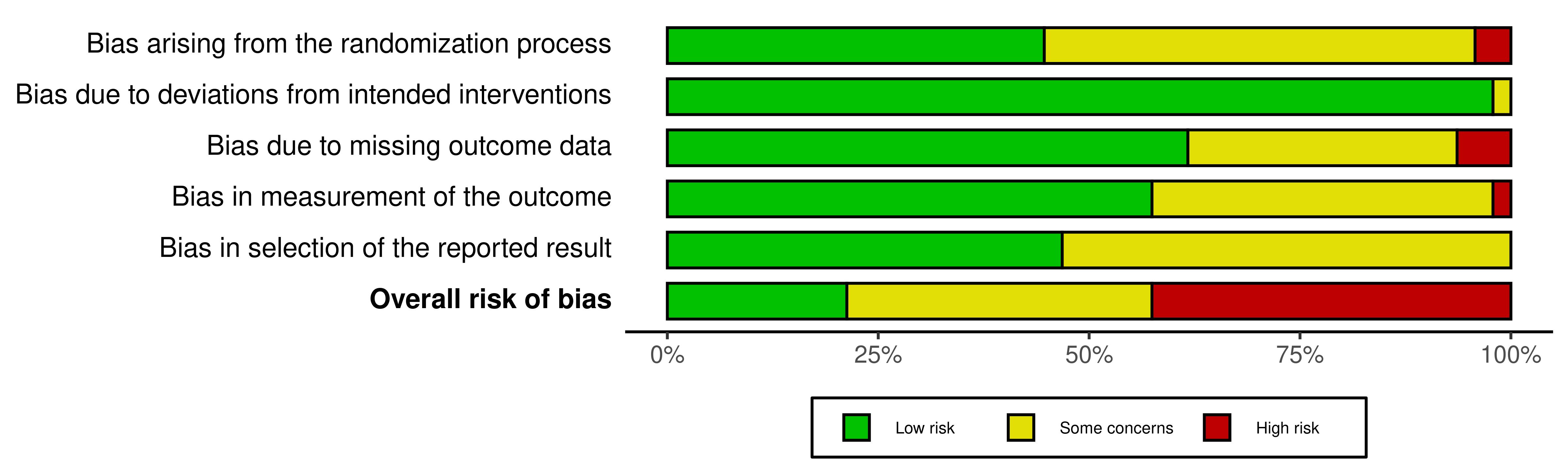

Supplement: Supplementary file 1 [file ijerph-19-14928-s001.zip › Figure S10.jpeg]

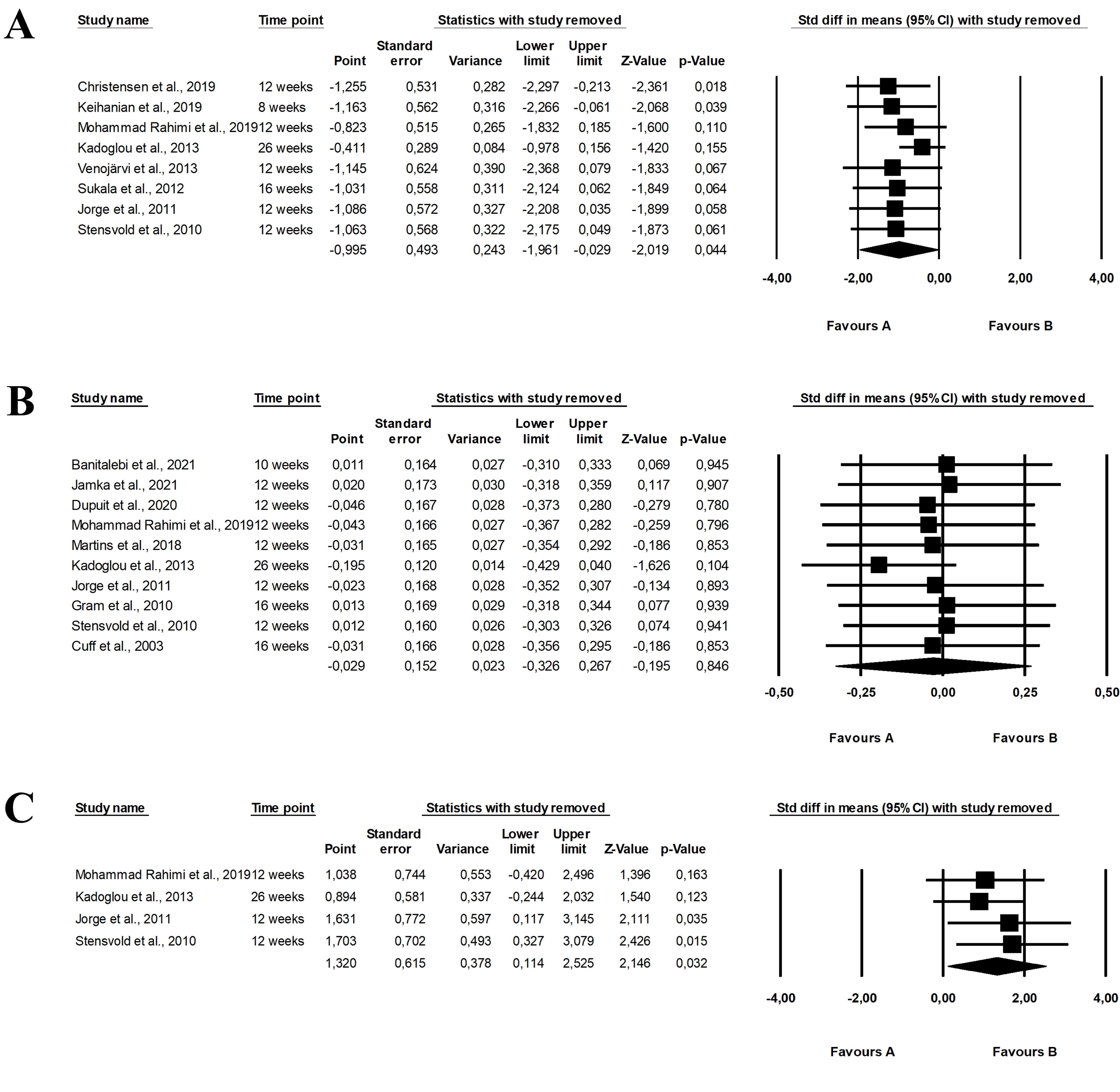

Supplement: Supplementary file 1 [file ijerph-19-14928-s001.zip › Figure S13.jpg]

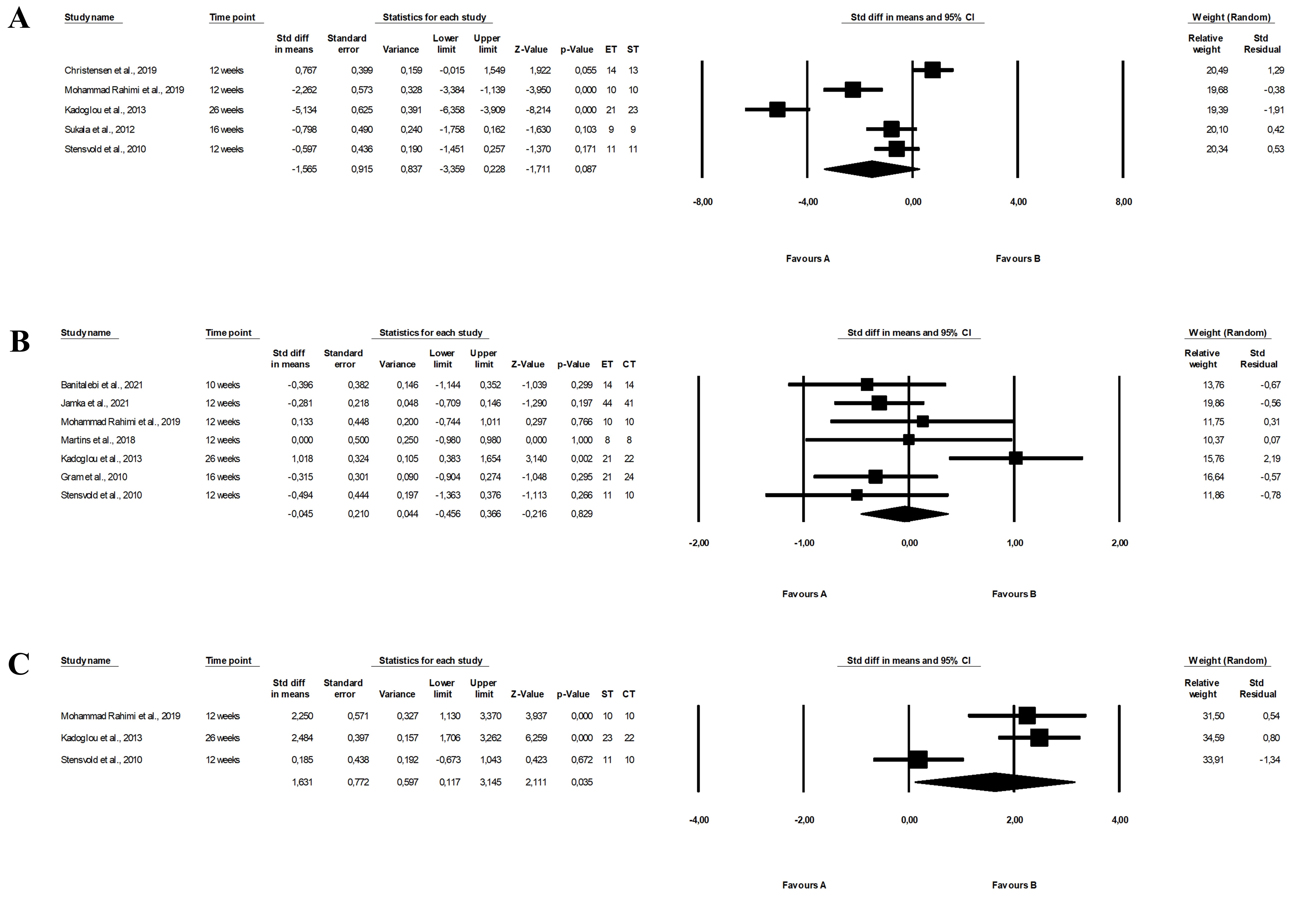

Supplement: Supplementary file 1 [file ijerph-19-14928-s001.zip › Figure S24.jpg]

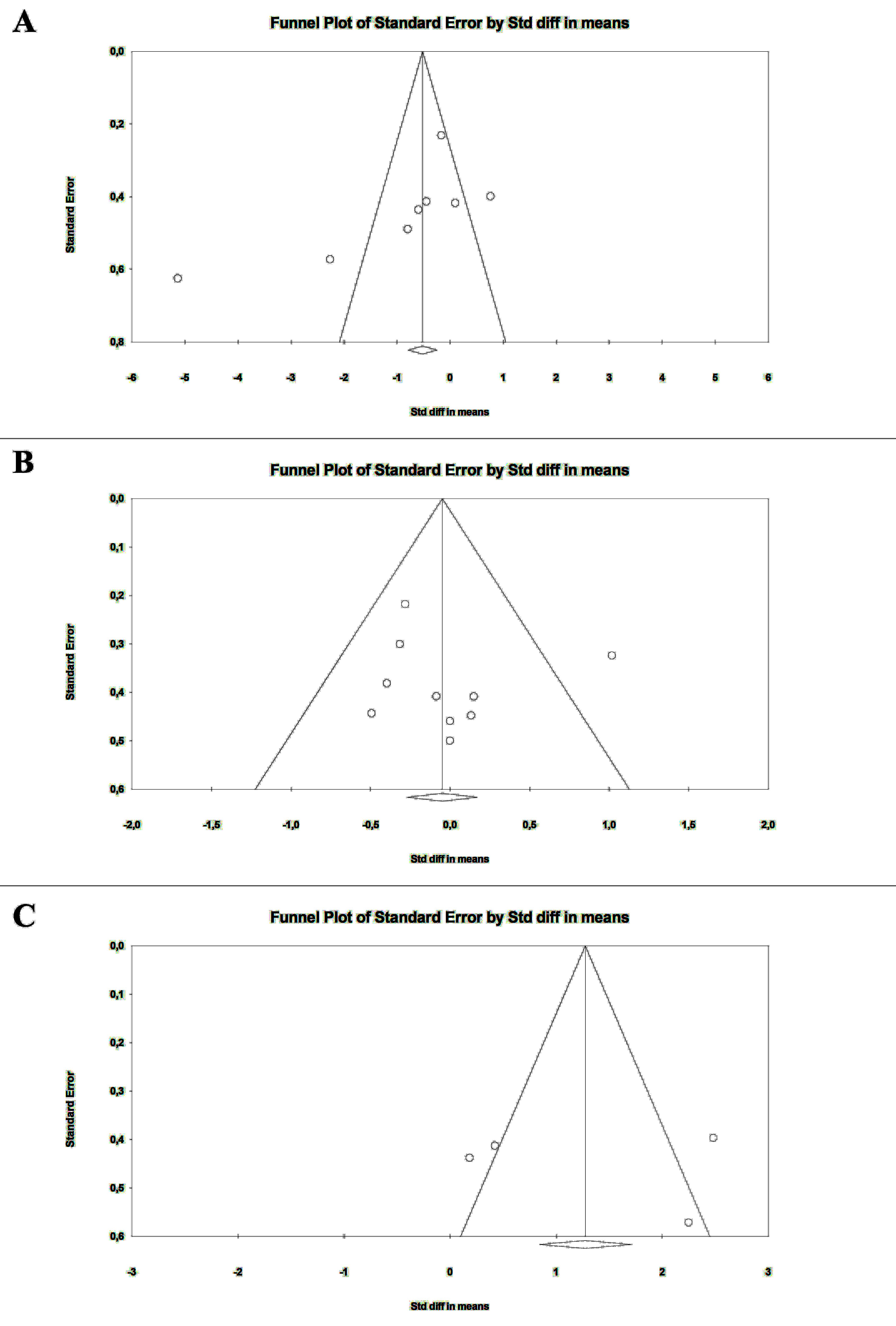

Supplement: Supplementary file 1 [file ijerph-19-14928-s001.zip › Figure S3.jpg]

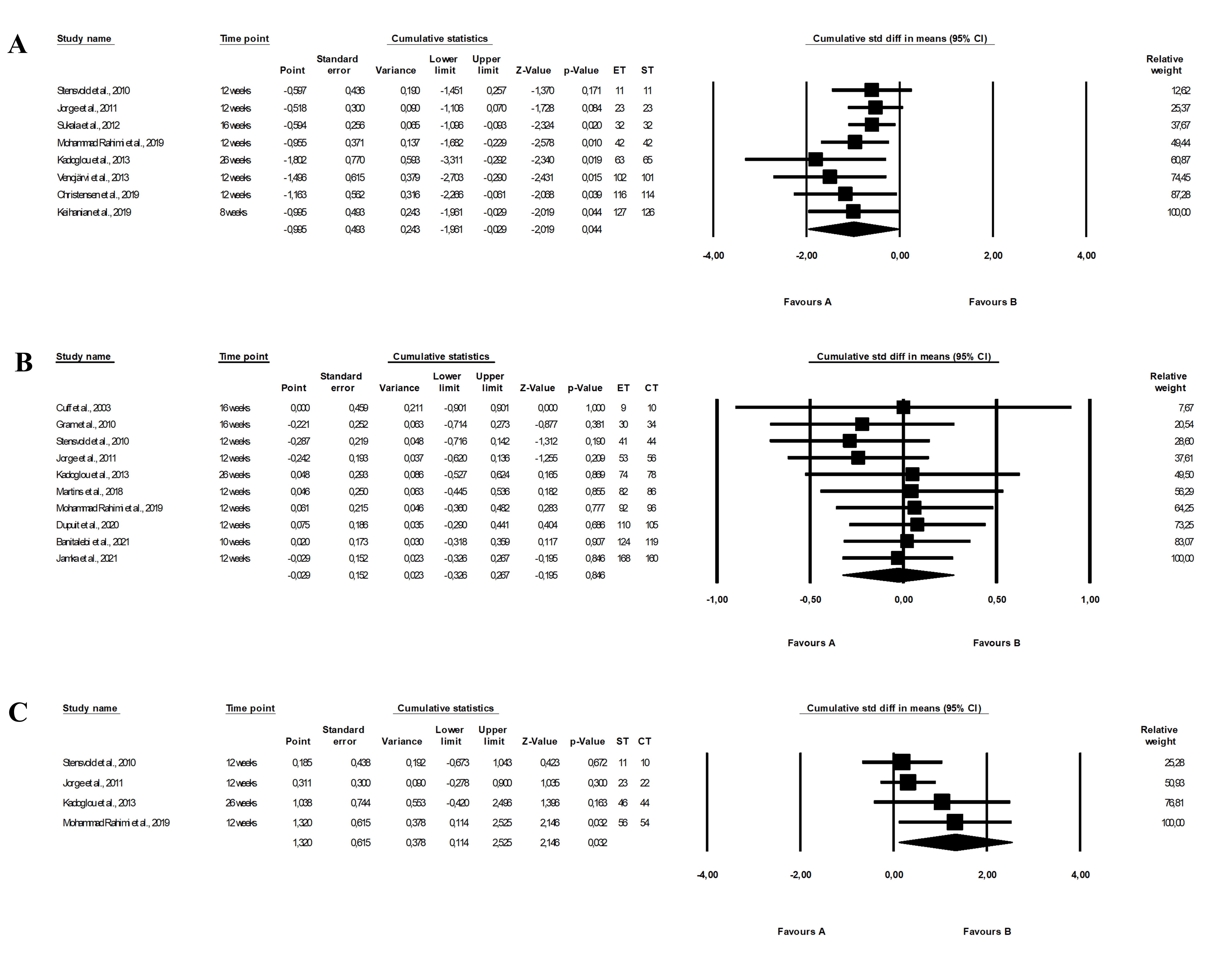

Supplement: Supplementary file 1 [file ijerph-19-14928-s001.zip › Figure S32.jpg]

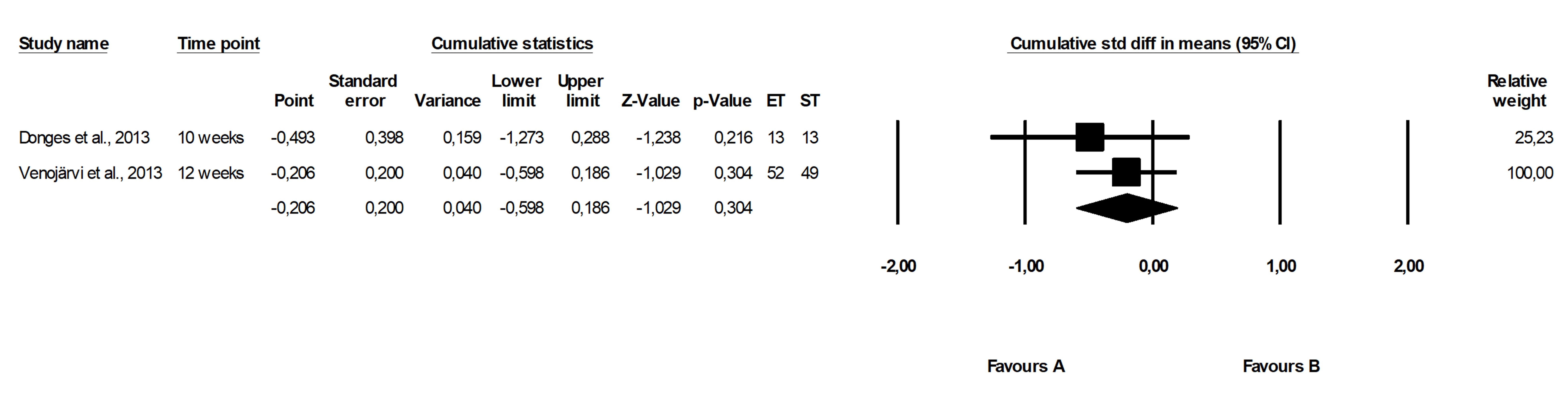

Supplement: Supplementary file 1 [file ijerph-19-14928-s001.zip › Figure S34.jpg]

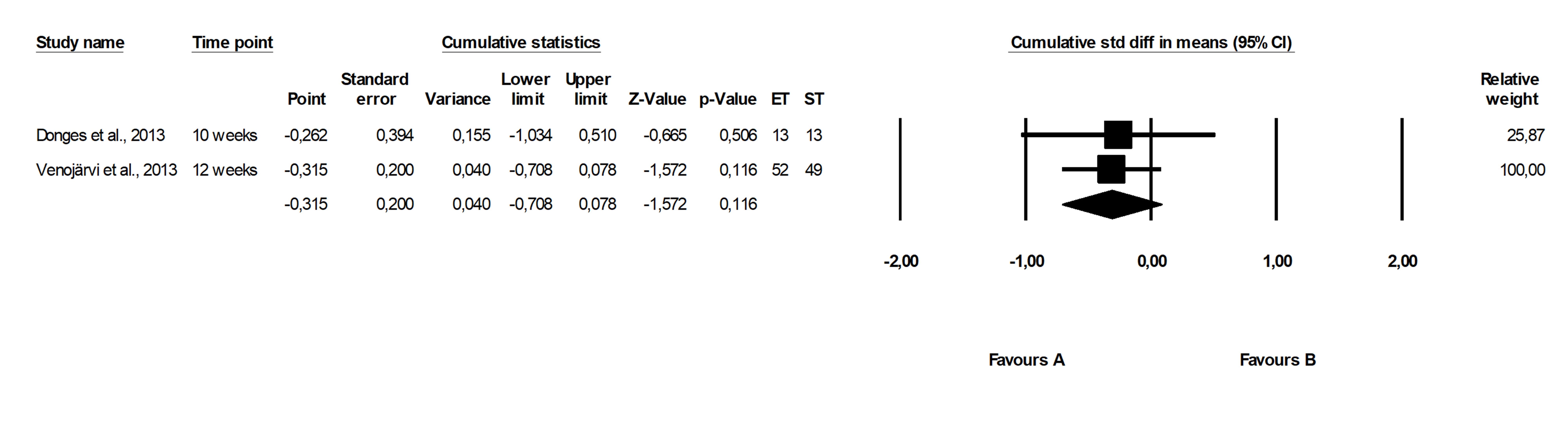

Supplement: Supplementary file 1 [file ijerph-19-14928-s001.zip › Figure S35.jpg]

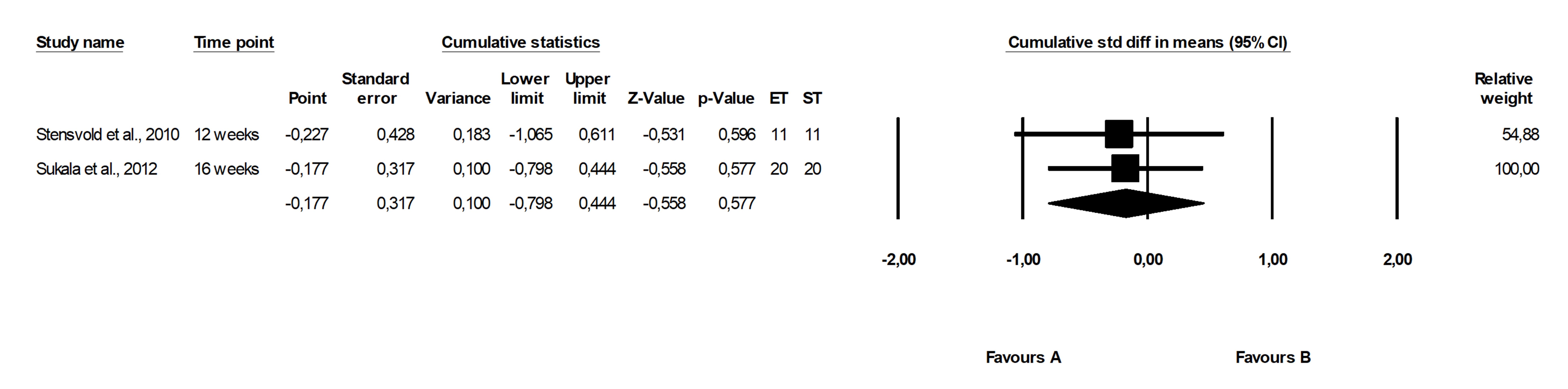

Supplement: Supplementary file 1 [file ijerph-19-14928-s001.zip › Figure S36.jpg]

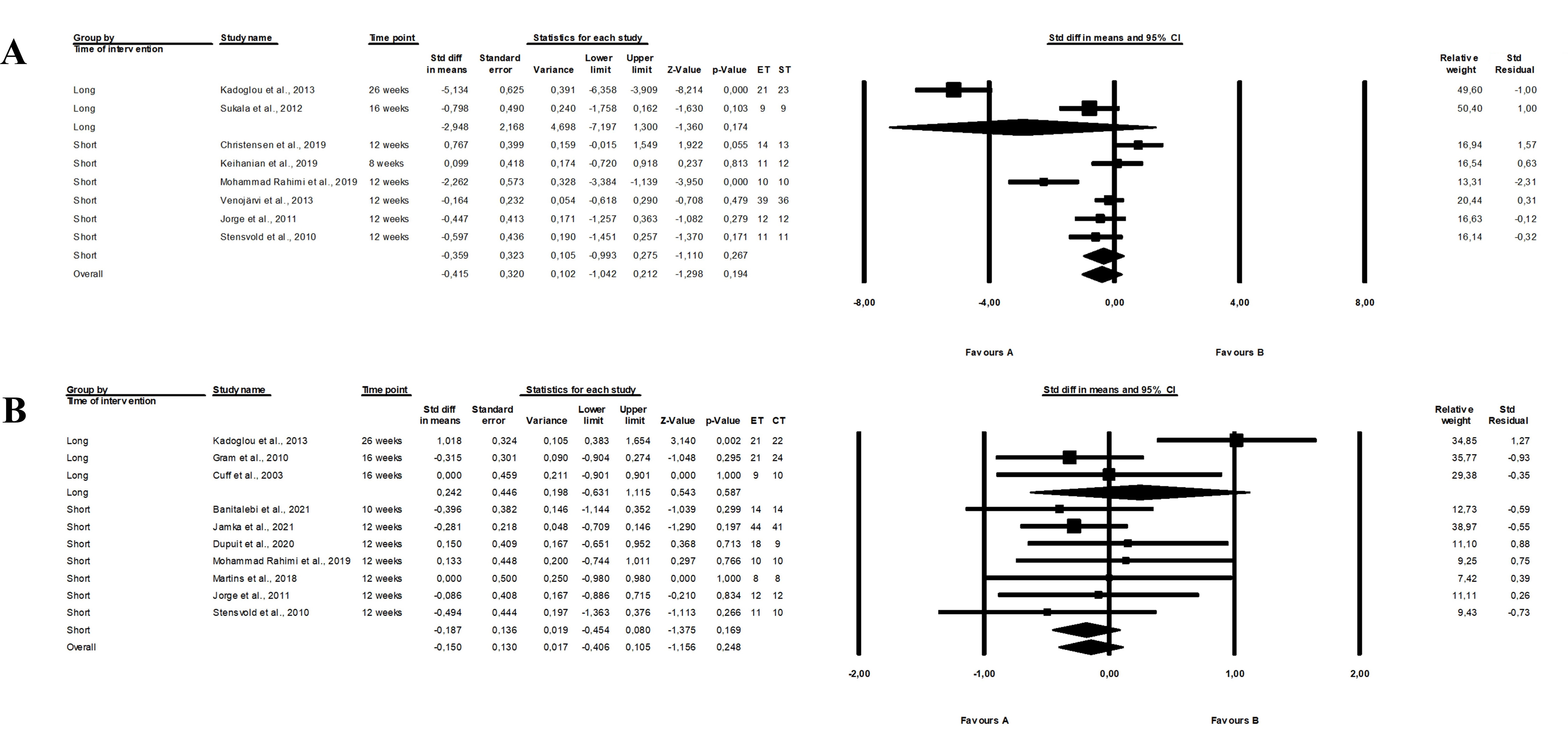

Supplement: Supplementary file 1 [file ijerph-19-14928-s001.zip › Figure S43.jpg]

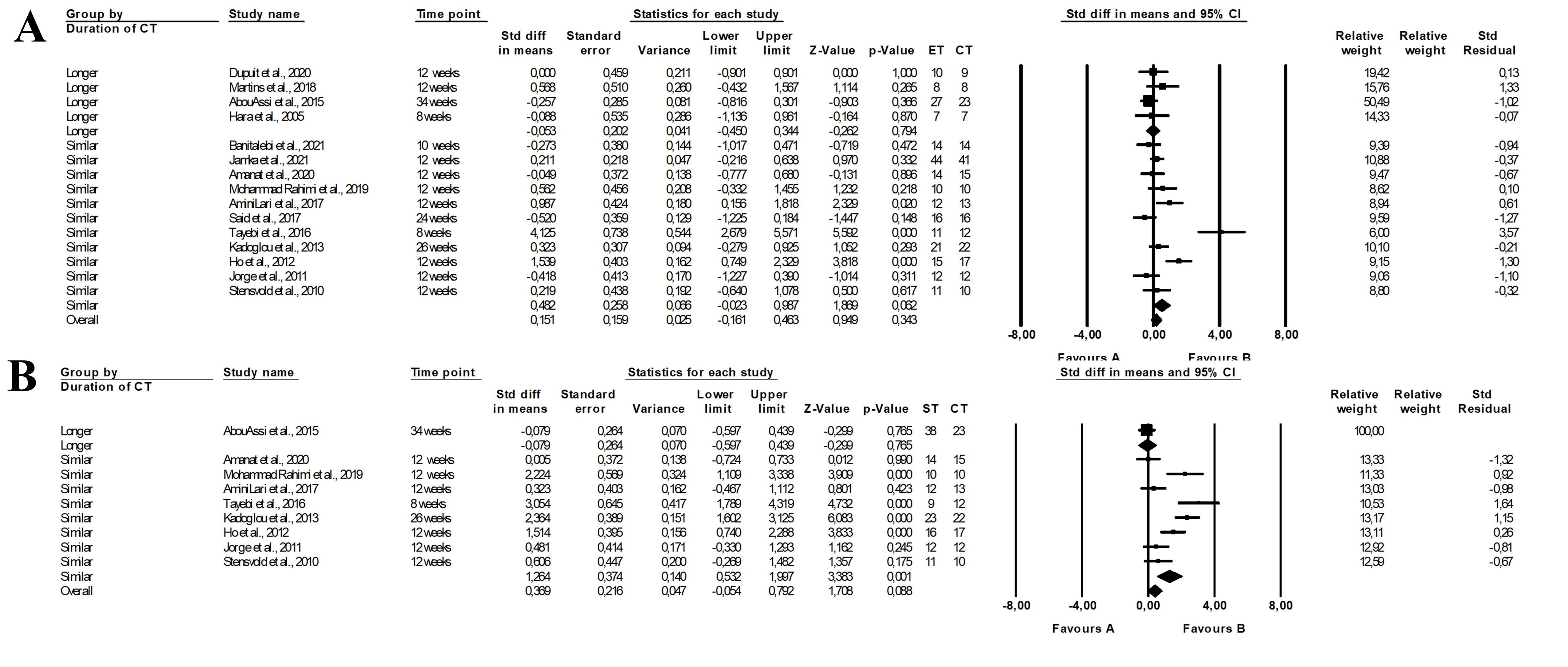

Supplement: Supplementary file 1 [file ijerph-19-14928-s001.zip › Figure S49.jpg]

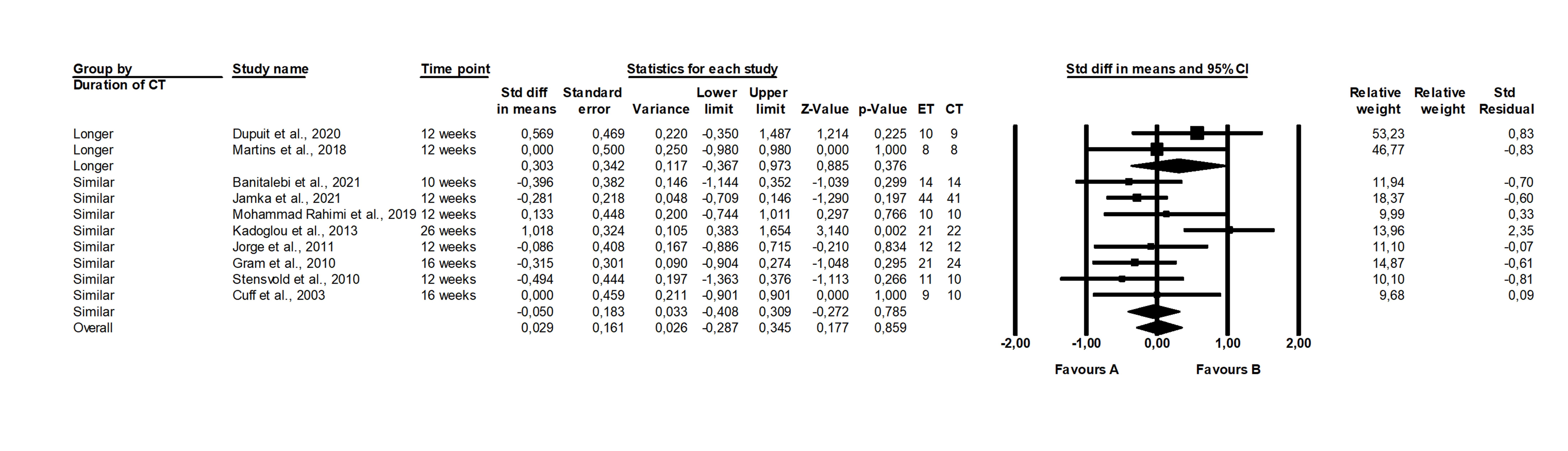

Supplement: Supplementary file 1 [file ijerph-19-14928-s001.zip › Figure S51.jpg]

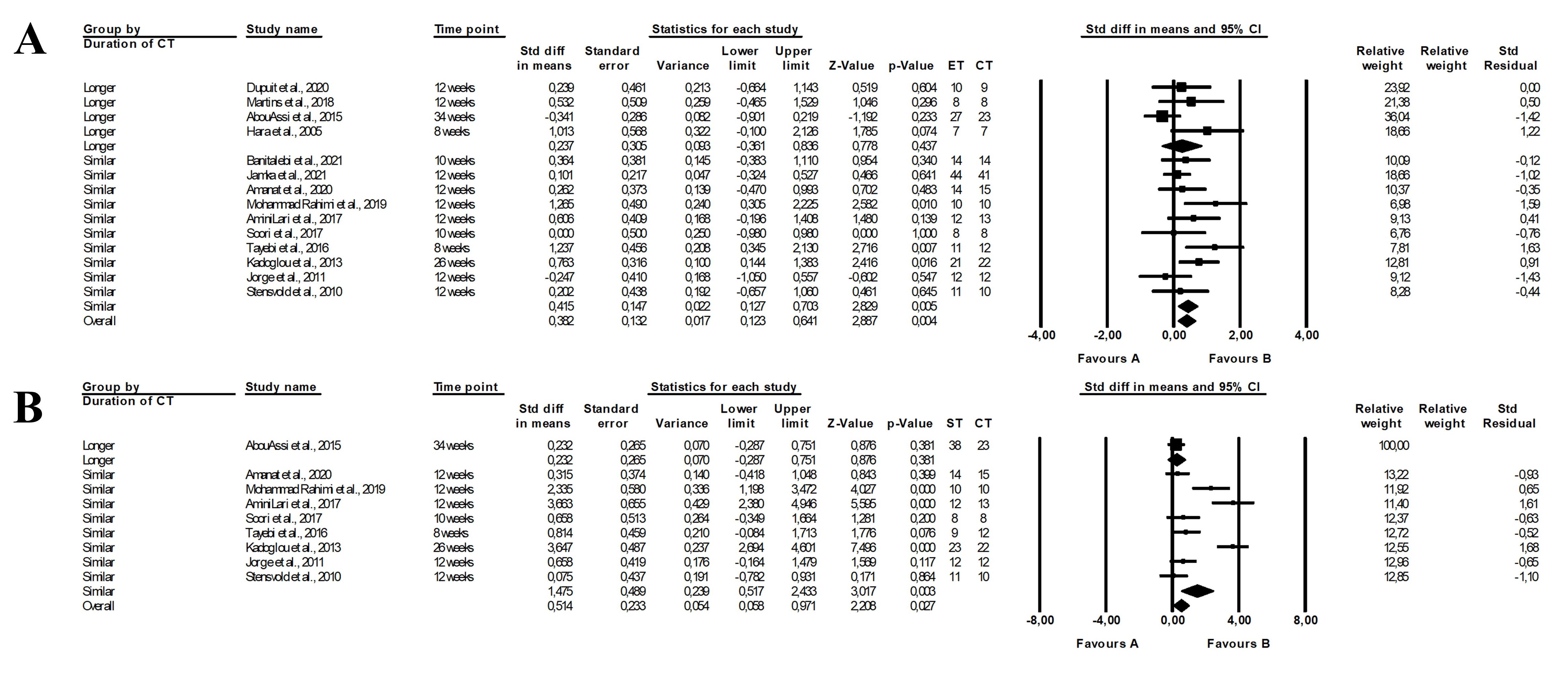

Supplement: Supplementary file 1 [file ijerph-19-14928-s001.zip › Figure S52.jpg]

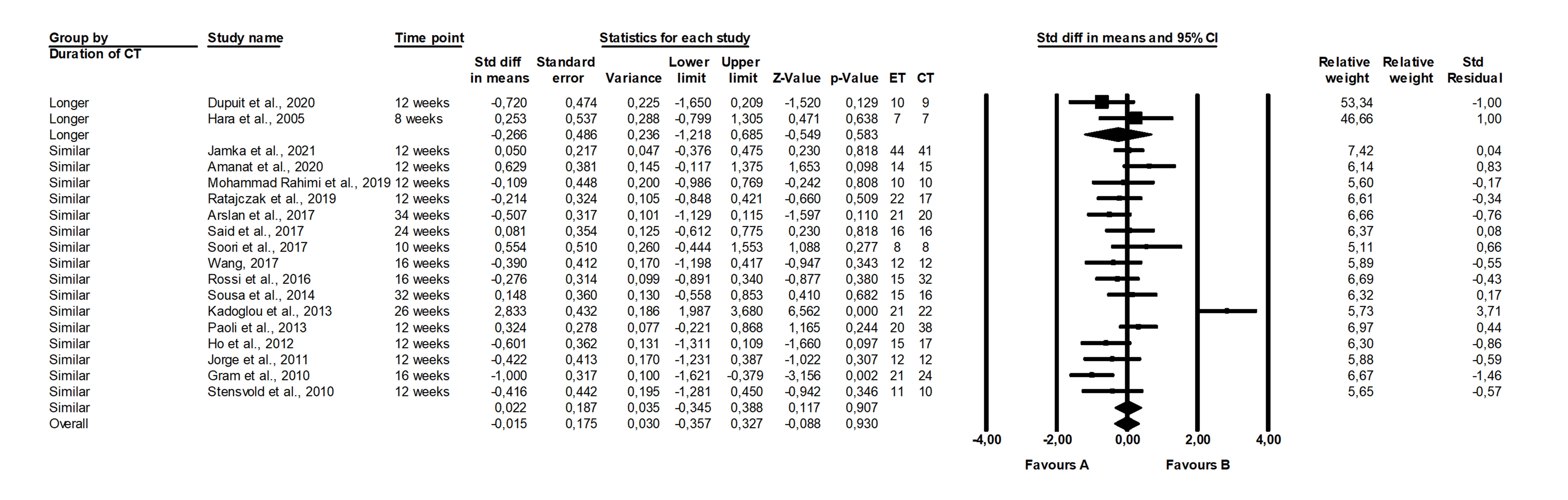

Supplement: Supplementary file 1 [file ijerph-19-14928-s001.zip › Figure S53.jpg]

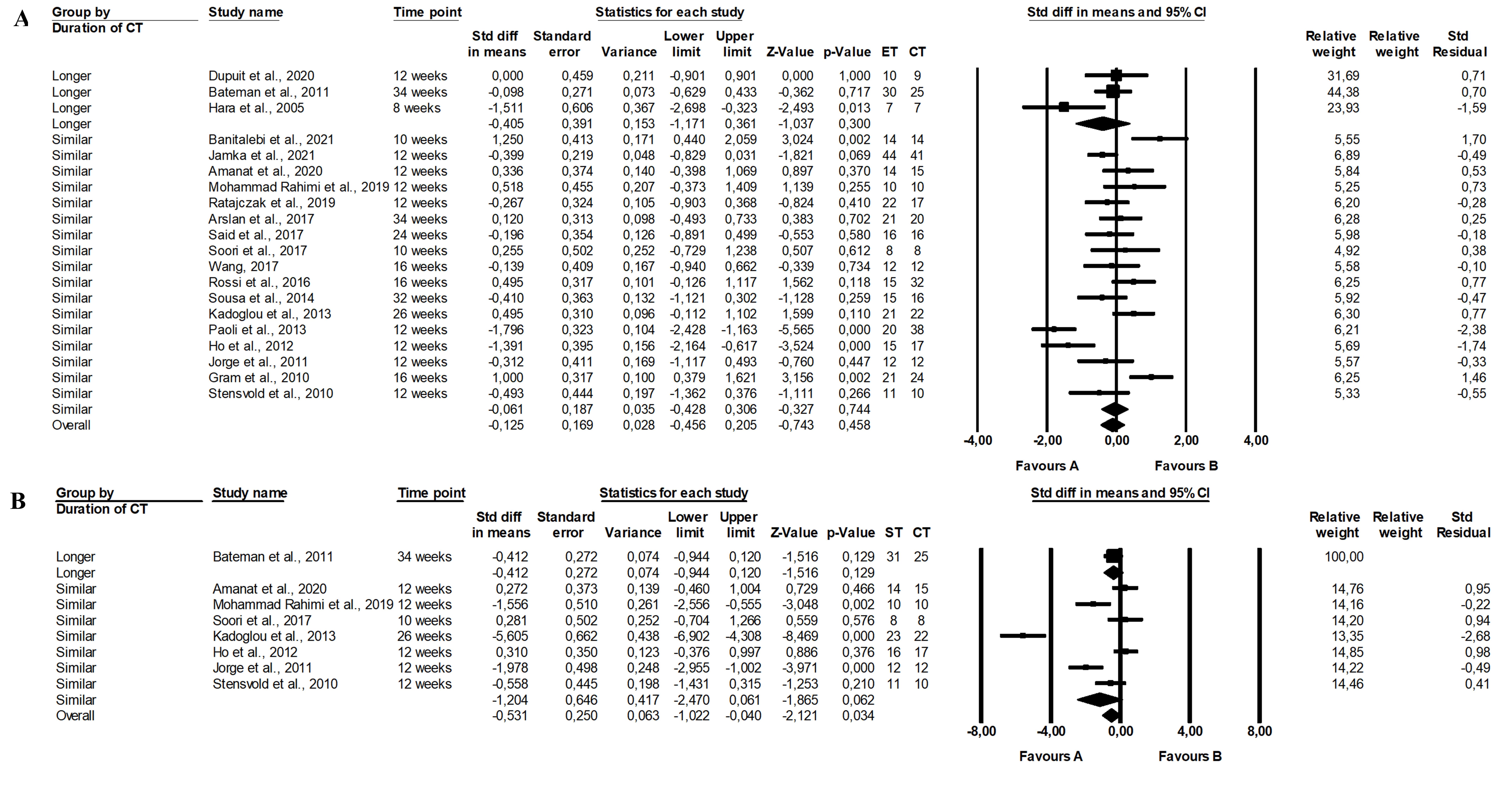

Supplement: Supplementary file 1 [file ijerph-19-14928-s001.zip › Figure S55.jpg]

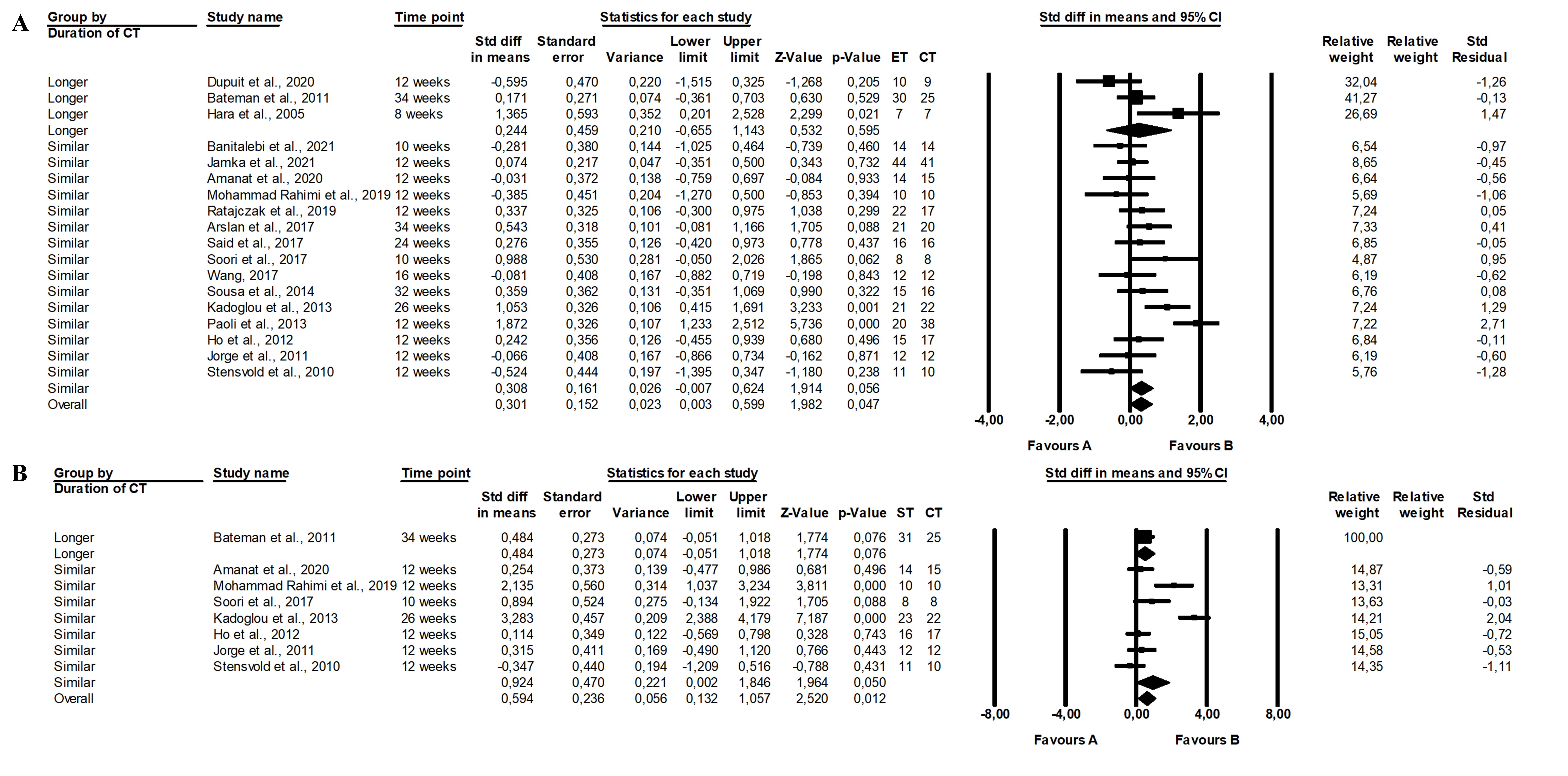

Supplement: Supplementary file 1 [file ijerph-19-14928-s001.zip › Figure S56.jpg]

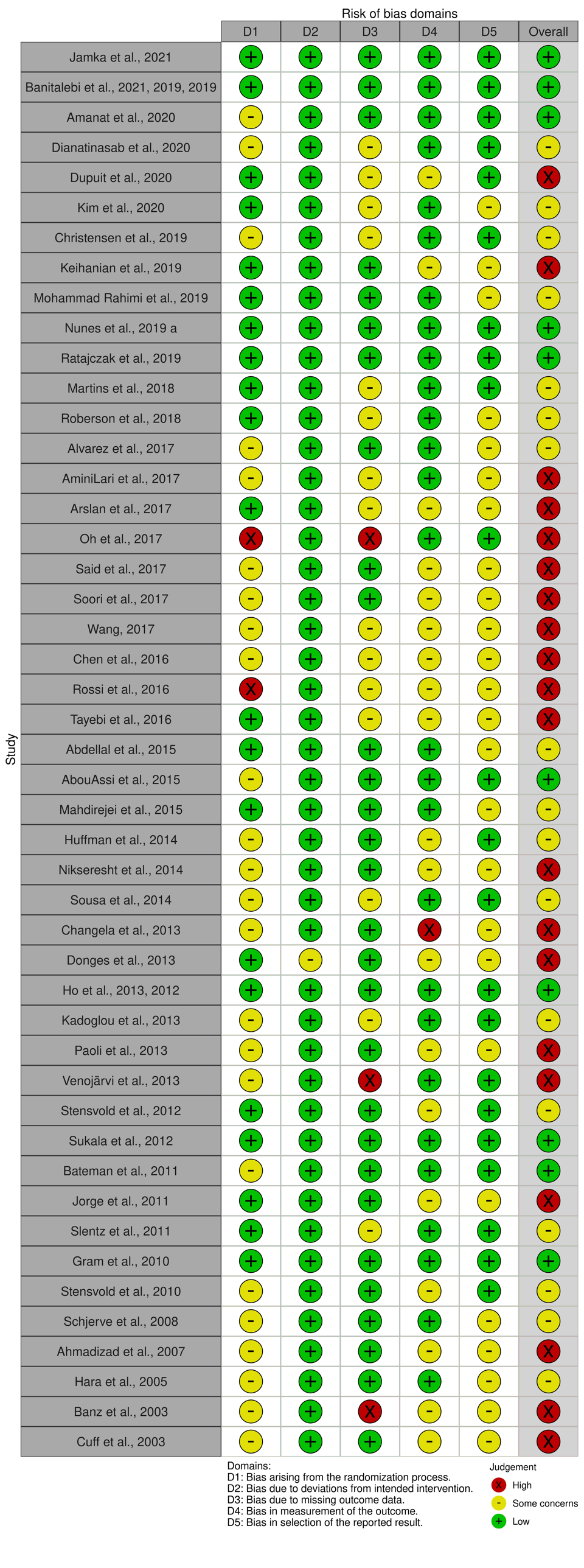

Supplement: Supplementary file 1 [file ijerph-19-14928-s001.zip › Figure S9.jpeg]
